# Developmental Stage-Specific Effects of Parenting on Adolescents’ Emotion Regulation: A Longitudinal Study From Infancy to Late Adolescence

**DOI:** 10.3389/fpsyg.2021.582770

**Published:** 2021-06-04

**Authors:** Jaakko Tammilehto, Raija-Leena Punamäki, Marjo Flykt, Mervi Vänskä, Lotta M. Heikkilä, Jari Lipsanen, Piia Poikkeus, Aila Tiitinen, Jallu Lindblom

**Affiliations:** ^1^Faculty of Social Sciences/Psychology, Tampere University, Tampere, Finland; ^2^Department of Psychology and Logopedics, Faculty of Medicine, University of Helsinki, Helsinki, Finland; ^3^Department of Obstetrics and Gynecology, Faculty of Medicine, University of Helsinki, Helsinki, Finland; ^4^Department of Clinical Medicine, Faculty of Medicine, University of Turku, Turku, Finland

**Keywords:** emotion regulation, sensitive periods, attachment theory, evolutionary–developmental theory, adolescence, parenting

## Abstract

The quality of parenting shapes the development of children’s emotion regulation. However, the relative importance of parenting in different developmental stages, indicative of sensitive periods, has rarely been studied. Therefore, we formulated four hypothetical developmental timing models to test the stage-specific effects of mothering and fathering in terms of parental autonomy and intimacy in infancy, middle childhood, and late adolescence on adolescents’ emotion regulation. The emotion regulation included reappraisal, suppression, and rumination. We hypothesized that both mothering and fathering in each developmental stage contribute unique effects to adolescents’ emotion regulation patterns. The participants were 885 families followed from pregnancy to late adolescence. This preregistered study used data at the children’s ages of 1 year, 7 to 8 years, and 18 years. At each measurement point, maternal and paternal autonomy and intimacy were assessed with self- and partner reports using the Subjective Family Picture Test. At the age of 18 years, adolescents’ reappraisal and suppression were assessed using the Emotion Regulation Questionnaire and rumination using the Cognitive Emotion Regulation Questionnaire. Stage-specific effects were tested comparing structural equation models. Against our hypotheses, the results showed no effects of mothering or fathering in infancy, middle childhood, or late adolescence on adolescents’ emotion regulation patterns. The results were consistent irrespective of both the reporter (i.e., self or partner) and the parental dimension (i.e., autonomy or intimacy). In addition to our main results, there were relatively low agreement between the parents in each other’s parenting and descriptive discontinuity of parenting across time (i.e., configural measurement invariance). Overall, we found no support for the stage-specific effects of parent-reported parenting in infancy, middle childhood, or late adolescence on adolescents’ emotion regulation. Instead, our findings might reflect the high developmental plasticity of emotion regulation from infancy to late adolescence.

## Introduction

Late adolescence, spanning from age 17 to age 24, is a critical developmental stage in which adolescents face novel challenges, including engagement in romantic and sexual relationships, identity exploration, and heightened responsibilities. Coping with these developmental tasks requires efficient management of emotions. *Emotion regulation (ER)* ability allows adolescents to modify the quality, intensity, duration, and expression of emotions according to their goals (Gross, 2015). Understanding the origins of adolescents’ ER is crucial for promoting socioemotional adjustment in the transition to adulthood.

Research suggests that *parenting quality* is linked to children’s ER development in childhood and adolescence. Parenting, as characterized by both relational intimacy and autonomy, can foster children’s constructive ER, whereas emotional neglect and intrusiveness can predispose children to ER problems (Morris et al., 2017). Intriguingly, current developmental models suggest that early stages from infancy to middle childhood may contain unique *sensitive periods* during which long-term ER development is exceptionally responsive to experiences within the parent–child relationship (Del Giudice et al., 2011; Gee, 2016; Perry et al., 2017; Szepsenwol and Simpson, 2019). Yet, the role of timing in the parenting effects remains unclear, as long-term studies focusing on parenting across multiple developmental stages are scarce. Therefore, in this prospective study, we formulated and compared four developmental timing models to test the stage-specific legacy of parenting in infancy, middle childhood, and late adolescence on adolescents’ ER.

Adolescents differ greatly in their reliance on different ER strategies, the most studied ones being *reappraisal*, *suppression*, and *rumination*. These adolescents’ ER patterns reflect their habitual ways to modify emotions when a regulatory need arises. In reappraisal, one changes the cognitive interpretations of emotion-eliciting situations, which typically results in increased positive and decreased negative emotions (Gross, 2015). In suppression, one inhibits the expressions of emotions and hides them from others, which, however, typically results in decreased positive and maintained negative emotions (Gross, 2015). Finally, in rumination, one repeatedly and rigidly focuses on the situation eliciting negative emotions, which, in turn, typically results in increased negative emotions (Nolen-Hoeksema et al., 2008). While the effectiveness of ER is often context-dependent (Bonanno and Burton, 2013), meta-analytical work has shown that reappraisal is linked to adolescents’ better emotional well-being (Schäfer et al., 2017). In contrast, suppression and especially rumination are linked to adolescents’ greater emotional problems (Schäfer et al., 2017).

Despite the ongoing changes in adolescents’ social relationships, parents remain vital figures who support and guide their children’s ER through the adolescence (Rosenthal and Kobak, 2010). According to the development-cohesion model of family relationships, *parental autonomy* and *intimacy* are two fundamental dimensions of parenting that are important for adolescents’ socioemotional development (Mattejat, 1993; Mattejat and Scholz, 1994). Parental autonomy refers to the amount of a parent’s agency, individuality, and confidence in their relationship with the child, whereas parental intimacy refers to the amount of a parent’s responsiveness, emotional attachment, and proximity in their relationship with the child. These dimensions reflect parents’ mental representations and emotional experiences that guide everyday parenting behaviors across diverse contexts. Parenting characterized by both high autonomy and intimacy has been suggested to constitute an optimal developmental environment that promotes children’s constructive ER, whereas deficits in one or both dimensions indicate a less optimal developmental environment for children’s ER (Mattejat, 1993; Mattejat and Scholz, 1994; McElhaney et al., 2009; Kobak et al., 2015; Kobak et al., 2017).

Empirical research confirms that parental autonomy and intimacy in adolescence are linked with adolescents’ ER patterns. In cross-sectional studies, parental responsiveness and proximity have been associated with adolescents’ greater reappraisal and less suppression (Gresham and Gullone, 2012; Cheung et al., 2019). Parental unresponsiveness and emotional detachment have, in turn, been linked with adolescents’ greater rumination (Ruijten et al., 2011). Consistent with this finding, in one longitudinal study, an emotionally intimate mother–child relationship predicted less rumination among girls 3 years later, at the age of 15 (Gaté et al., 2013). In another longitudinal study, high maternal support of adolescents’ autonomy predicted less suppression 1 year later, at the age of 13 (Brenning et al., 2015). Finally, low parental autonomy and intimacy have predicted adolescents’ greater emotional problems in midadolescence (Allen et al., 1994), whereas fathers’ undermining of adolescent autonomy in midadolescence has predicted more externalizing behavior 10 years later (Allen et al., 2002). In sum, contemporary research suggests that parenting quality in terms of autonomy and intimacy can shape adolescents’ ER patterns. However, the basis for these ER patterns may have already been formed during earlier developmental stages within the parent–child relationship.

During infancy and middle childhood, parents serve key regulatory functions in children’s emotional life. Although their behavioral manifestations differ from adolescence, high parental autonomy and intimacy capture the features of high parenting quality also in these early developmental stages, reflecting a supportive, responsive, and non-intrusive developmental environment (Ryan et al., 2016). In infancy, children use parents as their primary source of protection. When parents demonstrate high relational intimacy and autonomy, for example, by maintaining physical proximity and recognizing and responding to an infant’s signals accurately, the infant can feel safe and protected (Cassidy, 1994; Ryan et al., 2016). This felt security further enables infants’ developing sense of agency and encourages exploration (Cassidy, 1994). In middle childhood, parents remain children’s primary source of protection and security (Seibert and Kerns, 2009). Yet, due to children’s increased autonomy and self-regulation skills, parents’ availability when needed, rather than physical proximity, becomes a more critical regulatory factor (Gee et al., 2014; Brumariu, 2015).

The attachment theory helps explain the significance of parenting quality in children’s ER development. The attachment system is an evolutionary-based motivational system that drives children to seek proximity and protection from their parents (Bowlby, 1982). It encodes information from the parent–child relationship to adapt children’s emotional, cognitive, and behavioral patterns to the characteristics of their parent (Szepsenwol and Simpson, 2019). Importantly, within the parent–child relationship, a parent and a child co-regulate the child’s emotions and physiological stress responses, with potential life-span consequences on ER (Cassidy, 1994; Hostinar et al., 2014; Szepsenwol and Simpson, 2019). Recurring experiences with an emotionally intimate and autonomous parent enhance the child’s likelihood to develop a secure attachment with positive internal working models about self and others (de Wolff and van IJzendoorn, 1997; Koehn and Kerns, 2018). These models consist of high self-efficacy in dealing with distress and trust in the supportiveness of others, which together promote the development of constructive ER patterns, such as greater reliance on reappraisal (Cassidy, 1994; Cooke et al., 2019; Mikulincer and Shaver, 2019).

In contrast, if parenting is characterized by rejection, intrusiveness, or inconsistency, a child’s needs for proximity and protection are thwarted, and they are prone to develop an insecure attachment with negative internal working models about self and others (de Wolff and van IJzendoorn, 1997; Koehn and Kerns, 2018). Children with insecure attachment tend to form an ER pattern characterized by either (a) deactivating strategies, such as suppression, that minimize emotions and the sense of neediness or (b) hyperactivating strategies, such as rumination, that exaggerate negative emotions and distress (Cassidy, 1994; Cooke et al., 2019). These ER patterns help to maintain proximity to unresponsive or intrusive parents (Cassidy, 1994). Despite the adaptivity within the parent–child relationship, the insecure ER patterns may generalize to other life contexts and, as a trade-off, increase children’s risk for emotional problems later in life (Mikulincer and Shaver, 2019).

The importance of parenting quality for long-term ER development has been emphasized in infancy due to infants’ high dependence on parents and heightened developmental plasticity, that is, the ability to adjust development based on experience (Frankenhuis and Fraley, 2017, p. 142). Indeed, as children’s *a priori* expectations and beliefs about the world increase during development, plasticity should decrease over time, and the earlier experiences should thus have a disproportionate effect on ER development. In line with this, one long-term study (using partly the same data as the current study) showed that high parental autonomy and, tentatively, intimacy in infancy predict children’s more efficient ER in middle childhood (Lindblom et al., 2016). However, neurodevelopmental research has implied that also during middle childhood, parents still act as critical buffers for children’s neurobiological ER systems, including fronto-amygdala circuits (Gee et al., 2014) and stress response systems (Hostinar et al., 2015). Consequently, recent neurodevelopmental models propose that children’s long-term ER development can be highly sensitive to the experiences within the parent–child relationship not only in infancy but also in middle childhood (Gee, 2016; Perry et al., 2017).

The evidence from the longitudinal and neurodevelopmental studies also concurs with the evolutionary–developmental theory. According to this theoretical framework, both infancy and middle childhood are potential sensitive periods during which ER systems are susceptible to reorganize based on experiences in developmental environments and genetic information (Del Giudice et al., 2011; Del Giudice, 2018). This evolutionary adaptive process may leave a long-lasting developmental mark on children’s ER patterns (Szepsenwol and Simpson, 2019). Differences in parenting should contribute to the differences in the organization of ER systems because the parent–child relationship forms one of the most proximal developmental environments across childhood. Parenting can also provide reliable cues about broader ecological contexts that children will face in their lives and, thus, guide the development of children’s ER systems to function in these contexts later in life (Szepsenwol and Simpson, 2019).

From the attachment, neurodevelopmental, and evolutionary standpoints, it is surprising that only two long-term studies have examined the role of early parenting quality on children’s later ER patterns. In a recent small-sample (*N* = 102) study, a secure mother–child attachment relationship during infancy predicted less deactivating ER, including suppression, in conflict situations with partners in adulthood (Girme et al., 2020). In another study with a larger sample (*N* = 337), maternal intrusiveness at preschool age predicted adolescents’ greater rumination at the age of 13 to 15 years (Hilt et al., 2012). Despite the scarcity of research, these two long-term studies tentatively suggest that early parenting may have long-term effects on children’s later ER patterns.

However, assessing the parenting quality only in one developmental stage leaves several open questions on the essence of the potential association between early parenting and later ER. First, the *relative contributions* of parenting in different developmental stages remain entirely unclear. Second, parenting seems to be relatively stable across development from early to later stages (Fraley et al., 2013). Thus, instead of early parenting quality having long-lasting effects on ER patterns, the *temporal stability* of parenting may explain the observed long-term associations between early parenting quality and later ER patterns. Interestingly, maternal responsiveness and individuality in the first 3 years of life seem to predict adolescents’ lower emotional problems beyond the temporal stability of parenting across time (Haltigan et al., 2013). To date, however, such studies on children’s ER are lacking. If sensitive periods, during which parenting quality has a disproportionate impact on ER, exist, the parenting in these periods should show unique predictive power over and above (a) its temporal stability and (b) parenting in other developmental periods. Therefore, in the current study, we considered these factors while testing whether infancy and middle childhood function as sensitive periods for the long-term effects of parenting quality on adolescents’ ER patterns.

Moreover, the two previous long-term studies regarding the role of early parenting quality for children’s later ER patterns focused only on mothers while ignoring the role of fathers (Hilt et al., 2012; Girme et al., 2020). The same applies to most research on children’s ER development in general (Morris et al., 2017). Nonetheless, both mothering and fathering are likely to shape children’s ER development uniquely. According to Paquette (2004), children tend to seek intimacy and comfort more from their mothers; fathers, in turn, activate children more often and promote their autonomy in overcoming emotional challenges. In line with this view of unique roles, in one longitudinal study on midadolescents, elevated maternal supportiveness predicted increased constructive ER (i.e., flexible impulse control) among girls, whereas elevated paternal behavioral control predicted decreased constructive ER among boys and girls (Van Lissa et al., 2019). In the current study, we focused on both mothers and fathers to increase understanding about the role of parents’ gender in children’s ER development.

## The Current Study

The aim of this study was to examine the relative importance of parenting quality in distinct developmental stages on adolescents’ ER. To achieve this, we formulated four hypothetical models to test the stage-specific effects of mothering and fathering in infancy, middle childhood, and late adolescence on adolescents’ ER patterns. We operationalized parenting quality as mothers’ and fathers’ autonomy and intimacy in relation to their child, perceived by both parents. Focus on these fundamental parental dimensions allowed us to use the same assessment method in each developmental stage. Further, by using multiple informants, we were able to consider both internal (self-perceptions) and external (partner-perceptions) aspects of parenting. Adolescents’ ER patterns were, in turn, operationalized using adolescents’ reports on their habitual use of reappraisal, suppression, and rumination.

**FIGURE 1:**
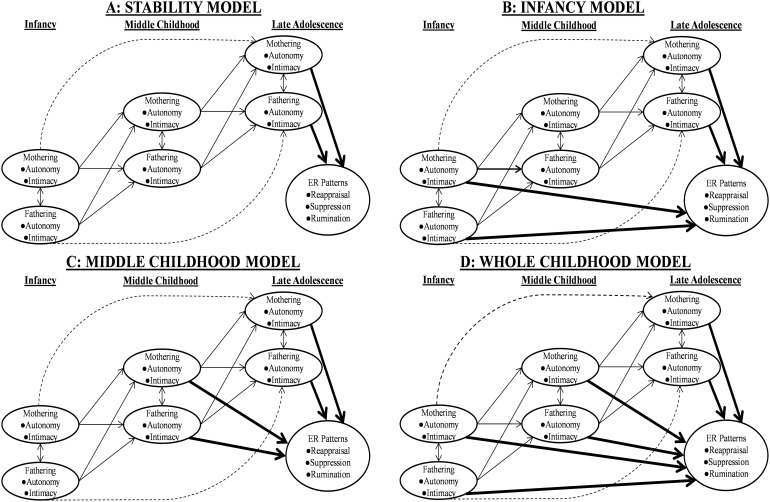
The Hypothetical Developmental Timing Models, including the Stability Model **(A)**, Infancy Model **(B)**, Middle Childhood Model **(C)**, and Whole Childhood Model **(D)**. The bolded paths represent the paths of our main interests. The non-bolded unbroken paths represent the first-order autoregressive and cross-lagged paths and concurrent correlations of mothering and fathering. The dotted paths represent the potential second-order temporal stability paths of the mothering and fathering. ER = emotion regulation.

The four *developmental timing models* are presented in Figure 1. In all models, parenting quality in late adolescence contributes to adolescents’ ER patterns. Yet, the models differ in the long-term effects of parenting quality in infancy and middle childhood on adolescents’ ER patterns. In the first model, the *Stability Model* (Figure 1A), parenting quality contributes to adolescents’ ER patterns only in late adolescence. This model conveys that the temporal stability of parenting solely explains the associations of the parenting quality in infancy and middle childhood on adolescents’ ER patterns. In the second model, the *Infancy Model* (Figure 1B), the parenting quality in infancy contributes to adolescents’ ER patterns directly, over and above the temporal stability of parenting. This model conveys that infancy forms the sensitive period in which parenting quality has long-term effects on ER patterns. In contrast, in the third model, the *Middle Childhood Model* (Figure 1C), the parenting quality in middle childhood contributes to adolescents’ ER patterns directly, over and above the temporal stability of parenting. This model conveys that middle childhood forms the sensitive period in which parenting quality has long-term effects on ER patterns. Finally, in the fourth model, the *Whole Childhood Model* (Figure 1D), the parenting quality in both infancy and middle childhood contributes to adolescents’ ER patterns directly, over and above the temporal stability of parenting. This model conveys that both infancy and middle childhood form the sensitive periods in which parenting quality has long-term effects on ER patterns.

We used these models to test four interrelated hypotheses. First, we hypothesized that the parenting quality in both infancy and middle childhood has long-term effects on adolescents’ ER patterns. Thus, we expected that the Whole Childhood Model would be the most suitable model to describe the stage-specific effects of parental autonomy and intimacy on adolescents’ ER patterns. Second, we hypothesized that the parenting quality in late adolescence also independently contributes to adolescents’ ER patterns. Third, we hypothesized that the quality of both mothering and fathering plays a role in adolescents’ ER patterns. Finally, as a directional hypothesis, we hypothesized that high parenting quality in terms of higher parental autonomy and intimacy promotes adolescents’ constructive ER patterns, involving greater reappraisal and less suppression and rumination.

## Materials and Methods

### Participants and Procedure

The study was part of the Miracles of Development research project that has followed 885 Finnish families from pregnancy (mothers: *M*_*age*_ = 33.2 years, range: 20–47; fathers: *M*_*age*_ = 34.5 years, range: 20–58) to children’s age of 17 to 19 years. The original sample consisted of (a) naturally conceiving couples (NC; *n* = 442) recruited at the Helsinki University Central Hospital during a routine ultrasonographic examination and (b) couples who had conceived with assisted reproductive treatment (ART; *n* = 443) recruited from five infertility clinics in Finland. Inclusion criteria were singleton pregnancy, and for the NC, no previous infertility history and maternal age over 25 years. The NC and ART groups did not differ in maternal age, paternal age, child’s sex, and parents’ average education level (*p*s > 0.05). Most parents (66% of the mothers; 53% of the fathers) had at least postsecondary education. Of all children, 399 were boys and 407 girls (79 missing values). The ethical boards of the Helsinki University Hospital and the five infertility clinics approved all phases of data collection.

In the current study, we used data from the children’s age of 1 year (T1), 7 to 8 years (T2), and 17 to 19 years (T3). Mothers and fathers participated at T1, T2, and T3, whereas children participated only at T3. After the data collection at T3, we registered all hypotheses and analysis plan before analyses^1^.

At T1, response rates from the beginning of the research project were 61% for mothers (*n* = 544; *n*_NC_ = 251, *n*_ART_ = 293) and 57% for fathers (*n* = 502; *n*_NC_ = 223, *n*_ART_ = 279). The attrition at T1 was independent of paternal age, maternal age, child’s sex, and parents’ average education level at pregnancy (*p*s > 0.05). At T2, the response rates were 59% for mothers (*n* = 519; *n*_NC_ = 273, *n*_ART_ = 246) and 33% for fathers (*n* = 296; *n*_NC_ = 132, *n*_ART_ = 164). Lower paternal age, maternal age, and parents’ average education level at pregnancy were related to fathers’ attrition. At T3, the response rates were 51% for mothers (*n* = 449; *n*_NC_ = 222, *n*_ART_ = 227), 40% for fathers (*n* = 357; *n*_NC_ = 171, *n*_ART_ = 186), and 49% for adolescents (*n* = 437; *n*_NC_ = 220, *n*_ART_ = 217). Lower parents’ average education level at pregnancy was related to the attrition of mothers, fathers, and adolescents, and lower paternal age was related to fathers’ attrition. Among adolescents, boys’ attrition (*n* = 176) was higher than girls’ attrition (*n* = 261). The number of complete cases (i.e., both parents participated at each assessment and their adolescent participated at T3) was 129, and the numbers of pairwise observations between assessments ranged from 186 to 500. Specifically, the pairwise observations between adolescents’ reports at T3 and parental reports at T1, T2, and T3 ranged from 205 (fathers’ reports at T2) to 357 (mothers’ reports at T3).

### Measures

#### Parenting Quality

At T1, T2, and T3, parenting quality was assessed using the parental autonomy and intimacy subscales of the Subjective Family Picture Test (Mattejat and Scholz, 1994). Using a 7-point (−3 to 3) bipolar scale with two extremes, both mothers and fathers reported their perceptions of maternal and paternal autonomy (four items; e.g., “In relation to my child I am/my partner is indecisive–determined”) and maternal and paternal intimacy (four items; e.g., “In relation to my child I am/my partner is rejecting–loving”). This enabled us to use both self- and partner (or ex-partner) reports to model these parental dimensions. The correlations between self- and partner-reported sum scores at T1, T2, and T3 were modest (*r*_*autonomy*_ = 0.08–0.26; *r*_*intimacy*_ = 0.14–0.30). The items of autonomy (skewness = −2.43–−0.85 and kurtosis = −0.19–10.55) and especially intimacy (skewness = −8.98–−1.17 and kurtosis = 0.80–117.04) were skewed to the left and leptokurtic, implying high autonomy and intimacy in most families. This non-normality of parental autonomy and intimacy items was taken into account in analyses (see Analytic Strategy section). Before analyzing data, the scales of autonomy and intimacy items were transformed to range from 1 to 7.

#### Adolescents’ Emotion Regulation Patterns

At T3, adolescents’ ER patterns were assessed using the Emotion Regulation Questionnaire (ERQ; Gross and John, 2003) and rumination and catastrophizing scales of the Cognitive Emotion Regulation Questionnaire (CERQ; Garnefski et al., 2001). In the ERQ, using a 7-point Likert scale (1 = *strongly disagree* to 7 = *strongly agree*), adolescents reported their use of reappraisal (six items; e.g., “I control my emotions by changing the way I think about the situation I’m in”) and suppression (four items; e.g., “I keep my emotions to myself”) in ER-evoking situations. In the CERQ, using a 5-point Likert scale (1 = *almost never* to 5 = *almost always*), adolescents reported their use of rumination (four items; e.g., “I dwell upon the feelings the situation has evoked in me”) and catastrophizing (four items; e.g., “I continually think how horrible the situation has been”) in unpleasant situations.

Several studies have supported the theoretical two-factor structure of the ERQ (Gross and John, 2003; Gómez-Ortiz et al., 2016). For the CERQ rumination, a study by Ireland et al. (2017) is so far the highest-quality validation study. In that study, two original rumination items (“I often think about how I feel about what I have experienced”; “I want to understand why I feel the way I do about what I have experienced”) showed poor, < 0.30 standardized loadings. According to Ireland et al., these items may measure self-reflection and curiosity rather than rumination. Also, another ER pattern, catastrophizing, correlated strongly with rumination (*r* = 0.92), suggesting that these scales may reflect mostly the same ER pattern (Ireland et al., 2017). As a result, we tested two competing measurement models for the CERQ rumination (see Analytic Strategy section).

#### Covariates

Parents’ average education level at pregnancy (1 = *lower than vocational training* to 4 = *higher education*), child’s sex (0 = *boy*, 1 = *girl*), and ART status (0 = *no*, 1 = *yes*) were included as covariates in the analyses. By including parents’ average education level at pregnancy as a covariate, we aimed to ensure that the effects we were interested in did not depend on the early socioeconomic status of families that seems to play a role in parenting (Conger et al., 2010) as well as children’s ER development (Ursache and Noble, 2016). Moreover, we wanted to take into account the child’s sex in our analyses due to the documented associations between sex and ER patterns, particularly suppression (Gross and John, 2003) and rumination (Hilt et al., 2012). Finally, although no apparent reason existed to expect that ART status would be a cofounder for the effects of parenting quality on adolescents’ ER patterns, we also decided to control for ART status. The aim was to verify that the sample distribution did not play a role in the effects we were interested in.

### Analytic Strategy

Structural equation modeling (SEM) was used as a statistical tool to test the hypotheses. All models were conducted using the *Mplus version 8.3* (Muthén and Muthén, 1998–2019) or the *lavaan* package (Rosseel, 2012) in R software. Maximum likelihood estimation with robust standard errors and the Yuan–Bentler scaled test statistic was used as the estimator due to its robustness for non-normality. Throughout the analyses, parental autonomy and intimacy were examined in separate models to reach a more acceptable parameter-to-*N* ratio. Moreover, the self- and partner reports were handled in separate models, because these were unlikely to measure identical constructs. Yet, mothering and fathering were modeled simultaneously in all models. Thus, we had four separate modeling situations to test our four hypotheses: (a) self-reported parental autonomy, (b) partner-reported parental autonomy, (c) self-reported parental intimacy, and (d) partner-reported parental intimacy. In the self-reported models, we used mother-reported maternal autonomy/intimacy data and father-reported paternal autonomy/intimacy data. In turn, in the partner-reported models, we used father-reported maternal autonomy/intimacy data and mother-reported paternal autonomy/intimacy data. We made two justified changes to the original preregistered analysis plan, which will be described below. All analysis scripts and outputs can be found at https://osf.io/b8mqz.

#### Measurement Models

Before testing the hypotheses with the developmental timing models, the measurement models of parental autonomy and intimacy and ER patterns were assessed with confirmatory factor analysis. The measurement models of parental autonomy and intimacy included six latent variables (maternal and paternal autonomy/intimacy at T1, T2, and T3), with four indicators each. The correlations between the latent variables were estimated. The error term correlations of the corresponding indicators were also estimated across time as we assumed that these indicators shared some unique method variance. The time invariance of mothering and fathering and the factorial invariance between the mothering and fathering were assessed in terms of configural, weak, strong, and strict invariance.

After testing the invariance structure of parental autonomy and intimacy, we fixed the error term correlations of corresponding indicators, which did not weaken the model fit, to the same value or zero in order to minimize the parameter-to-*N* ratio. Then, in each of the four modeling situations, we changed the measurement models of parenting to autoregressive cross-lagged models. We compared two models: (a) the basic first-order autoregressive cross-lagged model and (b) the second-order autoregressive cross-lagged model in which the second-order autoregressive paths (from T1 to T3) of both fathering and mothering were estimated. In the preregistration, we planned to use the second-order structure only if the model fit of the first-order structure did not meet the set criterion, and the second-order structure fit the data better. However, we decided to choose the second-order structure if this improved the model fit regardless of the fit of the first-order model. We viewed this solution as more valid because it decreased the risk to misspecify model parts that did not directly concern our hypotheses. Of these first- and second-order models, we included the model with a better fit as such to the developmental timing models.

The measurement model of the ERQ included a latent reappraisal variable with six indicators and a latent suppression variable with four indicators. The correlation between the latent variables was estimated. Regarding the CERQ rumination, we tested two models due to the results of the validation study by Ireland et al. (2017). In the first original model, we used the four original rumination items as indicators of latent rumination. In the second alternative model, we excluded two rumination items showing poor loadings in Ireland et al.’s study and combined the scales of rumination and catastrophizing that correlated strongly in that study. Thus, in this model, we used the other two rumination items (i.e., “I am preoccupied with what I think and feel about what I have experienced”; “I dwell upon the feelings the situation has evoked in me”) and the four catastrophizing items as indicators of latent rumination.

Then, on the basis of the results regarding the individual models of the ERQ and CERQ rumination, we tested the models for all three ER patterns and estimated Bartlett factor scores for each ER pattern. These factor scores were used as the single indicator variables of latent ER patterns in the developmental timing models. This procedure minimized the parameter-to-*N* ratio in these models, while also allowing us to take into account the measurement error related to ER patterns. Thus, attenuation correction was applied for single indicator latent variables by fixing the loading of each latent ER pattern on its single indicator (i.e., factor score) to 1.00 and the error variance of the single indicator to:

VAR⁢(X)⁢(1-ρ)

where **VAR(X)** was the variance, and ρ was the reliability estimate of the factor score. To estimate the reliabilities of the ER patterns, we computed Tarkkonen’s rho (Tarkkonen and Vehkalahti, 2005).

#### Developmental Timing Models

To test the first hypothesis, we formulated and compared the four developmental timing models in all four self-reported and partner-reported modeling situations (in line with Figure 1). All parameters were specified to vary freely between the modeling situations. In the Stability Model, we estimated only the effects of maternal and paternal autonomy/intimacy at T3 on adolescents’ ER patterns. In the Infancy Model, we estimated the effects of maternal and paternal autonomy/intimacy at T1 and T3 on adolescents’ ER patterns. In the Middle Childhood Model, we estimated the effects of maternal and paternal autonomy/intimacy at T2 and T3 on adolescents’ ER patterns. Finally, in the Whole Childhood Model, we estimated the effects of maternal and paternal autonomy/intimacy at T1, T2, and T3 on adolescents’ ER patterns. In all models, we estimated the path coefficients of all covariates on maternal and paternal autonomy/intimacy at T2 and T3 and adolescents’ ER patterns. Of these models, we selected the best-fitted model for each of the four modeling situations.

Next, we further examined the estimated paths of parental autonomy/intimacy on ER patterns in the selected models. In all four modeling situations, we compared the selected developmental timing models and their submodels in which the effects of parental autonomy/intimacy at T3 on ER patterns were fixed to zero. Comparing the fit of these models allowed us to test our second hypothesis, whether parental autonomy/intimacy in late adolescence had effects on adolescents’ ER patterns. Moreover, we aimed to conduct several other model comparisons to test our third and fourth hypotheses regarding the role of parents’ gender and whether parenting had effects on all or only some ER patterns.

#### Missing Data

Regarding the variables used in our analyses (i.e., indicators of parental autonomy and intimacy and ER patterns and covariates), Little’s MCAR test rejected the missing completely at random assumption, χ^2^ (11389) = 12237.65, *p* < 0.001. As a result, missing data were handled using the full information maximum likelihood estimation (FIML) with auxiliary variables. In the preregistration, we planned to conduct the analyses without the auxiliary variables. Nonetheless, we decided to include the auxiliary variables in models to decrease potential systematic biases in missingness. This corresponds with the current best practices of handling missing data: Using auxiliary variables improves the FIML estimation by increasing the probability of missing at random assumption (i.e., missingness is related to observed variables in the data), the most critical assumption of modern missing data methods such as FIML (Enders, 2010; Howard et al., 2015; Rioux and Little, 2021).

Because our multidisciplinary data included thousands of potential auxiliary variables, we utilized the approach proposed by Howard et al. (2015) to minimize the number of auxiliary variables in models. To form auxiliary variables, in short, we first data-mined the most relevant variables that correlated either with the missingness using *p* < 0.05 as a criterion and/or with the parenting and ER variables used in analyses. In the latter case, we included all variables that showed average |*r*| of > 0.20 with the indicators of the latent parenting and ER variables used in the developmental timing models. However, when less than 10 variables with the average |*r*| > 0.20 were available, we selected the first 10 variables with the highest correlations to maximize relevant information for all variables used in the analyses. Second, using the *semTools* package (Jorgensen et al., 2018b) in R software, we imputed the selected variables via means of multiple imputation methods and conducted principal component analysis. Finally, we extracted principal components that explained 50% of the overall variance and included these as auxiliary variables in models. It should be noted that imputations were only conducted for those variables that were used to obtain the component scores (i.e., auxiliary variables) from the principal component analysis. The missingness of our main variables (i.e., parental autonomy, intimacy, and ER) was handled with FIML estimation that utilized the created auxiliary variables to improve model estimation (Howard et al., 2015).

This procedure was conducted separately for each modeling situation to maximize the missing-at-random assumption in all four situations. The auxiliary variables (20–22 in total) were included in the developmental timing models and the parental measurement models. The only exceptions were the measurement models of ER patterns, as it was not possible to estimate factor scores from the models with auxiliary variables. Thus, the measurement models of ER patterns were tested without the auxiliary variables.

#### Criteria of Model Fit

In the comparisons of the nested models, we used the scaled chi-square difference test with α = 0.050 (Satorra and Bentler, 2001). The Akaike Information Criteria (AIC) was used in the only non-nested model comparison between the Infancy Model and the Middle Childhood Model. A difference of |AIC| ≥ 2.00 was our criterion for the meaningful difference between the models. To assess the absolute model fit, we focused on the approximate fit indices of robust comparative fit index (CFI; Brosseau-Liard and Savalei, 2014), robust root mean square error of approximation (RMSEA; Brosseau-Liard et al., 2012), and standardized root mean square residual (SRMR). The values of CFI > 0.900, RMSEA < 0.080, and SRMR < 0.080 were our criteria of adequate model fit. We decided to choose more liberal criteria than typically used cutoffs because our hypotheses considered the relative rather than absolute model fit. In the case of individual model parameters, we emphasized the effect sizes (standardized estimates and *R*^2^s) and 95% confidence intervals because our simulation study indicated much uncertainty in the interpretation of significance tests related to these parameters (for further information, see preregistration^1^).

## Results

### Preliminary Analyses

#### Descriptive Statistics

Supplementary Material 1 presents descriptive statistics for the variables used in the self-reported parental autonomy models (Supplementary Table 1A), the partner-reported parental autonomy models (Supplementary Table 1B), the self-reported parental intimacy models (Supplementary Table 1C), the partner-reported parental intimacy models (Supplementary Table 1D), and the measurement models of ER patterns (Supplementary Table 1E). It is noteworthy that all parental autonomy and intimacy items showed median values of 6 or 7, demonstrating strong skewness to the left. Yet, the range of most parental items (i.e., 73/96) varied from 1 to 7 in line with their expected maximum range. Supplementary Material 2 shows the zero-order correlations for the same variables used in the self-reported parental autonomy models (Supplementary Table 2A), the partner-reported parental autonomy models (Supplementary Table 2B), the self-reported parental intimacy models (Supplementary Table 2C), the partner-reported parental intimacy models (Supplementary Table 2D), and the measurement models of ER patterns (Supplementary Table 2E).

#### Longitudinal Measurement Models of Parental Autonomy and Intimacy

Regarding the measurement models of self- and partner-reported parental autonomy and intimacy (see Supplementary Material 3), all models showed adequate model fit when the configural time invariance structure was tested (self-reported parental autonomy: scaled χ^2^ (213, *N* = 885) = 256.48, *p* = 0.022, CFI = 0.982, RMSEA = 0.015, SRMR = 0.051; partner-reported parental autonomy: scaled χ^2^ (213, *N* = 885) = 278.45, *p* = 0.002, CFI = 0.981, RMSEA = 0.019, SRMR = 0.052; self-reported parental intimacy: scaled χ^2^ (213, *N* = 885) = 412.04, *p* < 0.001, CFI = 0.922, RMSEA = 0.033, SRMR = 0.066; and partner-reported parental intimacy: scaled χ^2^ (213, *N* = 885) = 345.97, *p* < 0.001, CFI = 0.967, RMSEA = 0.030, SRMR = 0.070). However, none of the models met any stricter assumptions of the time invariance (Supplementary Material 3), reflecting the descriptive discontinuity in the manifestation of parental autonomy and intimacy across development. Similarly, in all self- and partner-reported models, only the configural factorial invariance between the mothering and fathering held (Supplementary Material 3), suggesting that the maternal autonomy and intimacy differed from the paternal autonomy and intimacy.

Individual parameters (i.e., loadings and correlations) for the measurement models of parental autonomy and intimacy can be found in Supplementary Material 4. The standardized loadings for the models of self- and partner-reported parental autonomy ranged 0.13–0.80 and 0.20–0.83, respectively. The autonomy indicator “clinging–independent” had especially weak loadings for both mothers and fathers from T1 to T3 (self-reported: λ = 0.13–0.33; partner-reported: λ = 0.20–0.54). Nevertheless, as the overall fits of the parental autonomy models were adequate, we decided to keep this indicator in the models in line with our preregistration. The standardized loadings for the models of self- and partner-reported parental intimacy were slightly higher, ranging 0.36–0.86 and 0.32–0.92, respectively. Omega reliabilities (McDonald, 1999) for self- and partner-reported parental autonomy and self- and partner-reported parental intimacy ranged 0.54–0.63, 0.53–0.83, 0.60–0.79, and 0.62–0.89, respectively. The correlations within latent autonomy/intimacy across time (i.e., T1 and T2, T2 and T3, T1, and T3) ranged 0.42–0.47 for mothers and 0.21–0.27 for fathers in the self-reported parental autonomy model, 0.32–0.42 for mothers and 0.15–0.38 for fathers in the partner-reported parental autonomy model, 0.27–0.43 for mothers and 0.09–0.36 for fathers in the self-reported parental intimacy model, and 0.06–0.48 for mothers and 0.21–0.44 for fathers in the partner-reported parental intimacy model.

In the comparisons of the first- and second-order autoregressive cross-lagged models, the latter showed a better fit in the situations of self- and partner-reported parental autonomy (Supplementary Material 3). No difference existed between the models in the situations of self- and partner-reported parental intimacy (Supplementary Material 3). Thus, we included the second-order autoregressive structure in the developmental timing models of parental autonomy. The first-order autoregressive structure was, in turn, included in the models of parental intimacy. Regarding our sample distribution, ART status was not linked to parenting using *p* < 0.050 as a criterion apart from two exceptions (24 tested effects in total). Compared to NC families, ART families showed higher partner-reported paternal intimacy at T1 (β = 0.144, *SE* = 0.044, *p* = 0.001) and at T2 (β = 0.114, *SE* = 0.048, *p* = 0.018). Thus, in general, ART and NC families showed little differences in parenting.

#### Measurement Models of Emotion Regulation Patterns

In the measurement model of the ERQ, RMSEA did not meet the set criteria of adequate model fit, scaled χ^2^ (34, *N* = 437) = 117.56, *p* < 0.001, CFI = 0.936, RMSEA = 0.085, SRMR = 0.042. Similarly, in the case of the CERQ rumination, both the original model (χ^2^ (2, *N* = 437) = 29.66, *p* < 0.001, CFI = 0.938, RMSEA = 0.181, SRMR = 0.040) and the alternative model (scaled χ^2^ (9, *N* = 437) = 76.33, *p* < 0.001, CFI = 0.926, RMSEA = 0.142, SRMR = 0.054) showed inadequate fit in RMSEA. To solve the problem of inadequate model fit, we tested two measurement models for all three ER patterns. The first model included the original measurement models of the ERQ and CERQ rumination. The second model included the original model of the ERQ and the alternative model of the CERQ rumination. Although the differences in the model fit indices were small, only the latter model met the set criteria of model fit (scaled χ^2^ (101, *N* = 437) = 323.89, *p* < 0.001, CFI = 0.910, RMSEA = 0.076, SRMR = 0.061), whereas the former model did not (scaled χ^2^ (74, *N* = 437) = 267.08, *p* < 0.001, CFI = 0.899, RMSEA = 0.083, SRMR = 0.067). Tarkkonen’s rho for the factor scores of rumination was also higher in the alternative model of the CERQ rumination (ρ = 0.88) than in the original model (ρ = 0.78). For these reasons, we decided to use the rumination factor scores based on the alternative model of the CERQ rumination in the developmental timing models. Tarkkonen’s rhos for the factor scores of reappraisal and suppression were 0.89 and 0.80, respectively. Individual parameters for the selected measurement model of ER patterns can be found in Supplementary Material 5.

### Main Results: Comparing Developmental Timing Models

The fit indices of all four developmental timing models were adequate in all four modeling situations (self-reported parental autonomy models: CFI = 0.973–0.973, RMSEA = 0.015–0.015, SRMR = 0.050–0.051; partner-reported parental autonomy models: CFI = 0.973–0.974, RMSEA = 0.017–0.018, SRMR = 0.049–0.050; self-reported parental intimacy models: CFI = 0.924–0.925, RMSEA = 0.027–0.027, SRMR = 0.060–0.061; partner-reported parental intimacy models: CFI = 0.974–0.974, RMSEA = 0.019–0.021, SRMR = 0.065–0.066). The comparisons of developmental timing models showed consistent results across the four modeling situations: Contrary to our first hypothesis, no differences in the model fit were found between the models (Table 1). Thus, the most parsimonious Stability Model was considered as the most suitable model for the self- and partner-reported parental autonomy and self- and partner-reported parental intimacy. In other words, the parenting quality in infancy and middle childhood did not show any predictive power on adolescents’ ER patterns over and above the temporal stability of parenting.

**TABLE 1:** Comparisons of developmental timing models.

Model comparison	Δ*df*	Scaled Δχ^2^ test	*p*	ΔAIC	Δ*R*^2^ Reappraisal	Δ*R*^2^ Suppression	Δ*R*^2^ Rumination
**Self-Reported Parental Autonomy**							
Stability Model (*df* = 352) vs.							
Infancy Model	6	6.74	0.346	5.62	0.017	0.016	0.003
Middle Childhood Model	6	6.70	0.349	5.13	0.006	0.008	0.016
Whole Childhood Model	12	12.45	0.410	12.04	0.021	0.023	0.017
Infancy Model (*df* = 346) vs.							
Middle Childhood Model	0			–0.48	–0.011	–0.008	0.013
Whole Childhood Model	6	5.73	0.454	6.42	0.004	0.007	0.014
Middle Childhood Model (*df* = 346) vs.							
Whole Childhood Model	6	5.68	0.460	6.91	0.015	0.015	0.001
**Partner-Reported Parental Autonomy**							
Stability Model (*df* = 352) vs.							
Infancy Model	6	6.01	0.422	6.31	0.014	0.004	0.007
Middle Childhood Model	6	1.17	0.979	10.69	0.002	0.002	0.003
Whole Childhood Model	12	8.22	0.768	15.74	0.021	0.014	0.007
Infancy Model (*df* = 346) vs.							
Middle Childhood Model	0			4.38	–0.012	–0.002	–0.004
Whole Childhood Model	6	2.42	0.878	9.43	0.007	0.010	0.000
Middle Childhood Model (*df* = 346) vs.							
Whole Childhood Model	6	7.77	0.256	5.05	0.019	0.012	0.004
**Self-Reported Parental Intimacy**							
Stability Model (*df* = 351) vs.							
Infancy Model	6	6.87	0.333	6.54	0.004	0.012	0.010
Middle Childhood Model	6	7.80	0.253	4.27	0.017	0.022	0.004
Whole Childhood Model	12	11.49	0.487	13.65	0.017	0.026	0.011
Infancy Model (*df* = 345) vs.							
Middle Childhood Model	0			–2.26	0.013	0.010	–0.006
Whole Childhood Model	6	4.85	0.563	7.12	0.013	0.014	0.001
Middle Childhood Model (*df* = 345) vs.							
Whole Childhood Model	6	3.21	0.782	9.38	0.000	0.004	0.007
**Partner-Reported Parental Intimacy**							
Stability Model (*df* = 356) vs.							
Infancy Model	6	3.67	0.721	8.81	0.005	0.009	0.003
Middle Childhood Model	6	1.19	0.977	10.84	0.003	0.001	0.004
Whole Childhood Model	12	6.44	0.892	18.14	0.009	0.011	0.017
Infancy Model (*df* = 350) vs.							
Middle Childhood Model	0			2.03	–0.002	–0.008	0.001
Whole Childhood Model	6	2.81	0.832	9.33	0.004	0.002	0.014
Middle Childhood Model (*df* = 350) vs.							
Whole Childhood Model	6	5.50	0.481	7.30	0.006	0.010	0.013

**N* = 885. The ΔAIC and Δ*R*^2^ of reappraisal, suppression, and rumination were calculated by subtracting the estimates of the model above from the model below. In the model comparisons between the Infancy Model and Middle Childhood Model, scaled Δχ2 tests were not calculated because these models were non-nested with each other. AIC = Akaike information criterion.*

Next, in the four modeling situations, we compared the selected Stability Model and its submodel in which we also fixed the effects of parenting quality at T3 on ER patterns to zero to test whether parenting had any effects on adolescents’ ER patterns. Again, the results were consistent across the modeling situations. Contrary to our second hypothesis, there were no differences in the model fit between the Stability Models and their submodels (Table 2). Thus, no other comparisons were needed to test the role of parents’ gender (third hypothesis) and the effects of parenting quality on each specific ER pattern (fourth hypothesis).

**TABLE 2:** Comparisons of Stability Model and its submodel with no effects of parenting in late adolescence on adolescents’ emotion regulation patterns.

Model comparison	Δ*df*	Scaled	*p*	ΔAIC	Δ*R*^2^	Δ*R*^2^	Δ*R*^2^
		Δχ^2^ test			Reappraisal	Suppression	Rumination
**Self-Reported Parental Autonomy**							
Stability Model (*df* = 352) vs.							
its submodel with no effects of parenting on ER patterns (*df* = 358)	6	11.33	0.078	–1.50	–0.011	–0.001	–0.035
**Partner-Reported Parental Autonomy**							
Stability Model (*df* = 352) vs.							
its submodel with no effects of parenting on ER patterns (*df* = 358)	6	10.46	0.107	–1.04	–0.023	–0.003	–0.012
**Self-Reported Parental Intimacy**							
Stability Model (*df* = 351) vs.							
its submodel with no effects of parenting on ER patterns (*df* = 357)	6	1.03	0.985	–10.86	–0.000	–0.002	–0.001
**Partner-Reported Parental Intimacy**							
Stability Model (*df* = 356) vs.							
its submodel with no effects of parenting on ER patterns (*df* = 362)	6	11.40	0.077	0.18	–0.010	–0.006	–0.019

**N* = 885. As in Table 1, the ΔAIC and Δ*R*^2^ of reappraisal, suppression, and rumination were calculated by subtracting the estimates of the Stability Model above from its submodel below. AIC = Akaike information criterion; ER = emotion regulation.*

**FIGURE 2:**
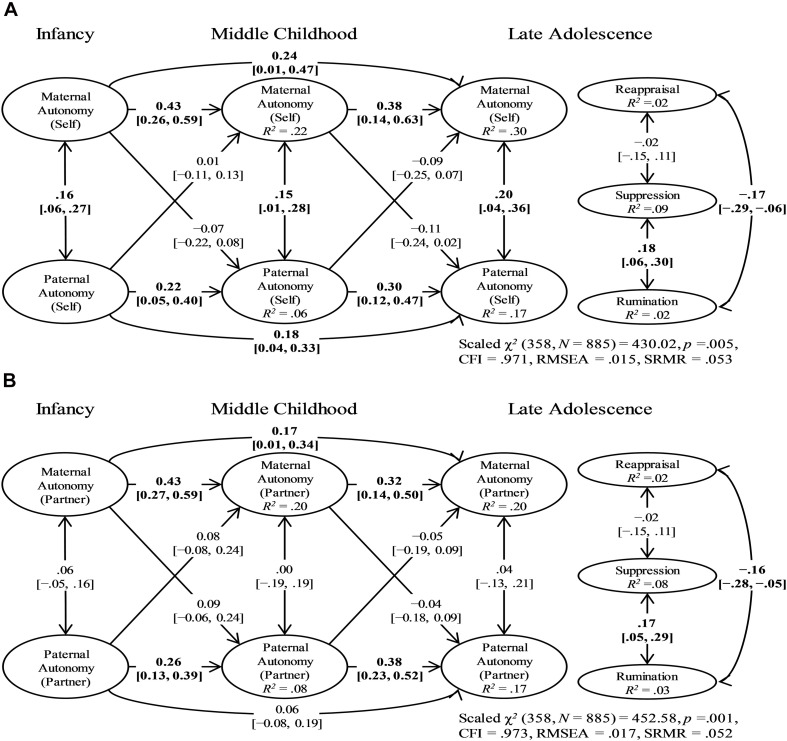
The Final Self-Reported **(A)** and Partner-Reported **(B)** Parental Autonomy Models: Standardized Parameter Estimates and 95% Confidence Intervals. There were no effects of maternal or paternal autonomy in infancy, middle childhood, and late adolescence on adolescents’ emotion regulation patterns. Bolded values represent paths in which the 95% confidence interval did not contain zero. The factor loadings and error term correlations of parental autonomy indicators and the path coefficients of covariates are not shown. CFI = robust comparative fit index; RMSEA = robust root-mean-square error of approximation; SRMR = standardized root mean square residual.

**FIGURE 3:**
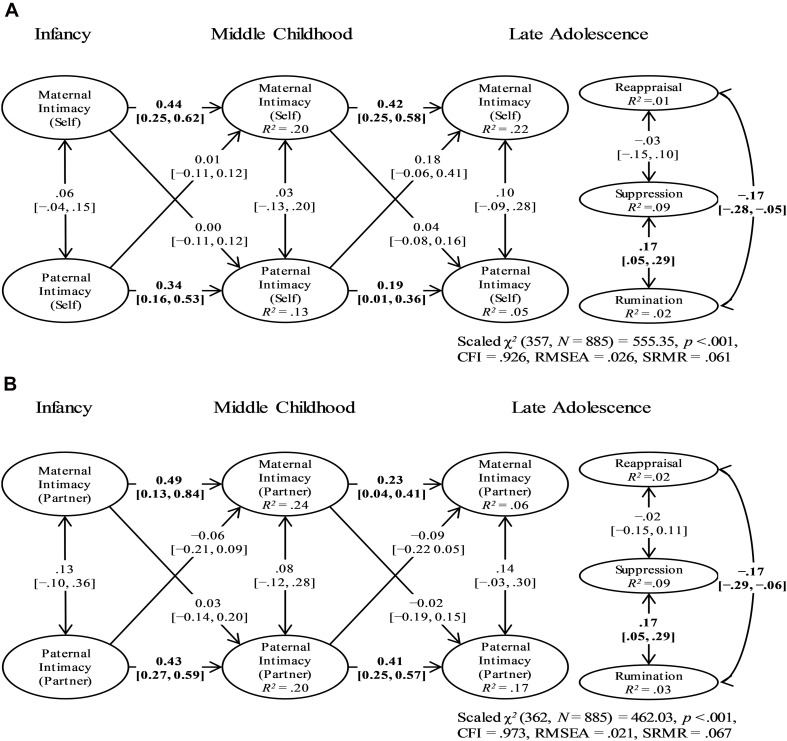
The Final Self-Reported **(A)** and Partner-Reported **(B)** Parental Intimacy Models: Standardized Parameter Estimates and 95% Confidence Intervals. There were no effects of maternal or paternal intimacy in infancy, middle childhood, and late adolescence on adolescents’ emotion regulation patterns. Bolded values represent paths in which the 95% confidence interval did not contain zero. The factor loadings and error term correlations of parental autonomy indicators and the path coefficients of covariates are not shown. CFI = robust comparative fit index; RMSEA = robust root-mean-square error of approximation; SRMR = standardized root mean square residual.

The models with no effects of parenting quality in infancy, middle childhood, and late adolescence on adolescents’ ER patterns were selected as our final model for (a) self-reported parental autonomy (Figure 2A), (b) partner-reported parental autonomy (Figure 2B), (c) self-reported parental intimacy (Figure 3A), and (d) partner-reported parental intimacy (Figure 3B). These models completely contradicted our hypotheses, suggesting that neither mothering nor fathering in any developmental stage had predictive power on adolescents’ ER patterns. Regarding other model parts, the autoregressive paths of parental autonomy/intimacy generally showed small to moderate temporal stability, whereas the cross-lagged paths between mothering and fathering were small at best (Figures 2, 3). Individual parameters for the selected models can be found in Supplementary Material 6.

### Assessing Interpretability of Null Results

In the preregistration, the first author, without access to the data, conducted a Monte Carlo simulation study to assess the power and Type I error rate of this study. However, substantial discrepancies existed between the original simulation study and the actual data in (a) the missing data structure, (b) the variable distributions, and (c) the model parameters (e.g., factor loadings) that did not directly concern our hypotheses. Therefore, to strengthen the interpretation of our results, we conducted new Monte Carlo simulations for the four modeling situations using the *simsem* package (Jorgensen et al., 2018a) in R software. In these simulations, we defined the identical missing data structure as in our actual data. For all variables, we also set kurtosis and skewness values that were comparable to our data; yet, we had to adjust several values of parental autonomy and intimacy indicators to guarantee the convergence of the Vale and Maurelli (1983) method, especially in the parental intimacy models. In the data-generating population models, we mostly used the values of individual model parameters derived from our actual conducted models as the true population parameters. The only exceptions were the effects of parental autonomy and intimacy at T1, T2, and T3 on adolescents’ ER patterns. These parameters were set to the same values as in the original simulations. Thus, depending on the theoretical assumptions of each developmental timing model, we set the standardized path coefficients of maternal and paternal autonomy/intimacy at T1 and T2 on reappraisal to 0.13 or zero, and suppression and rumination to −0.13 or zero. In all models, we set the standardized path coefficients of maternal and paternal autonomy/intimacy at T3 on reappraisal to 0.13 and suppression and rumination to −0.13. The value of ± 0.13 was based on previous studies (Gresham and Gullone, 2012; Hilt et al., 2012; Brenning et al., 2015; Cheung et al., 2019) that have focused on the role of parenting in adolescents’ ER patterns.

The results of the simulations can be found in Supplementary Material 7. The power consistently decreased compared to our original simulation study. Nevertheless, the results suggested that if the true population model had been the Whole Childhood Model, Stability Model, or Infancy Model, the power to detect the correct model in the model comparisons between the developmental timing models should have been relatively sufficient. Importantly, when the true population model was one of the four developmental timing models, the power for detecting the difference between the Stability Model and its submodel with no effects of parenting at T1, T2, and T3 on adolescents’ ER patterns ranged 0.92–1.00. These results suggest that if the true model had been one of the developmental timing models, we should have expected to find the differences between the Stability Model and the selected model with no effects of parenting on ER patterns in all four modeling situations.

### Sensitivity Analyses

The development-cohesion model of family relationships suggests that both high autonomy and high intimacy are necessary for optimal child development, whereas deficits in one or both indicate a more problematic developmental environment (Mattejat and Scholz, 1994). In our main analyses, following our preregistered protocol, we tested the separate main effects of autonomy and intimacy. This was because due to our limited sample size and model complexity, it was impractical to model the interaction between autonomy and intimacy while also taking into account (a) the stability of parenting, (b) the parenting effects in other developmental stages, and (c) the unique effects of mothers and fathers, all prerequisite to rigorously test our hypotheses. However, modeling only the separate main effects of each parental dimension may have explained our observed null results. To consider this, we inspected the robustness of our results with additional analyses in which we focused on the effects of shared variance between autonomy and intimacy at T1, T2, and T3 on adolescents’ ER patterns (separately for self- and partner reports).

In the additional analyses, we first parceled the autonomy indicators into the average variables at T1, T2, and T3 separately for mothers and fathers. The same procedure was also executed for the intimacy indicators at T1, T2, and T3. These pairs of autonomy and intimacy variables were used as the two reflective indicators of each latent parental variable at each measurement point. Thus, each latent variable of mothering and fathering at T1, T2, and T3, reflected the continuum of optimal parenting in terms of combined *high autonomy and high intimacy* versus *low autonomy and low intimacy*. The developmental timing models were tested and compared for self- and partner reports using the same approach as in the main analyses. The detailed description and results of these additional analyses can be found in Supplementary Material 8.

The results were highly similar to the main results regarding the separate main effects of parental autonomy and intimacy. The interpretation of the self-reported results was precisely the same; that is, the model with no parenting effects in infancy, middle childhood, and late adolescence on adolescents’ ER patterns was selected as the final model. Similarly, regarding the partner reports, no effects of mothering or fathering in infancy and middle childhood were detected on adolescents’ ER patterns; yet, in late adolescence, the model comparisons provided support for one effect. High autonomous and intimate fathering was linked to adolescents’ lower rumination, β = –0.182 *SE* = 0.067, *p* = 0.007. However, it should be noted that five out of six of our modeling situations did not find this effect in late adolescence, and, in total, our models considered 36 effects of mothering and fathering in late adolescence on adolescents’ ER patterns. This multiple testing increases the risk that the single detected effect can be false positive. Thus, we deemed that it was safest to emphasize the more consistent results in our interpretation. In sum, the additional analyses provided no support that our null findings in infancy and middle childhood were due to our chosen approach of modeling the effects of autonomy and intimacy separately. In late adolescence, the analyses yielded only one effect, also showing marginal differences to our main analyses.

## Discussion

Current attachment, neurodevelopmental, and evolutionary–developmental models suggest that early experiences within the parent–child relationships during infancy and middle childhood may have a long-lasting impact on later ER development (Del Giudice et al., 2011; Gee, 2016; Perry et al., 2017; Szepsenwol and Simpson, 2019). Empirical work has further implied that parenting quality may still shape children’s ER development in adolescence (Morris et al., 2017). However, our preregistered study was the first to rigorously test the stage-specific effects of parenting quality by taking into account both the temporal stability of parenting and its relative contributions in other developmental stages. We formulated four developmental timing models to test the stage-specific legacy of mothering and fathering in infancy, middle childhood, and late adolescence on adolescents’ ER patterns. Yet, entirely against our hypotheses, none of the developmental timing models were supported. We found no effects of mothering or fathering in infancy, middle childhood, or late adolescence on adolescents’ ER patterns. The results were consistent irrespective of both the reporter (i.e., self or partner) and the parental dimension (i.e., autonomy or intimacy). Importantly, the Monte Carlo simulations indicated that our study had sufficient power to detect the differences between the developmental timing models and the final selected models with no effects of parenting on adolescents’ ER patterns. Thus, our findings provide no support for the conceptual idea of sensitive periods in infancy and middle childhood, during which parenting quality has long-term effects on children’s ER development. The findings also deviate from the previously documented link between parenting quality in adolescence and adolescents’ ER patterns.

### No Long-Term Effects of Parenting in Infancy and Middle Childhood

Several tentative explanations exist for why we did not find long-term effects of mothering or fathering on adolescents’ ER patterns. One possibility may be that although parenting can be a key factor in shaping children’s ER patterns within infancy and middle childhood, these effects may later disappear due to the high developmental plasticity of ER patterns through development. From the evolutionary–developmental standpoint, rather than viewing early attachment-related ER patterns as long-term adaptations preparing children for *future ecological contexts*, these ER patterns might be better understood in terms of stage-specific ontogenetic adaptations, mainly helping children to function *only within the current proximal environment* (Bjorklund, 2015). Thus, early ER patterns, especially in infancy, might develop to foster coping with stage-specific challenges (e.g., maintain proximity to parents), but can be highly malleable during later development.

In addition to our main findings, the idea of the ontogenetic adaptations is consistent with our finding that the stability of parenting was only small to moderate. This small-to-moderate stability suggests that the parenting quality in infancy and middle childhood may not be such a reliable cue about future environments for ER systems to organize long-term development. Viewing early ER patterns as ontogenetic adaptations is also compatible with research that has stressed the buffering and protective role of the parents in children’s ER within infancy (Hostinar et al., 2014) and middle childhood (Gee et al., 2014; Hostinar et al., 2015). However, to make any more definite conclusions about the potential ontogenetic adaptations of early ER patterns, long-term studies that measure both parenting and children’s ER patterns in infancy and middle childhood are necessary. It is also notable that we did not find support for this ontogenetic adaptation explanation in late adolescence as parenting quality in late adolescence showed no effects on adolescents’ ER patterns. Nevertheless, this does not exclude the possibility that ER patterns within the parent–child relationships in infancy and middle childhood could function as ontogenetic adaptations. During these earlier developmental stages, the importance of the parent–child relationships as children’s proximal environment is considerably higher than in late adolescence.

Alternatively, children may substantially vary in how strongly parenting in infancy and middle childhood shapes their long-term ER development, for better and for worse. This core prediction of differential susceptibility theories (Ellis et al., 2011) emphasizes the importance of taking into account children’s *neurobiological susceptibility* to environmental input, marked by temperamental (e.g., negative emotionality) and other (endo)phenotypic characteristics, when studying the role of parents in children’s ER development. The lack of long-term parenting effects in our study may indicate that some children’s ER development is susceptible to both high and low parenting quality, whereas others are highly resistant to experiences within the parent–child relationships. It is noteworthy that the previous long-term study by Hilt et al. (2012) showed that children’s high negative emotionality might strengthen the effects of maternal intrusiveness at preschool age on adolescents’ greater rumination, while also showing the main effects of intrusiveness on rumination. Our study could not examine these potential moderation effects because our data did not include proper susceptibility markers in infancy. Thus, future studies are needed to consider the role of neurobiological susceptibility when testing the stage-specific effects of parenting on children’s long-term ER development.

Finally, children’s widening developmental environments may explain the lack of long-term parenting effects on adolescents’ ER patterns in our study, especially in middle childhood. In fact, key developmental tasks in middle childhood are highly related to peer relations, including social learning and integration (i.e., adopting norms and activities) as well as social competition (i.e., achieving status and friendships; Del Giudice, 2018). How well children cope with these tasks may shape their developmental trajectory of ER. Therefore, if we had focused on peer relations in middle childhood, we might have observed stage-specific effects on adolescents’ ER patterns. Yet, while being exposed to peer victimization has been linked to less constructive ER patterns in middle childhood (Gardner et al., 2017), such long-term studies spanning to adolescence are lacking. Thus, research is needed to examine the impact of peer relations in middle childhood on adolescents’ ER patterns.

### Lack of Concurrent Parenting Effects on Emotion Regulation in Adolescence

Unexpectedly, we also found no stage-specific effects of parenting in late adolescence on adolescents’ ER patterns. This finding differs from previous studies (Ruijten et al., 2011; Gresham and Gullone, 2012; Brenning et al., 2015; Cheung et al., 2019) that have shown parenting quality in adolescence to associate with adolescents’ ER patterns. These differences might, in part, be explained by the fact that while our study focused on late adolescence, most previous studies have examined parenting and adolescents’ ER patterns in early or middle adolescence (Ruijten et al., 2011; Gresham and Gullone, 2012; Gaté et al., 2013; Brenning et al., 2015). The role of parents in children’s ER might be more salient in earlier compared to late adolescence that involves higher independence from parents. However, some research has still observed the associations of parenting in late adolescence with adolescents’ ER patterns (Cheung et al., 2019), implying that the timing of assessment in adolescence does not solely explain the differences between our study and previous ones.

Another difference between our study and previous ones is that whereas most previous studies used adolescents’ reports to assess both parenting quality and ER (Ruijten et al., 2011; Gresham and Gullone, 2012; Brenning et al., 2015; Cheung et al., 2019), we used parental reports to assess parenting quality and adolescents’ reports to assess their ER. From this point of view, at least two plausible explanations exist for the different results between our study and the previous studies. On the one hand, adolescent-reported parenting may show stronger effects on adolescents’ ER compared to parental reports because adolescents’ reports can capture experiences in the parent–child relationships that are outside of parents’ perceptions. In line with this, in a recent study, only adolescent-reported but not parent-reported parenting in midadolescence predicted changes in adolescents’ ER, indicating that adolescents’ reports of parenting may have higher predictive validity on adolescents’ ER compared to parental reports (Van Lissa et al., 2019). If so, future research should emphasize adolescents’ reports when assessing parenting quality.

On the other hand, however, common method bias due to the same evaluator may also explain some of the previous research findings. Although self-reports on parenting have limited validity due to reporting biases, such as socially desirable reporting (Morsbach and Prinz, 2006), it should be noted that we also used partner reports of parenting. Partner-reported parenting seems to be less prone to these biases and, thus, increase the validity of the measurement (Bögels and van Melick, 2004). Our results were consistent regardless of whether parental self- or partner reports were used, which provides tentative support for the common method bias explanation. If so, parenting in late adolescence would not be such a critical factor for adolescents’ ER patterns. This would suggest that future ER research should focus on other, more age-salient intimate relationships with partners and friends. At any rate, different sources of measurements enabled us to test the link between parenting quality in adolescence and adolescents’ ER patterns more rigorously. However, more research is still required about the role of the evaluator in the association between parenting in adolescence and adolescents’ ER patterns.

### Potential Relationship Specificity of Emotional Regulation Patterns

On a general level, the weak contributions of parenting quality in all developmental stages on adolescents’ ER patterns may also be explained by the high *relationship-specificity* of ER patterns that develop within the mother–child and father–child relationships. In line with the group socialization theory (Harris, 1995), children may develop a specific pattern to regulate emotions within the family but show distinct ER patterns outside of the family due to different demands in these developmental environments. Attachment research has provided some indirect support for this hypothesis, showing only modest associations between late adolescents’ parent-specific and general attachment patterns (Klohnen et al., 2005). Relatedly, it is also possible that ER patterns developed within the parent–child relationships may generalize only to intimate relationships, as was suggested in a recent study on early attachment and ER patterns in adulthood (Girme et al., 2020). Future research should test whether parenting quality contributes to ER patterns only within the parent–child relationships and/or other intimate relationships but not in other life contexts.

### Strengths and Limitations

The strengths of our study include the long-term design that allowed us to examine novel research questions on ER development. We also preregistered the study plan before analyzing the data and followed this plan with two justified exceptions. In light of the recent years’ discussion about the lack of transparency and the high number of researchers’ degrees of freedom in psychological research, preregistration can decrease the risk for false positives (Nelson et al., 2018). At least, it gives researchers a better opportunity to evaluate the credibility of findings. Notably, the benefits of preregistration can even be accentuated when testing complex developmental hypotheses with unique longitudinal data.

Our study also has several limitations. First, although ART status seemed to have a minor role in our analyses, the selectiveness of our sample (half of the families received ART) limits the generalizability of our findings. Second, the attachment and evolutionary–developmental theories emphasize, for example, predictability and harshness as the most critical parental dimensions for child development. Thus, parental autonomy and intimacy may not have sufficiently captured the most relevant aspects of parenting quality, especially during the early stages. Third, despite our multi-informant assessments of parenting, we used only questionnaire-based information reported by parents. Parental reports are prone to several reporting biases (Morsbach and Prinz, 2006), albeit some of these biases are mitigated in partner reports (Bögels and van Melick, 2004). The use of observational methods might have led to different results. Indeed, meta-analytical work shows that the correlations between parental self-reports and observational measures tend to be small (Hendriks et al., 2018), implying that these measures mostly capture different aspects of parenting. Meanwhile, however, when comparing the predictive validity of parental reports and observational measures of parenting on children’s internalizing symptoms (indicative of children’s ER problems), meta-analyses show hardly any differences (Pinquart, 2017) or inconsistent results (McLeod et al., 2007a,b). Thus, to date, there is no clear empirical evidence that observational measures would have shown stronger effects on adolescents’ ER patterns. The limitations regarding self-reports are also true for adolescents’ ER patterns. Fourth, our measurement of parental autonomy and intimacy showed only configural time invariance, a limitation in reliably testing sensitive periods. Nevertheless, it is also quite expectable that the manifestation of parental autonomy and intimacy in one developmental stage (e.g., infancy) is not the same as in another developmental stage (e.g., late adolescence) due to the unique stage-specific challenges in parenting. Interestingly, parental autonomy and intimacy still showed small-to-moderate stability. Thus, we deem it possible that our results reflect heterogeneous continuity in parental autonomy and intimacy, involving some variance in their manifestation but continuity in the underlying latent constructs (Petersen et al., 2020). Fifth, we could not include preschool age in our data collection as a potential sensitive period for parenting effects, although parenting seems to play a crucial role in children’s ER during this developmental stage (Hilt et al., 2012; Gee, 2016). Sixth, most families in our sample showed high levels of parental autonomy and intimacy; that is, these variables were skewed toward high parenting quality. Our relatively well-adjusted sample may have been limited to demonstrate the effects of parenting on adolescents’ ER patterns and, thus, provide evidence for sensitive periods. Finally, attrition was high in our data. Although we used rigorous statistical approaches to handle missing data and our Monte Carlo simulations indicated relatively sufficient power, the high attrition increases the uncertainty of our findings. While missingness was highest in father reports, the inclusion of fathers was, nevertheless, our one contribution to the field that has typically ignored the role of fathers in children’s ER development (Morris et al., 2017).

## Conclusion

Current developmental models emphasize the role of parenting in children’s ER development. Our preregistered study was the first to test the relative importance of parenting quality in infancy, middle childhood, and late adolescence on adolescents’ ER patterns. Surprisingly, we found no effects of maternal or paternal autonomy or intimacy in any developmental stage on adolescents’ ER patterns. Our findings provide no support for the stage-specific effects of parenting in infancy, middle childhood, or late adolescence on adolescents’ emotion regulation. Instead, our findings might reflect the ongoing developmental plasticity of ER from infancy to late adolescence. We hope that our study will stimulate further research that focuses on other parental dimensions, children’s neurobiological susceptibility, and, in addition to the parent–child relationships, other age-salient relationships when trying to recognize key environmental factors shaping children’s long-term ER development.

## Data Availability Statement

The data analyzed in this study is subject to the following licenses/restrictions: The participants provided their informed consent to allow only certain researchers to use the data. This was based on The Ethical Board of Helsinki University Hospital’s requirements of the data usage. Requests concerning data analyses should be directed to JT, jaakko.tammilehto@gmail.com, https://projects.tuni.fi/kehi/.

## Ethics Statement

The studies involving human participants were reviewed and approved by The Ethical Board of Helsinki University Central Hospital. Written informed consent to participate in this study was provided by the participants’ legal guardian/next of kin.

## Author Contributions

JT, with the support of JLn and R-LP, conceived of the presented research idea and hypotheses. JT formulated the initial drafts of the manuscript and preregistration and revised them in collaboration with JLn, R-LP, MF, MV, LH, JLp, PP, and AT. R-LP, JLn, MF, MV, LH, PP, and AT contributed to collecting the empirical data. JT conducted all data preparations and statistical analyses. JLp provided expertise in the statistical analyses. All the authors have made a significant contribution to this study, read and approved the final manuscript.

## Conflict of Interest

The authors declare that the research was conducted in the absence of any commercial or financial relationships that could be construed as a potential conflict of interest.

## Footnotes

1 https://osf.io/nehmf
