# Supplementary material for: Developmental Stage-Specific Effects of Parenting on Adolescents’ Emotion Regulation: A Longitudinal Study From Infancy to Late Adolescence
Source: Front Psychol. 2021 Jun 4;12:582770. doi: 10.3389/fpsyg.2021.582770 (PMC8211896; doi:10.3389/fpsyg.2021.582770)
Supplement: Supplementary file 1 [file Table_1.docx]

Supplementary Material 1: **Descriptive Statistics**

Supplementary **Table 1A.** Descriptive Statistics of Variables in Self-Reported Parental Autonomy Models.

|  | | | | | | | | | | | | | | | | |
| --- | --- | --- | --- | --- | --- | --- | --- | --- | --- | --- | --- | --- | --- | --- | --- | --- |
| Variable | *n* | *M* | *SD* | | *Mdn* | | | min | | max | | | skewness | | kurtosis | |
| Maternal autonomy 1 at T1 | 543 | 6.00 | 1.35 | | 6.00 | | | 1.00 | | 7.00 | | | −1.82 | | 2.93 | |
| Maternal autonomy 2 at T1 | 542 | 6.41 | 0.69 | | 7.00 | | | 4.00 | | 7.00 | | | −1.04 | | 0.96 | |
| Maternal autonomy 3 at T1 | 542 | 6.41 | 0.84 | | 7.00 | | | 2.00 | | 7.00 | | | −2.43 | | 8.72 | |
| Maternal autonomy 4 at T1 | 543 | 6.41 | 0.79 | | 7.00 | | | 1.00 | | 7.00 | | | −2.22 | | 8.78 | |
| Maternal autonomy 1 at T2 | 506 | 5.75 | 1.51 | | 6.00 | | | 1.00 | | 7.00 | | | −1.53 | | 1.60 | |
| Maternal autonomy 2 at T2 | 516 | 6.37 | 0.76 | | 6.00 | | | 2.00 | | 7.00 | | | −1.70 | | 5.41 | |
| Maternal autonomy 3 at T2 | 516 | 6.34 | 0.90 | | 6.00 | | | 1.00 | | 7.00 | | | −2.38 | | 8.34 | |
| Maternal autonomy 4 at T2 | 515 | 6.31 | 0.90 | | 6.00 | | | 1.00 | | 7.00 | | | −2.22 | | 7.88 | |
| Maternal autonomy 1 at T3 | 448 | 5.67 | 1.38 | | 6.00 | | | 1.00 | | 7.00 | | | −1.19 | | 0.70 | |
| Maternal autonomy 2 at T3 | 449 | 6.09 | 1.11 | | 6.00 | | | 1.00 | | 7.00 | | | −2.09 | | 5.87 | |
| Maternal autonomy 3 at T3 | 449 | 5.95 | 1.08 | | 6.00 | | | 1.00 | | 7.00 | | | −1.83 | | 4.67 | |
| Maternal autonomy 4 at T3 | 449 | 6.10 | 1.06 | | 6.00 | | | 2.00 | | 7.00 | | | −1.57 | | 2.63 | |
| Paternal autonomy 1 at T1 | 500 | 5.56 | 1.61 | | 6.00 | | | 1.00 | | 7.00 | | | −1.38 | | 1.10 | |
| Paternal autonomy 2 at T1 | 501 | 6.30 | 0.77 | | 6.00 | | | 3.00 | | 7.00 | | | −1.03 | | 0.98 | |
| Paternal autonomy 3 at T1 | 502 | 6.32 | 0.86 | | 6.00 | | | 2.00 | | 7.00 | | | −1.89 | | 5.65 | |
| Paternal autonomy 4 at T1 | 502 | 6.27 | 0.95 | | 6.00 | | | 1.00 | | 7.00 | | | −2.00 | | 5.80 | |
| Paternal autonomy 1 at T2 | 294 | 5.49 | 1.50 | | 6.00 | | | 1.00 | | 7.00 | | | −1.22 | | 0.74 | |
| Paternal autonomy 2 at T2 | 292 | 6.23 | 0.85 | | 6.00 | | | 1.00 | | 7.00 | | | −2.10 | | 8.29 | |
| Paternal autonomy 3 at T2 | 294 | 6.24 | 0.88 | | 6.00 | | | 2.00 | | 7.00 | | | −1.59 | | 3.51 | |
| Paternal autonomy 4 at T2 | 294 | 6.21 | 0.87 | | 6.00 | | | 1.00 | | 7.00 | | | −1.69 | | 5.81 | |
| Paternal autonomy 1 at T3 | 357 | 5.22 | 1.60 | | 6.00 | | | 1.00 | | 7.00 | | | −0.85 | | −0.19 | |
| Paternal autonomy 2 at T3 | 357 | 5.88 | 1.13 | | 6.00 | | | 1.00 | | 7.00 | | | −1.56 | | 3.17 | |
| Paternal autonomy 3 at T3 | 357 | 5.62 | 1.17 | | 6.00 | | | 1.00 | | 7.00 | | | −1.21 | | 1.61 | |
| Paternal autonomy 4 at T3 | 357 | 5.84 | 1.09 | | 6.00 | | | 2.00 | | 7.00 | | | −1.27 | | 1.73 | |
| Parents' average education level at T1 | 806 | 2.87 | 0.86 | | 3.00 | | | 1.00 | | 4.00 | | | −0.36 | | −0.78 | |
| Assisted reproduction treatment | 885 | 0.50 | 0.50 | | 1.00 | | | 0.00 | | 1.00 | | | 0.00 | | −2.00 | |
| Child's sex | 806 | 0.50 | 0.50 | | 1.00 | | | 0.00 | | 1.00 | | | −0.02 | | −2.00 | |
| Reappraisal | 437 | 0.00 | 1.06 | | 0.06 | | | −2.92 | | 2.30 | | | −0.31 | | −0.03 | |
| Suppression | 437 | 0.00 | 1.12 | | 0.00 | | | −2.10 | | 2.78 | | | 0.19 | | −0.62 | |
| Rumination | 437 | 0.00 | 1.06 | | −0.17 | | | −1.51 | | 3.84 | | | 0.87 | | 0.60 | |
| *Note.* T1 = Infancy; T2 = Middle childhood; T3 = Late adolescence. | | | |  | |  |  | |  | |  |  | |  | |  |
|  | | | |  | |  |  | |  | |  |  | |  | |  |

Supplementary **Table 1B.** Descriptive Statistics of Variables in Partner-Reported Parental Autonomy Models.

|  | | | | | | | | | | | | | | | |
| --- | --- | --- | --- | --- | --- | --- | --- | --- | --- | --- | --- | --- | --- | --- | --- |
| Variable | *n* | *M* | *SD* | | *Mdn* | | | min | | max | | skewness | | kurtosis | |
| Maternal autonomy 1 at T1 | 498 | 5.68 | 1.77 | | 6.00 | | | 1.00 | | 7.00 | | −1.52 | | 1.21 | |
| Maternal autonomy 2 at T1 | 499 | 6.40 | 0.83 | | 7.00 | | | 1.00 | | 7.00 | | −2.42 | | 10.55 | |
| Maternal autonomy 3 at T1 | 499 | 6.40 | 0.90 | | 7.00 | | | 1.00 | | 7.00 | | −2.35 | | 7.51 | |
| Maternal autonomy 4 at T1 | 499 | 6.41 | 0.87 | | 7.00 | | | 1.00 | | 7.00 | | −2.57 | | 9.97 | |
| Maternal autonomy 1 at T2 | 293 | 5.51 | 1.62 | | 6.00 | | | 1.00 | | 7.00 | | −1.27 | | 0.69 | |
| Maternal autonomy 2 at T2 | 290 | 6.28 | 0.84 | | 6.00 | | | 2.00 | | 7.00 | | −1.62 | | 4.45 | |
| Maternal autonomy 3 at T2 | 290 | 6.21 | 1.07 | | 6.00 | | | 1.00 | | 7.00 | | −2.08 | | 5.27 | |
| Maternal autonomy 4 at T2 | 292 | 6.19 | 0.98 | | 6.00 | | | 1.00 | | 7.00 | | −2.03 | | 6.21 | |
| Maternal autonomy 1 at T3 | 353 | 5.25 | 1.63 | | 6.00 | | | 1.00 | | 7.00 | | −0.91 | | −0.12 | |
| Maternal autonomy 2 at T3 | 353 | 5.80 | 1.08 | | 6.00 | | | 1.00 | | 7.00 | | −0.96 | | 1.05 | |
| Maternal autonomy 3 at T3 | 353 | 5.59 | 1.40 | | 6.00 | | | 1.00 | | 7.00 | | −1.21 | | 0.96 | |
| Maternal autonomy 4 at T3 | 353 | 5.58 | 1.32 | | 6.00 | | | 1.00 | | 7.00 | | −1.06 | | 0.77 | |
| Paternal autonomy 1 at T1 | 539 | 5.79 | 1.35 | | 6.00 | | | 1.00 | | 7.00 | | −1.39 | | 1.56 | |
| Paternal autonomy 2 at T1 | 540 | 5.95 | 1.10 | | 6.00 | | | 1.00 | | 7.00 | | −1.53 | | 2.86 | |
| Paternal autonomy 3 at T1 | 541 | 6.08 | 1.12 | | 6.00 | | | 1.00 | | 7.00 | | −1.64 | | 2.86 | |
| Paternal autonomy 4 at T1 | 541 | 5.94 | 1.18 | | 6.00 | | | 1.00 | | 7.00 | | −1.67 | | 3.30 | |
| Paternal autonomy 1 at T2 | 489 | 5.87 | 1.37 | | 6.00 | | | 1.00 | | 7.00 | | −1.71 | | 2.72 | |
| Paternal autonomy 2 at T2 | 500 | 6.25 | 0.99 | | 6.00 | | | 1.00 | | 7.00 | | −2.08 | | 5.94 | |
| Paternal autonomy 3 at T2 | 496 | 6.05 | 1.28 | | 6.00 | | | 1.00 | | 7.00 | | −1.77 | | 3.03 | |
| Paternal autonomy 4 at T2 | 498 | 6.05 | 1.18 | | 6.00 | | | 1.00 | | 7.00 | | −1.67 | | 2.83 | |
| Paternal autonomy 1 at T3 | 438 | 5.79 | 1.47 | | 6.00 | | | 1.00 | | 7.00 | | −1.40 | | 1.35 | |
| Paternal autonomy 2 at T3 | 438 | 5.89 | 1.26 | | 6.00 | | | 1.00 | | 7.00 | | −1.22 | | 0.91 | |
| Paternal autonomy 3 at T3 | 438 | 5.65 | 1.48 | | 6.00 | | | 1.00 | | 7.00 | | −1.21 | | 0.87 | |
| Paternal autonomy 4 at T3 | 438 | 5.61 | 1.54 | | 6.00 | | | 1.00 | | 7.00 | | −1.10 | | 0.33 | |
| Parents' average education level at T1 | 806 | 2.87 | 0.86 | | 3.00 | | | 1.00 | | 4.00 | | −0.36 | | −0.78 | |
| Assisted reproduction treatment | 885 | 0.50 | 0.50 | | 1.00 | | | 0.00 | | 1.00 | | 0.00 | | −2.00 | |
| Child's sex | 806 | 0.50 | 0.50 | | 1.00 | | | 0.00 | | 1.00 | | −0.02 | | −2.00 | |
| Reappraisal | 437 | 0.00 | 1.06 | | 0.06 | | | −2.92 | | 2.30 | | −0.31 | | −0.03 | |
| Suppression | 437 | 0.00 | 1.12 | | 0.00 | | | −2.10 | | 2.78 | | 0.19 | | −0.62 | |
| Rumination | 437 | 0.00 | 1.06 | | −0.17 | | | −1.51 | | 3.84 | | 0.87 | | 0.60 | |
| *Note.* T1 = Infancy; T2 = Middle childhood; T3 = Late adolescence. | | | |  | |  |  | |  | |  |  |  | |  |
|  | | | |  | |  |  | |  | |  |  |  | |  |

Supplementary **Table 1C.** Descriptive Statistics of Variables in Self-Reported Parental Intimacy Models.

|  | | |  | |  | | |  | | | |  | |  | | |  | |  | | |  | |
| --- | --- | --- | --- | --- | --- | --- | --- | --- | --- | --- | --- | --- | --- | --- | --- | --- | --- | --- | --- | --- | --- | --- | --- |
| Variable | *n* | *M* | | *SD* | | | *Mdn* | | | | min | | | | max | | | skewness | | | kurtosis | | |
| Maternal intimacy 1 at T1 | 542 | 6.89 | | 0.36 | | | 7.00 | | | | 3.00 | | | | 7.00 | | | −4.56 | | | 30.91 | | |
| Maternal intimacy 2 at T1 | 542 | 6.85 | | 0.43 | | | 7.00 | | | | 3.00 | | | | 7.00 | | | −4.08 | | | 24.63 | | |
| Maternal intimacy 3 at T1 | 543 | 6.54 | | 0.69 | | | 7.00 | | | | 2.00 | | | | 7.00 | | | −1.87 | | | 5.54 | | |
| Maternal intimacy 4 at T1 | 542 | 6.92 | | 0.38 | | | 7.00 | | | | 1.00 | | | | 7.00 | | | −8.98 | | | 117.04 | | |
| Maternal intimacy 1 at T2 | 518 | 6.82 | | 0.44 | | | 7.00 | | | | 3.00 | | | | 7.00 | | | −2.93 | | | 13.01 | | |
| Maternal intimacy 2 at T2 | 516 | 6.65 | | 0.69 | | | 7.00 | | | | 1.00 | | | | 7.00 | | | −3.38 | | | 19.05 | | |
| Maternal intimacy 3 at T2 | 517 | 6.34 | | 0.81 | | | 6.00 | | | | 1.00 | | | | 7.00 | | | −2.14 | | | 9.19 | | |
| Maternal intimacy 4 at T2 | 516 | 6.81 | | 0.52 | | | 7.00 | | | | 1.00 | | | | 7.00 | | | −4.67 | | | 35.90 | | |
| Maternal intimacy 1 at T3 | 449 | 6.65 | | 0.81 | | | 7.00 | | | | 1.00 | | | | 7.00 | | | −4.19 | | | 23.04 | | |
| Maternal intimacy 2 at T3 | 449 | 6.37 | | 1.07 | | | 7.00 | | | | 1.00 | | | | 7.00 | | | −2.68 | | | 8.53 | | |
| Maternal intimacy 3 at T3 | 449 | 6.25 | | 0.98 | | | 6.00 | | | | 1.00 | | | | 7.00 | | | −2.14 | | | 6.32 | | |
| Maternal intimacy 4 at T3 | 449 | 6.66 | | 0.73 | | | 7.00 | | | | 1.00 | | | | 7.00 | | | −3.26 | | | 14.89 | | |
| Paternal intimacy 1 at T1 | 502 | 6.72 | | 0.54 | | | 7.00 | | | | 3.00 | | | | 7.00 | | | −2.58 | | | 10.32 | | |
| Paternal intimacy 2 at T1 | 502 | 6.69 | | 0.72 | | | 7.00 | | | | 1.00 | | | | 7.00 | | | −4.30 | | | 26.62 | | |
| Paternal intimacy 3 at T1 | 502 | 6.40 | | 0.79 | | | 7.00 | | | | 1.00 | | | | 7.00 | | | −2.01 | | | 7.13 | | |
| Paternal intimacy 4 at T1 | 502 | 6.83 | | 0.44 | | | 7.00 | | | | 4.00 | | | | 7.00 | | | −2.85 | | | 9.37 | | |
| Paternal intimacy 1 at T2 | 295 | 6.54 | | 0.71 | | | 7.00 | | | | 2.00 | | | | 7.00 | | | −2.37 | | | 9.97 | | |
| Paternal intimacy 2 at T2 | 294 | 6.40 | | 0.84 | | | 7.00 | | | | 1.00 | | | | 7.00 | | | −2.40 | | | 9.48 | | |
| Paternal intimacy 3 at T2 | 294 | 6.09 | | 0.91 | | | 6.00 | | | | 1.00 | | | | 7.00 | | | −1.36 | | | 3.40 | | |
| Paternal intimacy 4 at T2 | 295 | 6.62 | | 0.74 | | | 7.00 | | | | 2.00 | | | | 7.00 | | | −3.20 | | | 14.56 | | |
| Paternal intimacy 1 at T3 | 357 | 6.24 | | 0.94 | | | 6.00 | | | | 1.00 | | | | 7.00 | | | −2.05 | | | 6.75 | | |
| Paternal intimacy 2 at T3 | 357 | 5.78 | | 1.27 | | | 6.00 | | | | 1.00 | | | | 7.00 | | | −1.32 | | | 1.49 | | |
| Paternal intimacy 3 at T3 | 357 | 5.94 | | 1.08 | | | 6.00 | | | | 2.00 | | | | 7.00 | | | −1.49 | | | 2.55 | | |
| Paternal intimacy 4 at T3 | 357 | 6.15 | | 1.05 | | | 6.00 | | | | 1.00 | | | | 7.00 | | | −1.60 | | | 3.22 | | |
| Parents' average education level at T1 | 806 | 2.87 | | 0.86 | | | 3.00 | | | | 1.00 | | | | 4.00 | | | −0.36 | | | −0.78 | | |
| Assisted reproduction treatment | 885 | 0.50 | | 0.50 | | | 1.00 | | | | 0.00 | | | | 1.00 | | | 0.00 | | | −2.00 | | |
| Child's sex | 806 | 0.50 | | 0.50 | | | 1.00 | | | | 0.00 | | | | 1.00 | | | −0.02 | | | −2.00 | | |
| Reappraisal | 437 | 0.00 | | 1.06 | | | 0.06 | | | | −2.92 | | | | 2.30 | | | −0.31 | | | −0.03 | | |
| Suppression | 437 | 0.00 | | 1.12 | | | 0.00 | | | | −2.10 | | | | 2.78 | | | 0.19 | | | −0.62 | | |
| Rumination | 437 | 0.00 | | 1.06 | | | −0.17 | | | | −1.51 | | | | 3.84 | | | 0.87 | | | 0.60 | | |
| *Note.* T1 = Infancy; T2 = Middle childhood; T3 = Late adolescence. | | | | | |  | | |  |  | | |  | | |  | |  | |  | | |  |
|  | | | | | |  | | |  |  | | |  | | |  | |  | |  | | |  |

Supplementary **Table 1D.** Descriptive Statistics of Variables in Partner-Reported Parental Intimacy Models.

|  | | |  | |  | | |  | | | |  | |  | | |  | |  | | |  | |
| --- | --- | --- | --- | --- | --- | --- | --- | --- | --- | --- | --- | --- | --- | --- | --- | --- | --- | --- | --- | --- | --- | --- | --- |
| Variable | *n* | *M* | | *SD* | | | *Mdn* | | | | min | | | | max | | | skewness | | | kurtosis | | |
| Maternal intimacy 1 at T1 | 500 | 6.84 | | 0.53 | | | 7.00 | | | | 1.00 | | | | 7.00 | | | −5.82 | | | 47.90 | | |
| Maternal intimacy 2 at T1 | 500 | 6.77 | | 0.73 | | | 7.00 | | | | 1.00 | | | | 7.00 | | | −5.20 | | | 32.93 | | |
| Maternal intimacy 3 at T1 | 500 | 6.54 | | 0.77 | | | 7.00 | | | | 2.00 | | | | 7.00 | | | −2.23 | | | 6.89 | | |
| Maternal intimacy 4 at T1 | 500 | 6.90 | | 0.42 | | | 7.00 | | | | 1.00 | | | | 7.00 | | | −7.71 | | | 86.08 | | |
| Maternal intimacy 1 at T2 | 291 | 6.66 | | 0.75 | | | 7.00 | | | | 2.00 | | | | 7.00 | | | −3.34 | | | 14.27 | | |
| Maternal intimacy 2 at T2 | 291 | 6.51 | | 0.82 | | | 7.00 | | | | 2.00 | | | | 7.00 | | | −2.38 | | | 7.56 | | |
| Maternal intimacy 3 at T2 | 291 | 6.20 | | 1.08 | | | 7.00 | | | | 1.00 | | | | 7.00 | | | −1.72 | | | 3.31 | | |
| Maternal intimacy 4 at T2 | 291 | 6.72 | | 0.74 | | | 7.00 | | | | 1.00 | | | | 7.00 | | | −4.31 | | | 23.77 | | |
| Maternal intimacy 1 at T3 | 353 | 6.24 | | 1.15 | | | 7.00 | | | | 1.00 | | | | 7.00 | | | −2.12 | | | 5.26 | | |
| Maternal intimacy 2 at T3 | 353 | 5.98 | | 1.26 | | | 6.00 | | | | 1.00 | | | | 7.00 | | | −1.59 | | | 2.39 | | |
| Maternal intimacy 3 at T3 | 353 | 5.75 | | 1.34 | | | 6.00 | | | | 1.00 | | | | 7.00 | | | −1.29 | | | 1.32 | | |
| Maternal intimacy 4 at T3 | 353 | 6.23 | | 1.15 | | | 7.00 | | | | 1.00 | | | | 7.00 | | | −1.86 | | | 3.71 | | |
| Paternal intimacy 1 at T1 | 541 | 6.65 | | 0.75 | | | 7.00 | | | | 2.00 | | | | 7.00 | | | −3.02 | | | 11.56 | | |
| Paternal intimacy 2 at T1 | 538 | 6.74 | | 0.70 | | | 7.00 | | | | 1.00 | | | | 7.00 | | | −4.12 | | | 21.82 | | |
| Paternal intimacy 3 at T1 | 539 | 6.32 | | 0.98 | | | 7.00 | | | | 1.00 | | | | 7.00 | | | −2.32 | | | 7.24 | | |
| Paternal intimacy 4 at T1 | 541 | 6.84 | | 0.50 | | | 7.00 | | | | 3.00 | | | | 7.00 | | | −3.88 | | | 18.60 | | |
| Paternal intimacy 1 at T2 | 500 | 6.33 | | 1.06 | | | 7.00 | | | | 1.00 | | | | 7.00 | | | −2.54 | | | 7.99 | | |
| Paternal intimacy 2 at T2 | 500 | 6.30 | | 1.06 | | | 7.00 | | | | 1.00 | | | | 7.00 | | | −2.31 | | | 6.50 | | |
| Paternal intimacy 3 at T2 | 499 | 5.92 | | 1.21 | | | 6.00 | | | | 1.00 | | | | 7.00 | | | −1.46 | | | 2.06 | | |
| Paternal intimacy 4 at T2 | 499 | 6.49 | | 0.99 | | | 7.00 | | | | 1.00 | | | | 7.00 | | | −2.73 | | | 8.83 | | |
| Paternal intimacy 1 at T3 | 439 | 5.97 | | 1.33 | | | 6.00 | | | | 1.00 | | | | 7.00 | | | −1.67 | | | 2.55 | | |
| Paternal intimacy 2 at T3 | 439 | 5.72 | | 1.40 | | | 6.00 | | | | 1.00 | | | | 7.00 | | | −1.26 | | | 1.30 | | |
| Paternal intimacy 3 at T3 | 439 | 5.52 | | 1.52 | | | 6.00 | | | | 1.00 | | | | 7.00 | | | −1.17 | | | 0.80 | | |
| Paternal intimacy 4 at T3 | 439 | 5.99 | | 1.29 | | | 6.00 | | | | 1.00 | | | | 7.00 | | | −1.49 | | | 2.07 | | |
| Parents' average education level at T1 | 806 | 2.87 | | 0.86 | | | 3.00 | | | | 1.00 | | | | 4.00 | | | −0.36 | | | −0.78 | | |
| Assisted reproduction treatment | 885 | 0.50 | | 0.50 | | | 1.00 | | | | 0.00 | | | | 1.00 | | | 0.00 | | | −2.00 | | |
| Child's sex | 806 | 0.50 | | 0.50 | | | 1.00 | | | | 0.00 | | | | 1.00 | | | −0.02 | | | −2.00 | | |
| Reappraisal | 437 | 0.00 | | 1.06 | | | 0.06 | | | | −2.92 | | | | 2.30 | | | −0.31 | | | −0.03 | | |
| Suppression | 437 | 0.00 | | 1.12 | | | 0.00 | | | | −2.10 | | | | 2.78 | | | 0.19 | | | −0.62 | | |
| Rumination | 437 | 0.00 | | 1.06 | | | −0.17 | | | | −1.51 | | | | 3.84 | | | 0.87 | | | 0.60 | | |
| *Note.* T1 = Infancy; T2 = Middle childhood; T3 = Late adolescence. | | | | | |  | | |  |  | | |  | | |  | |  | |  | | |  |
|  | | | | | |  | | |  |  | | |  | | |  | |  | |  | | |  |

Supplementary **Table 1E.** Descriptive Statistics of Variables in Measurement Models of Adolescents’ Emotion Regulation Patterns.

|  |  | |  | |  | |  | |  | |  | |  | |  | |
| --- | --- | --- | --- | --- | --- | --- | --- | --- | --- | --- | --- | --- | --- | --- | --- | --- |
| Variable | | *n* | | *M* | | *SD* | | *Mdn* | | min | | max | | skewness | | kurtosis |
| Reappraisal 1 | | 437 | | 4.37 | | 1.54 | | 5.00 | | 1.00 | | 7.00 | | −0.33 | | −0.58 |
| Reappraisal 2 | | 433 | | 4.31 | | 1.60 | | 4.00 | | 1.00 | | 7.00 | | −0.21 | | −0.75 |
| Reappraisal 3 | | 437 | | 4.32 | | 1.67 | | 4.00 | | 1.00 | | 7.00 | | −0.11 | | −0.94 |
| Reappraisal 4 | | 435 | | 4.53 | | 1.48 | | 5.00 | | 1.00 | | 7.00 | | −0.41 | | −0.37 |
| Reappraisal 5 | | 433 | | 4.23 | | 1.46 | | 4.00 | | 1.00 | | 7.00 | | −0.25 | | −0.42 |
| Reappraisal 6 | | 433 | | 4.31 | | 1.48 | | 4.00 | | 1.00 | | 7.00 | | −0.20 | | −0.58 |
| Suppression 1 | | 435 | | 4.15 | | 1.71 | | 4.00 | | 1.00 | | 7.00 | | −0.11 | | −1.02 |
| Suppression 2 | | 435 | | 2.50 | | 1.46 | | 2.00 | | 1.00 | | 7.00 | | 1.00 | | 0.22 |
| Suppression 3 | | 432 | | 3.52 | | 1.78 | | 4.00 | | 1.00 | | 7.00 | | 0.18 | | −1.05 |
| Suppression 4 | | 436 | | 3.54 | | 1.61 | | 3.00 | | 1.00 | | 7.00 | | 0.31 | | −0.78 |
| Rumination 1 | | 435 | | 3.06 | | 1.03 | | 3.00 | | 1.00 | | 5.00 | | −0.05 | | −0.54 |
| Rumination 2 | | 436 | | 2.83 | | 1.10 | | 3.00 | | 1.00 | | 5.00 | | 0.12 | | −0.64 |
| Rumination 3 | | 436 | | 3.23 | | 1.06 | | 3.00 | | 1.00 | | 5.00 | | −0.16 | | −0.52 |
| Rumination 4 | | 437 | | 3.15 | | 1.21 | | 3.00 | | 1.00 | | 5.00 | | −0.11 | | −0.93 |
| Catastrophizing 1 | | 437 | | 2.36 | | 1.02 | | 2.00 | | 1.00 | | 5.00 | | 0.52 | | −0.22 |
| Catastrophizing 2 | | 436 | | 2.03 | | 0.98 | | 2.00 | | 1.00 | | 5.00 | | 0.78 | | 0.16 |
| Catastrophizing 3 | | 437 | | 1.51 | | 0.85 | | 1.00 | | 1.00 | | 5.00 | | 1.74 | | 2.77 |
| Catastrophizing 4 | | 437 | | 2.04 | | 1.03 | | 2.00 | | 1.00 | | 5.00 | | 0.79 | | −0.02 |
|  | | | | | | | | | | | | | | | | |
