# Supplementary material for: Developmental Stage-Specific Effects of Parenting on Adolescents’ Emotion Regulation: A Longitudinal Study From Infancy to Late Adolescence
Source: Front Psychol. 2021 Jun 4;12:582770. doi: 10.3389/fpsyg.2021.582770 (PMC8211896; doi:10.3389/fpsyg.2021.582770)
Supplement: Supplementary file 2 [file Table_2.docx]

Supplementary Material 2: **Zero-Order Correlations**

Supplementary **Table 2A.** Zero-Order Correlations Between Variables in Self-Reported Parental Autonomy Models.

|  |  |  |  |  |  |  |  |  |  |  |  |  |  |  |  |  |  |  |  |  |  |  |  |  |  |  |  |  |  |  |  |
| --- | --- | --- | --- | --- | --- | --- | --- | --- | --- | --- | --- | --- | --- | --- | --- | --- | --- | --- | --- | --- | --- | --- | --- | --- | --- | --- | --- | --- | --- | --- | --- |
|  | 1 | 2 | 3 | 4 | 5 | 6 | 7 | 8 | 9 | 10 | 11 | 12 | 13 | 14 | 15 | 16 | 17 | 18 | 19 | 20 | 21 | 22 | 23 | 24 | 25 | 26 | 27 | 28 | 29 | 30 |  |
| 1. Maternal autonomy 1 T1 | – |  |  |  |  |  |  |  |  |  |  |  |  |  |  |  |  |  |  |  |  |  |  |  |  |  |  |  |  |  |  |
| 2. Maternal autonomy 2 T1 | **.25** | – |  |  |  |  |  |  |  |  |  |  |  |  |  |  |  |  |  |  |  |  |  |  |  |  |  |  |  |  |  |
| 3. Maternal autonomy 3 T1 | **.19** | **.44** | – |  |  |  |  |  |  |  |  |  |  |  |  |  |  |  |  |  |  |  |  |  |  |  |  |  |  |  |  |
| 4. Maternal autonomy 4 T1 | **.25** | **.52** | **.40** | – |  |  |  |  |  |  |  |  |  |  |  |  |  |  |  |  |  |  |  |  |  |  |  |  |  |  |  |
| 5. Maternal autonomy 1 T2 | **.34** | **.12** | −.01 | −.02 | – |  |  |  |  |  |  |  |  |  |  |  |  |  |  |  |  |  |  |  |  |  |  |  |  |  |  |
| 6. Maternal autonomy 2 T2 | .06 | **.29** | **.25** | **.20** | **.12** | – |  |  |  |  |  |  |  |  |  |  |  |  |  |  |  |  |  |  |  |  |  |  |  |  |  |
| 7. Maternal autonomy 3 T2 | **.13** | **.24** | **.27** | **.11** | **.11** | **.43** | – |  |  |  |  |  |  |  |  |  |  |  |  |  |  |  |  |  |  |  |  |  |  |  |  |
| 8. Maternal autonomy 4 T2 | .09 | **.27** | **.30** | **.20** | **.15** | **.53** | **.53** | – |  |  |  |  |  |  |  |  |  |  |  |  |  |  |  |  |  |  |  |  |  |  |  |
| 9. Maternal autonomy 1 T3 | **.38** | **.12** | .07 | **.15** | **.34** | .09 | .06 | **.13** | – |  |  |  |  |  |  |  |  |  |  |  |  |  |  |  |  |  |  |  |  |  |  |
| 10. Maternal autonomy 2 T3 | **.16** | **.15** | **.13** | **.12** | **.14** | **.32** | **.20** | **.21** | **.20** | – |  |  |  |  |  |  |  |  |  |  |  |  |  |  |  |  |  |  |  |  |  |
| 11. Maternal autonomy 3 T3 | .10 | **.25** | **.18** | **.27** | .03 | **.22** | **.34** | **.22** | **.21** | **.36** | – |  |  |  |  |  |  |  |  |  |  |  |  |  |  |  |  |  |  |  |  |
| 12. Maternal autonomy 4 T1 | **.12** | **.25** | **.24** | **.26** | .09 | **.32** | **.35** | **.39** | **.23** | **.48** | **.48** | – |  |  |  |  |  |  |  |  |  |  |  |  |  |  |  |  |  |  |  |
| 13. Paternal autonomy 1 T1 | .**18** | .06 | .06 | .09 | .10 | .01 | .03 | .08 | .10 | .10 | .02 | .06 | – |  |  |  |  |  |  |  |  |  |  |  |  |  |  |  |  |  |  |
| 14. Paternal autonomy 2 T1 | .02 | **.10** | **.12** | **.15** | −.06 | .06 | .05 | .08 | −.04 | .03 | **.12** | −.04 | **.20** | – |  |  |  |  |  |  |  |  |  |  |  |  |  |  |  |  |  |
| 15. Paternal autonomy 3 T1 | −.01 | .05 | .08 | .05 | .01 | .07 | .06 | .02 | −.03 | .04 | **.14** | −.03 | **.19** | **.57** | – |  |  |  |  |  |  |  |  |  |  |  |  |  |  |  |  |
| 16. Paternal autonomy 4 T1 | .04 | .09 | **.11** | **.13** | −.01 | .05 | .05 | .10 | −.01 | .05 | **.12** | −.03 | **.23** | **.61** | **.53** | – |  |  |  |  |  |  |  |  |  |  |  |  |  |  |  |
| 17. Paternal autonomy 1 T2 | **.20** | .08 | .08 | .11 | .11 | .01 | −.04 | −.07 | .12 | .09 | −.05 | .00 | **.49** | .08 | **.13** | .05 | – |  |  |  |  |  |  |  |  |  |  |  |  |  |  |
| 18. Paternal autonomy 2 T2 | −.01 | **.11** | .02 | .07 | .07 | .12 | **.12** | .05 | **.17** | .10 | .11 | .08 | .08 | **.31** | **.22** | **.16** | **.13** | – |  |  |  |  |  |  |  |  |  |  |  |  |  |
| 19. Paternal autonomy 3 T2 | .02 | .12 | .00 | .08 | .05 | .10 | **.14** | **.13** | **.14** | .07 | .07 | .04 | **.15** | **.28** | **.34** | **.20** | **.18** | **.57** | – |  |  |  |  |  |  |  |  |  |  |  |  |
| 20. Paternal autonomy 4 T2 | .08 | .14 | .07 | .10 | .10 | **.15** | .04 | **.15** | **.21** | .05 | .06 | .01 | .06 | **.27** | **.26** | **.36** | .10 | **.58** | **.59** | – |  |  |  |  |  |  |  |  |  |  |  |
| 21. Paternal autonomy 1 T3 | .08 | −.07 | −.02 | −.02 | .02 | **−.13** | −.01 | −.02 | .01 | .05 | .03 | .07 | **.37** | −.04 | .06 | .02 | **.51** | .07 | .07 | .03 | – |  |  |  |  |  |  |  |  |  |  |
| 22. Paternal autonomy 2 T3 | **−.15** | .01 | −.11 | −.10 | −.11 | .01 | .00 | −.08 | −.10 | −.01 | .06 | −.01 | .01 | **.13** | .08 | **.16** | .00 | **.20** | .13 | **.23** | .10 | – |  |  |  |  |  |  |  |  |  |
| 23. Paternal autonomy 3 T3 | −.07 | −.01 | −.03 | −.07 | −.09 | .02 | .02 | −.01 | −.02 | .08 | **.18** | .04 | .07 | **.23** | **.19** | **.27** | .08 | **.27** | **.31** | **.27** | **.11** | **.43** | – |  |  |  |  |  |  |  |  |
| 24. Paternal autonomy 4 T3 | −.08 | .10 | .00 | .04 | −.02 | .04 | .01 | .10 | **.13** | .07 | **.16** | .08 | −.04 | **.15** | **.20** | **.25** | .07 | .13 | .13 | **.37** | **.14** | **.36** | **.48** | – |  |  |  |  |  |  |  |
| 25. Reappraisal | .04 | .07 | .06 | .06 | .03 | .03 | .06 | .02 | .04 | .03 | .06 | .08 | .05 | −.04 | −.01 | .00 | .00 | −.03 | −.02 | .11 | .02 | .09 | .06 | .07 | – |  |  |  |  |  |  |
| 26. Suppression | .04 | .02 | .04 | .03 | .02 | −.03 | −.05 | .06 | .01 | −.08 | −.01 | −.10 | .04 | −.06 | −.02 | **−.12** | −.07 | .00 | .06 | .04 | −.01 | −.03 | −.10 | −.08 | −.06 | – |  |  |  |  |  |
| 27. Rumination | .07 | −.04 | .00 | −.07 | −.05 | −.05 | −.04 | **−.11** | .05 | −.06 | −.08 | −.07 | −.06 | −.10 | −.04 | −.07 | −.09 | −.06 | −.07 | **−.16** | −.06 | **−.14** | **−.14** | **−.12** | **−.17** | **.13** | – |  |  |  |  |
| 28. Parents’ education level T1 | −.05 | −.05 | −.05 | −.01 | .06 | **−.11** | **−.13** | **−.12** | .01 | −.09 | −.04 | −.06 | **.19** | −.06 | −.07 | −.08 | **.15** | −.07 | −.05 | −.08 | .04 | −.01 | −.09 | −.12 | **.10** | −.05 | −.08 | – |  |  |  |
| 29. ART | −.05 | −.05 | −.03 | −.02 | .01 | .00 | .04 | .01 | .00 | −.03 | .01 | .01 | −.05 | −.01 | −.04 | −.02 | .06 | .01 | .01 | −.04 | .01 | .06 | −.03 | .03 | −.03 | .03 | **.09** | −.07 | – |  |  |
| 30. Child's sex | −.04 | .03 | .00 | .00 | .03 | .08 | .06 | .07 | **.10** | .01 | .05 | **.11** | .01 | .00 | .03 | .06 | .06 | −.02 | −.04 | −.09 | .08 | .02 | .09 | .04 | .08 | **−.28** | **.12** | .07 | .03 | – |  |
| *Note.* In bolded values, *p* < .050. T1 = Infancy; T2 = Middle childhood; T3 = Late adolescence; ART = Assisted reproduction treatment. | | | | | | | | | | | | | | | | | | | | | | | | | | | | | | | |
|  | | | | | | | | | | | | | | | | | | | | | | | | | | | | | | | |

Supplementary **Table 2B.** Zero-Order Correlations Between Variables in Partner-Reported Parental Autonomy Models.

|  | | | | | | | | | | | | | | | | | | | | | | | | | | | | | | |
| --- | --- | --- | --- | --- | --- | --- | --- | --- | --- | --- | --- | --- | --- | --- | --- | --- | --- | --- | --- | --- | --- | --- | --- | --- | --- | --- | --- | --- | --- | --- |
|  | 1 | 2 | 3 | 4 | 5 | 6 | 7 | 8 | 9 | 10 | 11 | 12 | 13 | 14 | 15 | 16 | 17 | 18 | 19 | 20 | 21 | 22 | 23 | 24 | 25 | 26 | 27 | 28 | 29 | 30 |
| 1. Maternal autonomy 1 T1 | – |  |  |  |  |  |  |  |  |  |  |  |  |  |  |  |  |  |  |  |  |  |  |  |  |  |  |  |  |  |
| 2. Maternal autonomy 2 T1 | **.16** | – |  |  |  |  |  |  |  |  |  |  |  |  |  |  |  |  |  |  |  |  |  |  |  |  |  |  |  |  |
| 3. Maternal autonomy 3 T1 | **.13** | **.55** |  |  |  |  |  |  |  |  |  |  |  |  |  |  |  |  |  |  |  |  |  |  |  |  |  |  |  |  |
| 4. Maternal autonomy 4 T1 | **.22** | **.45** | **.58** | – |  |  |  |  |  |  |  |  |  |  |  |  |  |  |  |  |  |  |  |  |  |  |  |  |  |  |
| 5. Maternal autonomy 1 T2 | **.50** | **.17** | .07 | **.13** | – |  |  |  |  |  |  |  |  |  |  |  |  |  |  |  |  |  |  |  |  |  |  |  |  |  |
| 6. Maternal autonomy 2 T2 | **.18** | **.26** | **.31** | **.22** | **.19** | – |  |  |  |  |  |  |  |  |  |  |  |  |  |  |  |  |  |  |  |  |  |  |  |  |
| 7. Maternal autonomy 3 T2 | **.24** | **.18** | **.30** | **.35** | **.26** | **.60** | – |  |  |  |  |  |  |  |  |  |  |  |  |  |  |  |  |  |  |  |  |  |  |  |
| 8. Maternal autonomy 4 T2 | **.19** | **.19** | **.29** | **.27** | **.20** | **.44** | **.48** | – |  |  |  |  |  |  |  |  |  |  |  |  |  |  |  |  |  |  |  |  |  |  |
| 9. Maternal autonomy 1 T3 | **.29** | **.18** | .10 | .10 | **.38** | **.24** | .12 | **.19** | – |  |  |  |  |  |  |  |  |  |  |  |  |  |  |  |  |  |  |  |  |  |
| 10. Maternal autonomy 2 T3 | **.15** | **.27** | **.27** | **.20** | **.18** | **.39** | **.25** | **.24** | **.43** | – |  |  |  |  |  |  |  |  |  |  |  |  |  |  |  |  |  |  |  |  |
| 11. Maternal autonomy 3 T3 | **.13** | **.22** | **.35** | **.25** | **.20** | **.30** | **.38** | **.27** | **.35** | **.66** | – |  |  |  |  |  |  |  |  |  |  |  |  |  |  |  |  |  |  |  |
| 12. Maternal autonomy 4 T1 | .12 | **.26** | **.30** | **.18** | .10 | **.20** | **.19** | **.28** | **.35** | **.66** | **.72** | – |  |  |  |  |  |  |  |  |  |  |  |  |  |  |  |  |  |  |
| 13. Paternal autonomy 1 T1 | **.16** | .03 | −.03 | .01 | .06 | .04 | .00 | .00 | **.14** | **.15** | **.18** | **.21** | – |  |  |  |  |  |  |  |  |  |  |  |  |  |  |  |  |  |
| 14. Paternal autonomy 2 T1 | **.11** | .06 | .07 | **.09** | **.14** | .10 | **.16** | **.18** | .10 | .03 | .10 | .09 | **.38** | – |  |  |  |  |  |  |  |  |  |  |  |  |  |  |  |  |
| 15. Paternal autonomy 3 T1 | .08 | .06 | .06 | **.09** | **.14** | .08 | .12 | **.20** | .06 | −.06 | .00 | −.02 | **.36** | **.63** | – |  |  |  |  |  |  |  |  |  |  |  |  |  |  |  |
| 16. Paternal autonomy 4 T1 | **.11** | .08 | .06 | **.12** | .12 | **.15** | **.19** | **.19** | .10 | −.03 | .07 | .07 | **.40** | **.65** | **.63** | – |  |  |  |  |  |  |  |  |  |  |  |  |  |  |
| 17. Paternal autonomy 1 T2 | −.02 | .10 | −.03 | .01 | .07 | .04 | .00 | .01 | .08 | .00 | .10 | .04 | **.19** | **.11** | .10 | .03 | – |  |  |  |  |  |  |  |  |  |  |  |  |  |
| 18. Paternal autonomy 2 T2 | .08 | **.15** | .04 | .08 | .03 | **.17** | **.17** | **.13** | .11 | −.01 | .08 | −.04 | **.23** | **.22** | **.23** | **.20** | **.38** | – |  |  |  |  |  |  |  |  |  |  |  |  |
| 19. Paternal autonomy 3 T2 | .05 | **.13** | .01 | .06 | .02 | .08 | .10 | .10 | −.07 | −.12 | .00 | −.05 | **.17** | **.17** | **.25** | **.13** | **.35** | **.52** | – |  |  |  |  |  |  |  |  |  |  |  |
| 20. Paternal autonomy 4 T2 | .11 | **.14** | .00 | .07 | .03 | .05 | .09 | **.12** | .08 | .00 | .05 | .03 | **.17** | **.24** | **.26** | **.22** | **.32** | **.57** | **.65** | – |  |  |  |  |  |  |  |  |  |  |
| 21. Paternal autonomy 1 T3 | −.03 | .06 | .09 | −.02 | −.03 | .08 | **.16** | .08 | .00 | −.03 | .07 | .04 | **.19** | .09 | **.13** | .10 | **.23** | **.23** | **.16** | **.14** | – |  |  |  |  |  |  |  |  |  |
| 22. Paternal autonomy 2 T3 | .08 | .05 | .08 | .00 | .05 | .04 | **.15** | .02 | −.01 | .00 | **.12** | .06 | **.13** | **.29** | **.29** | **.19** | **.12** | **.38** | **.33** | **.28** | **.46** | – |  |  |  |  |  |  |  |  |
| 23. Paternal autonomy 3 T3 | .04 | −.01 | .02 | .02 | .05 | .05 | **.16** | .06 | .01 | −.01 | .06 | .03 | **.12** | **.18** | **.27** | **.13** | **.18** | **.34** | **.42** | **.31** | **.46** | **.71** | – |  |  |  |  |  |  |  |
| 24. Paternal autonomy 4 T3 | .09 | −.02 | .07 | .00 | .06 | .05 | .13 | .10 | .02 | .09 | **.16** | .08 | **.12** | **.18** | **.24** | **.17** | **.16** | **.34** | **.24** | **.26** | **.47** | **.67** | **.68** | – |  |  |  |  |  |  |
| 25. Reappraisal | .05 | .07 | −.02 | −.09 | −.02 | −.02 | .08 | **.16** | .04 | .08 | **.12** | .11 | .03 | .07 | .09 | .07 | .02 | −.03 | −.03 | −.04 | .03 | −.02 | .01 | .06 | – |  |  |  |  |  |
| 26. Suppression | .01 | −.09 | −.05 | −.02 | −.06 | −.02 | .08 | .04 | −.04 | −.03 | .05 | −.06 | **.12** | .02 | −.09 | −.02 | .01 | −.01 | .00 | .02 | .06 | −.02 | −.02 | −.04 | −.06 | – |  |  |  |  |
| 27. Rumination | .06 | −.01 | .02 | .09 | −.03 | −.04 | .03 | −.06 | −.03 | −.07 | −.06 | .04 | −.04 | −.06 | **−.11** | −.02 | −.09 | −.08 | −.04 | .00 | **−.16** | **−.14** | −.08 | −.09 | **−.17** | **.13** | – |  |  |  |
| 28. Parents’ education level T1 | **.13** | **−.10** | **−.15** | **−.15** | **.13** | .02 | .06 | −.03 | −.03 | **−.11** | −.06 | **−.12** | .05 | .03 | .01 | .05 | .01 | −.03 | −.01 | −.01 | .01 | .05 | .08 | .04 | **.10** | −.05 | −.08 | – |  |  |
| 29. ART | −.03 | −.05 | .06 | .02 | .00 | .05 | .05 | −.04 | −.03 | .02 | .07 | .02 | −.03 | .02 | .00 | −.02 | −.01 | .02 | .05 | −.03 | −.01 | −.04 | −.02 | −.05 | −.03 | .03 | **.09** | −.07 | – |  |
| 30. Child's sex | .03 | .05 | .02 | .04 | .06 | .02 | −.03 | −.02 | .03 | .08 | .05 | .09 | −.04 | .01 | .00 | .01 | −.04 | .03 | −.01 | .03 | −.01 | .02 | .05 | .05 | .08 | **−.28** | **.12** | .07 | .03 | – |
| *Note.* In bolded values, *p* < .050. T1 = Infancy; T2 = Middle childhood; T3 = Late adolescence; ART = Assisted reproduction treatment. | | | | | | | | | | | | | | | | | | | | | | | | | | | | | | |
|  | | | | | | | | | | | | | | | | | | | | | | | | | | | | | | |

Supplementary **Table 2C.** Zero-Order Correlations Between Variables in Self-Reported Parental Intimacy Models.

|  | | | | | | | | | | | | | | | | | | | | | | | | | | | | | | |  |
| --- | --- | --- | --- | --- | --- | --- | --- | --- | --- | --- | --- | --- | --- | --- | --- | --- | --- | --- | --- | --- | --- | --- | --- | --- | --- | --- | --- | --- | --- | --- | --- |
|  | 1 | 2 | 3 | 4 | 5 | 6 | 7 | 8 | 9 | 10 | 11 | 12 | 13 | 14 | 15 | 16 | 17 | 18 | 19 | 20 | 21 | 22 | 23 | 24 | 25 | 26 | 27 | 28 | 29 | 30 | |
| 1. Maternal intimacy 1 T1 | – |  |  |  |  |  |  |  |  |  |  |  |  |  |  |  |  |  |  |  |  |  |  |  |  |  |  |  |  |  | |
| 2. Maternal intimacy 2 T1 | **.53** | – |  |  |  |  |  |  |  |  |  |  |  |  |  |  |  |  |  |  |  |  |  |  |  |  |  |  |  |  | |
| 3. Maternal intimacy 3 T1 | **.29** | **.46** | – |  |  |  |  |  |  |  |  |  |  |  |  |  |  |  |  |  |  |  |  |  |  |  |  |  |  |  | |
| 4. Maternal intimacy 4 T1 | **.44** | **.50** | **.48** | – |  |  |  |  |  |  |  |  |  |  |  |  |  |  |  |  |  |  |  |  |  |  |  |  |  |  | |
| 5. Maternal intimacy 1 T2 | **.36** | **.29** | **.26** | **.29** | – |  |  |  |  |  |  |  |  |  |  |  |  |  |  |  |  |  |  |  |  |  |  |  |  |  | |
| 6. Maternal intimacy 2 T2 | **.30** | **.30** | **.23** | **.31** | **.43** | – |  |  |  |  |  |  |  |  |  |  |  |  |  |  |  |  |  |  |  |  |  |  |  |  | |
| 7. Maternal intimacy 3 T2 | **.18** | **.19** | **.34** | **.12** | **.30** | **.32** | – |  |  |  |  |  |  |  |  |  |  |  |  |  |  |  |  |  |  |  |  |  |  |  | |
| 8. Maternal intimacy 4 T2 | **.18** | **.26** | **.18** | **.28** | **.39** | **.71** | **.32** | – |  |  |  |  |  |  |  |  |  |  |  |  |  |  |  |  |  |  |  |  |  |  | |
| 9. Maternal intimacy 1 T3 | **.22** | **.14** | **.14** | .06 | **.18** | **.12** | .05 | **.11** | – |  |  |  |  |  |  |  |  |  |  |  |  |  |  |  |  |  |  |  |  |  | |
| 10. Maternal intimacy 2 T3 | **.19** | **.20** | **.17** | .10 | **.27** | **.33** | **.18** | **.27** | **.23** | – |  |  |  |  |  |  |  |  |  |  |  |  |  |  |  |  |  |  |  |  | |
| 11. Maternal intimacy 3 T3 | .06 | .11 | **.13** | .00 | **.11** | **.15** | **.17** | **.14** | **.08** | **.33** | – |  |  |  |  |  |  |  |  |  |  |  |  |  |  |  |  |  |  |  | |
| 12. Maternal intimacy 4 T1 | .10 | **.28** | **.16** | **.15** | **.27** | **.30** | **.14** | **.29** | **.34** | **.42** | **.33** | – |  |  |  |  |  |  |  |  |  |  |  |  |  |  |  |  |  |  | |
| 13. Paternal intimacy 1 T1 | **.09** | .06 | .07 | −.01 | −.04 | .05 | .05 | .03 | .09 | .08 | −.01 | −.05 | – |  |  |  |  |  |  |  |  |  |  |  |  |  |  |  |  |  | |
| 14. Paternal intimacy 2 T1 | .07 | .01 | .01 | −.01 | −.03 | .02 | .04 | .10 | .06 | .07 | .02 | −.04 | **.44** | – |  |  |  |  |  |  |  |  |  |  |  |  |  |  |  |  | |
| 15. Paternal intimacy 3 T1 | .06 | .01 | .01 | .03 | .03 | .05 | −.01 | .01 | **.11** | −.02 | −.03 | **−.12** | **.42** | **.61** | – |  |  |  |  |  |  |  |  |  |  |  |  |  |  |  | |
| 16. Paternal intimacy 4 T1 | .07 | .04 | .02 | .00 | −.06 | .00 | .03 | .02 | **.14** | .04 | .06 | −.04 | **.58** | **.55** | **.43** | – |  |  |  |  |  |  |  |  |  |  |  |  |  |  | |
| 17. Paternal intimacy 1 T2 | .02 | −.07 | .10 | −.06 | .02 | −.03 | .07 | .04 | **.23** | .13 | .09 | **.21** | **.34** | **.22** | **.29** | **.31** | – |  |  |  |  |  |  |  |  |  |  |  |  |  | |
| 18. Paternal intimacy 2 T2 | .02 | .00 | **.16** | .06 | .09 | .10 | .08 | **.16** | .01 | **.19** | **.19** | .03 | **.24** | **.25** | **.25** | **.36** | **.36** | – |  |  |  |  |  |  |  |  |  |  |  |  | |
| 19. Paternal intimacy 3 T2 | **.13** | .11 | .11 | .10 | .09 | .07 | .07 | .11 | .09 | **.14** | .11 | −.03 | **.31** | **.23** | **.36** | **.19** | **.31** | **.53** | – |  |  |  |  |  |  |  |  |  |  |  | |
| 20. Paternal intimacy 4 T2 | −.03 | −.01 | .04 | −.04 | .00 | −.03 | .07 | .01 | **.14** | .11 | .09 | .12 | **.27** | **.21** | **.21** | **.35** | **.55** | **.54** | **.39** | – |  |  |  |  |  |  |  |  |  |  | |
| 21. Paternal intimacy 1 T3 | .06 | .04 | .01 | −.02 | **.15** | .07 | .05 | .07 | **.28** | .03 | .11 | **.13** | **.16** | **.13** | .12 | **.19** | **.43** | **.16** | **.17** | **.30** | – |  |  |  |  |  |  |  |  |  | |
| 22. Paternal intimacy 2 T3 | .00 | −.07 | −.05 | −.02 | .06 | .01 | .03 | −.01 | .01 | .09 | .07 | −.07 | **.14** | .04 | **.13** | .08 | .14 | **.29** | **.27** | **.17** | **.36** | – |  |  |  |  |  |  |  |  | |
| 23. Paternal intimacy 3 T3 | −.05 | −.07 | −.11 | −.07 | −.01 | −.02 | −.03 | −.04 | .08 | **.13** | .10 | −.03 | .12 | .08 | **.17** | .07 | **.16** | **.20** | **.35** | .09 | **.37** | **.54** | – |  |  |  |  |  |  |  | |
| 24. Paternal intimacy 4 T3 | −.03 | −.03 | −.06 | −.02 | .03 | .03 | .00 | .03 | **.16** | **.14** | **.14** | .08 | **.14** | .10 | **.14** | **.21** | **.34** | **.21** | **.26** | **.33** | **.60** | **.51** | **.54** | – |  |  |  |  |  |  | |
| 25. Reappraisal | .05 | .00 | .03 | −.01 | .08 | .06 | .00 | .04 | −.03 | .03 | .06 | .04 | .04 | −.04 | .01 | .05 | .00 | −.01 | .06 | .00 | .06 | .10 | .06 | .04 | – |  |  |  |  |  | |
| 26. Suppression | .03 | .04 | −.05 | .04 | −.04 | .01 | −.01 | −.04 | .00 | −.06 | −.08 | −.10 | −.06 | −.09 | −.02 | −.05 | .00 | −.04 | −.01 | .03 | −.05 | −.13 | −.11 | −.11 | −.06 | – |  |  |  |  | |
| 27. Rumination | −.03 | −.04 | −.07 | −.06 | −.03 | .00 | .03 | −.06 | −.07 | .03 | .00 | −.08 | −.05 | −.03 | −.03 | −.01 | −.04 | .06 | −.09 | .00 | −.09 | −.04 | −.05 | −.07 | **−.17** | **.13** | – |  |  |  | |
| 28. Parents’ education level T1 | −.04 | .00 | −.07 | −.03 | −.06 | **−.09** | −.02 | −.06 | .10 | −.04 | −.08 | −.05 | .00 | −.02 | −.06 | .02 | .00 | .01 | −.05 | .06 | .04 | −.06 | −.06 | .00 | **.10** | −.05 | −.08 | – |  |  | |
| 29. ART | .05 | .03 | .05 | .03 | .07 | .04 | −.03 | .01 | .08 | .05 | .03 | .04 | .01 | −.03 | .00 | −.02 | .05 | −.01 | .07 | .04 | −.04 | .06 | .05 | −.04 | −.03 | .03 | **.09** | −.07 | – |  | |
| 30. Child's sex | .00 | .00 | .03 | −.01 | .00 | .05 | .06 | −.01 | .05 | .02 | .07 | −.01 | .05 | .07 | .07 | .08 | −.02 | .05 | .05 | .04 | .07 | **.17** | .10 | **.13** | .08 | **−.28** | **.12** | .07 | .03 | – | |
| *Note.* In bolded values, *p* < .050. T1 = Infancy; T2 = Middle childhood; T3 = Late adolescence; ART = Assisted reproduction treatment. | | | | | | | | | | | | | | | | | | | | | | | | | | | | | | |  |

Supplementary **Table 2D.** Zero-Order Correlations Between Variables in Partner-Reported Parental Intimacy Models.

|  | | | | | | | | | | | | | | | | | | | | | | | | | | | | | | |  |
| --- | --- | --- | --- | --- | --- | --- | --- | --- | --- | --- | --- | --- | --- | --- | --- | --- | --- | --- | --- | --- | --- | --- | --- | --- | --- | --- | --- | --- | --- | --- | --- |
|  | 1 | 2 | 3 | 4 | 5 | 6 | 7 | 8 | 9 | 10 | 11 | 12 | 13 | 14 | 15 | 16 | 17 | 18 | 19 | 20 | 21 | 22 | 23 | 24 | 25 | 26 | 27 | 28 | 29 | 30 | |
| 1. Maternal intimacy 1 T1 | – |  |  |  |  |  |  |  |  |  |  |  |  |  |  |  |  |  |  |  |  |  |  |  |  |  |  |  |  |  | |
| 2. Maternal intimacy 2 T1 | **.33** | – |  |  |  |  |  |  |  |  |  |  |  |  |  |  |  |  |  |  |  |  |  |  |  |  |  |  |  |  | |
| 3. Maternal intimacy 3 T1 | **.29** | **.41** | – |  |  |  |  |  |  |  |  |  |  |  |  |  |  |  |  |  |  |  |  |  |  |  |  |  |  |  | |
| 4. Maternal intimacy 4 T1 | **.74** | **.34** | **.29** | – |  |  |  |  |  |  |  |  |  |  |  |  |  |  |  |  |  |  |  |  |  |  |  |  |  |  | |
| 5. Maternal intimacy 1 T2 | **.19** | .10 | **.17** | **.28** | – |  |  |  |  |  |  |  |  |  |  |  |  |  |  |  |  |  |  |  |  |  |  |  |  |  | |
| 6. Maternal intimacy 2 T2 | **.23** | **.32** | **.18** | **.37** | **.57** | – |  |  |  |  |  |  |  |  |  |  |  |  |  |  |  |  |  |  |  |  |  |  |  |  | |
| 7. Maternal intimacy 3 T2 | **.27** | **.21** | **.29** | **.21** | **.40** | **.60** | – |  |  |  |  |  |  |  |  |  |  |  |  |  |  |  |  |  |  |  |  |  |  |  | |
| 8. Maternal intimacy 4 T2 | **.20** | **.15** | **.14** | **.29** | **.64** | **.64** | **.38** | – |  |  |  |  |  |  |  |  |  |  |  |  |  |  |  |  |  |  |  |  |  |  | |
| 9. Maternal intimacy 1 T3 | .11 | **.15** | .09 | **.18** | **.24** | **.20** | .14 | **.20** | – |  |  |  |  |  |  |  |  |  |  |  |  |  |  |  |  |  |  |  |  |  | |
| 10. Maternal intimacy 2 T3 | **.13** | .07 | .07 | **.17** | .08 | **.34** | **.24** | **.15** | **.60** | – |  |  |  |  |  |  |  |  |  |  |  |  |  |  |  |  |  |  |  |  | |
| 11. Maternal intimacy 3 T3 | .03 | .10 | .06 | .10 | .01 | **.19** | **.21** | .07 | **.51** | **.63** | – |  |  |  |  |  |  |  |  |  |  |  |  |  |  |  |  |  |  |  | |
| 12. Maternal intimacy 4 T1 | **.16** | **.21** | **.15** | **.16** | **.23** | **.28** | **.22** | **.31** | **.78** | **.72** | **.57** | – |  |  |  |  |  |  |  |  |  |  |  |  |  |  |  |  |  |  | |
| 13. Paternal intimacy 1 T1 | .06 | .01 | .08 | .03 | −.01 | .01 | **.13** | −.01 | .02 | .07 | .00 | .04 | – |  |  |  |  |  |  |  |  |  |  |  |  |  |  |  |  |  | |
| 14. Paternal intimacy 2 T1 | **.18** | **.09** | **.13** | **.12** | −.02 | .05 | .13 | .04 | **.13** | **.13** | −.05 | .09 | **.60** | – |  |  |  |  |  |  |  |  |  |  |  |  |  |  |  |  | |
| 15. Paternal intimacy 3 T1 | **.14** | .04 | .04 | .07 | .01 | .03 | **.15** | .00 | **.14** | **.14** | .05 | .10 | **.59** | **.60** | – |  |  |  |  |  |  |  |  |  |  |  |  |  |  |  | |
| 16. Paternal intimacy 4 T1 | **.16** | .07 | .07 | .08 | −.05 | −.01 | .11 | .00 | .06 | .05 | −.03 | .01 | **.63** | **.71** | **.55** | – |  |  |  |  |  |  |  |  |  |  |  |  |  |  | |
| 17. Paternal intimacy 1 T2 | .06 | **.17** | **.14** | .07 | **.16** | .10 | .11 | **.14** | .07 | .03 | .05 | .06 | **.34** | **.22** | **.29** | **.23** | – |  |  |  |  |  |  |  |  |  |  |  |  |  | |
| 18. Paternal intimacy 2 T2 | .02 | .07 | .10 | .03 | .11 | .06 | .05 | **.14** | .03 | −.02 | −.04 | .01 | **.34** | **.29** | **.31** | **.30** | **.58** | – |  |  |  |  |  |  |  |  |  |  |  |  | |
| 19. Paternal intimacy 3 T2 | .06 | .02 | .01 | .00 | .00 | −.01 | −.01 | .01 | −.04 | .05 | −.03 | −.03 | **.23** | **.18** | **.41** | **.24** | **.45** | **.50** | – |  |  |  |  |  |  |  |  |  |  |  | |
| 20. Paternal intimacy 4 T2 | .02 | .05 | .03 | .05 | .11 | .08 | .05 | .10 | .05 | −.02 | −.04 | .03 | **.28** | **.23** | **.24** | **.27** | **.61** | **.71** | **.42** | – |  |  |  |  |  |  |  |  |  |  | |
| 21. Paternal intimacy 1 T3 | **−.12** | −.06 | .02 | **−.11** | −.01 | −.08 | .05 | .04 | **.19** | **.18** | **.15** | **.19** | **.25** | **.12** | **.15** | **.12** | **.45** | **.32** | **.31** | **.36** | – |  |  |  |  |  |  |  |  |  | |
| 22. Paternal intimacy 2 T3 | −.06 | −.03 | .04 | −.02 | .09 | .10 | **.23** | .11 | **.18** | **.14** | .05 | **.15** | **.27** | **.25** | **.21** | **.22** | **.36** | **.33** | **.34** | **.32** | **.72** | – |  |  |  |  |  |  |  |  | |
| 23. Paternal intimacy 3 T3 | −.04 | .00 | .00 | −.02 | .04 | .07 | .10 | .09 | **.24** | **.23** | .07 | **.19** | **.21** | **.17** | **.31** | **.15** | **.33** | **.33** | **.46** | **.27** | **.62** | **.69** | – |  |  |  |  |  |  |  | |
| 24. Paternal intimacy 4 T3 | −.05 | −.03 | .00 | .00 | .11 | .09 | **.17** | **.17** | **.22** | **.16** | .02 | **.24** | **.29** | **.23** | **.23** | **.21** | **.38** | **.35** | **.40** | **.34** | **.69** | **.84** | **.68** | – |  |  |  |  |  |  | |
| 25. Reappraisal | .04 | .07 | .05 | .04 | .06 | .09 | .13 | .05 | .08 | .10 | .13 | .11 | .08 | .02 | .05 | −.02 | .03 | .03 | .01 | .05 | **.16** | .10 | **.13** | .08 | – |  |  |  |  |  | |
| 26. Suppression | .00 | .02 | −.01 | −.03 | −.08 | −.04 | −.01 | .03 | −.04 | .01 | −.01 | −.01 | .00 | −.04 | .00 | .03 | −.09 | −.01 | .05 | −.05 | −.01 | −.02 | .00 | −.03 | −.06 | – |  |  |  |  | |
| 27. Rumination | −.07 | −.08 | −.04 | −.05 | .08 | −.01 | −.13 | .00 | −.02 | .00 | .02 | −.09 | −.05 | .03 | −.01 | .03 | −.05 | −.04 | −.01 | −.09 | **−.14** | −.10 | **−.13** | **−.15** | **−.17** | **.13** | – |  |  |  | |
| 28. Parents’ education level T1 | .03 | .06 | −.07 | .05 | .07 | .00 | −.06 | .05 | .02 | .01 | .02 | .06 | .00 | −.03 | .00 | −.03 | .04 | .05 | .01 | .04 | .08 | .00 | .02 | −.04 | **.10** | −.05 | −.08 | – |  |  | |
| 29. ART | −.03 | .08 | .07 | −.05 | .03 | −.03 | .03 | .01 | .08 | **.11** | .01 | .01 | **.13** | **.11** | **.11** | **.09** | **.17** | **.10** | −.03 | .09 | .08 | .06 | .04 | .03 | −.03 | .03 | **.09** | −.07 | – |  | |
| 30. Child's sex | .02 | .00 | .01 | .01 | −.01 | −.04 | −.04 | −.02 | .03 | −.02 | .01 | .04 | .04 | .03 | .06 | .07 | −.01 | .00 | .02 | −.02 | .07 | .07 | .07 | .04 | .08 | **−.28** | **.12** | .07 | .03 | – | |
| *Note.* In bolded values, *p* < .050. T1 = Infancy; T2 = Middle childhood; T3 = Late adolescence; ART = Assisted reproduction treatment. | | | | | | | | | | | | | | | | | | | | | | | | | | | | | | | |
|  | | | | | | | | | | | | | | | | | | | | | | | | | | | | | | | |

Supplementary **Table 2E.** Zero-Order Correlations Between Variables in Measurement Models of Adolescents’ Emotion Regulation Patterns.

|  | | | | | | | | | | | | | | | | | | |
| --- | --- | --- | --- | --- | --- | --- | --- | --- | --- | --- | --- | --- | --- | --- | --- | --- | --- | --- |
|  | 1 | 2 | 3 | 4 | 5 | 6 | 7 | 8 | 9 | 10 | 11 | 12 | 13 | 14 | 15 | 16 | 17 | 18 |
| 1. Reappraisal 1 | – |  |  |  |  |  |  |  |  |  |  |  |  |  |  |  |  |  |
| 2. Reappraisal 2 | **.71** | – |  |  |  |  |  |  |  |  |  |  |  |  |  |  |  |  |
| 3. Reappraisal 3 | **.22** | **.32** | – |  |  |  |  |  |  |  |  |  |  |  |  |  |  |  |
| 4. Reappraisal 4 | **.55** | **.56** | **.33** | – |  |  |  |  |  |  |  |  |  |  |  |  |  |  |
| 5. Reappraisal 5 | **.49** | **.49** | **.32** | **.64** | – |  |  |  |  |  |  |  |  |  |  |  |  |  |
| 6. Reappraisal 6 | **.55** | **.56** | **.31** | **.71** | **.67** | – |  |  |  |  |  |  |  |  |  |  |  |  |
| 7. Suppression 1 | −.03 | −.06 | .02 | −.08 | −.02 | −.05 | – |  |  |  |  |  |  |  |  |  |  |  |
| 8. Suppression 2 | **−.10** | −.11 | −.08 | **−.15** | .02 | −.09 | **.45** | – |  |  |  |  |  |  |  |  |  |  |
| 9. Suppression 3 | −.02 | −.03 | .05 | −.06 | .00 | −.09 | **.57** | **.48** | – |  |  |  |  |  |  |  |  |  |
| 10. Suppression 4 | −.02 | −.03 | .00 | −.02 | .04 | .01 | **.47** | **.34** | **.50** | – |  |  |  |  |  |  |  |  |
| 11. Rumination 1 | .04 | −.02 | **−.13** | .03 | .06 | −.02 | .00 | .07 | −.04 | .07 | – |  |  |  |  |  |  |  |
| 12. Rumination 2 | −.04 | −.06 | **−.24** | −.02 | .01 | −.03 | .00 | .04 | −.02 | .09 | **.55** | – |  |  |  |  |  |  |
| 13. Rumination 3 | **.10** | .06 | .02 | **.19** | **.14** | **.15** | **−.24** | **−.16** | **−.25** | **−.14** | **.44** | **.32** | – |  |  |  |  |  |
| 14. Rumination 4 | **.16** | **.10** | −.03 | **.16** | **.13** | **.17** | **−.12** | **−.10** | **−.21** | −.03 | **.52** | **.38** | **.53** | – |  |  |  |  |
| 15. Catastrophizing 1 | **−.08** | −.08 | **−.22** | **−.14** | **−.11** | **−.18** | .06 | **.17** | **.11** | .06 | **.29** | **.33** | **.10** | **.13** | – |  |  |  |
| 16. Catastrophizing 2 | **−.15** | **−.13** | **−.20** | **−.15** | −.02 | **−.16** | .08 | **.22** | .05 | **.12** | **.41** | **.53** | **.16** | **.26** | **.58** | – |  |  |
| 17. Catastrophizing 3 | **−.14** | **−.10** | **−.20** | **−.19** | −.05 | **−.16** | .07 | **.27** | .05 | **.10** | **.28** | **.36** | **.10** | **.17** | **.47** | **.61** | – |  |
| 18. Catastrophizing 4 | **−.13** | **−.12** | **−.20** | **−.12** | −.05 | **−.17** | .06 | **.14** | .04 | **.13** | **.40** | **.56** | **.15** | **.24** | **.46** | **.70** | **.57** | – |
| *Note.* In bolded values, *p* < .050. | | | | | | | | | | | | | | | | | | |
|  | | | | | | | | | | | | | | | | | | |
