# Supplementary material for: Developmental Stage-Specific Effects of Parenting on Adolescents’ Emotion Regulation: A Longitudinal Study From Infancy to Late Adolescence
Source: Front Psychol. 2021 Jun 4;12:582770. doi: 10.3389/fpsyg.2021.582770 (PMC8211896; doi:10.3389/fpsyg.2021.582770)
Supplement: Supplementary file 3 [file Table_3.docx]

**Supplementary Material 3.** Testing Measurement Models of Parental Autonomy and Intimacy.

|  |  |  |  |  |  |  |  |
| --- | --- | --- | --- | --- | --- | --- | --- |
| Model | *df* | Scaled  χ^2^ | CFI | RMSEA | SRMR | Scaled  ∆χ^2^ test | *p* |
| **Self-Reported Parental Autonomy** |  |  |  |  |  |  |  |
| ***Time Invariance*** |  |  |  |  |  |  |  |
| Configural time invariance | 213 | 256.48 | .982 | .015 | .051 |  |  |
| Weak time invariance | 229 | 309.58 | .966 | .020 | .088 | 43.10 | < .001 |
| Strong time invariance | 241 | 347.35 | .954 | .022 | .089 | 37.04 | < .001 |
| Strict time invariance | 257 | 698.88 | .783 | .048 | .254 | 132.14 | < .001 |
| ***Factorial Invariance Between Mothering and Fathering*** |  |  |  |  |  |  |  |
| Configural time invariance + |  |  |  |  |  |  |  |
| Configural factorial invariance | 213 | 256.48 | .982 | .015 | .051 |  |  |
| Weak factorial invariance | 225 | 289.01 | .973 | .018 | .087 | 27.32 | .007 |
| Strong factorial invariance | 234 | 317.77 | .964 | .020 | .088 | 28.23 | < .001 |
| Strict factorial invariance | 246 | 317.43 | .965 | .020 | .114 | 11.62 | .477 |
| ***Fixed Error Term Correlations of Corresponding Indicators*** |  |  |  |  |  |  |  |
| Configural time invariance + configural factorial invariance | 213 | 256.48 | .982 | .015 | .051 |  |  |
| Fixing the correlations of indicator error terms that do not weaken model fit to the same value or zero | 230 | 273.75 | .981 | .015 | .053 | 17.92 | .394 |
| ***Autoregressive Cross-Lagged Models*** |  |  |  |  |  |  |  |
| Second-order autoregressive cross-lagged model | 232 | 277.76 | .981 | .015 | .054 |  |  |
| First-order autoregressive cross-lagged model | 234 | 293.69 | .975 | .017 | .058 | 17.48 | < .001 |
| **Partner-Reported Parental Autonomy** |  |  |  |  |  |  |  |
| ***Time Invariance*** |  |  |  |  |  |  |  |
| Configural time invariance | 213 | 278.45 | .981 | .019 | .052 |  |  |
| Weak time invariance | 229 | 391.16 | .950 | .029 | .144 | 78.19 | < .001 |
| Strong time invariance | 241 | 444.75 | .936 | .032 | . 147 | 53.01 | < .001 |
| Strict time invariance | 257 | 474.65 | .920 | .035 | .195 | 29.64 | .020 |
| ***Factorial Invariance Between Mothering and Fathering*** |  |  |  |  |  |  |  |
| Configural time invariance + |  |  |  |  |  |  |  |
| Configural factorial invariance | 213 | 278.45 | .981 | .019 | .052 |  |  |
| Weak factorial invariance | 225 | 340.57 | .964 | .025 | .126 | 44.90 | < .001 |
| Strong factorial invariance | 234 | 409.65 | .945 | .030 | .133 | 66.02 | < .001 |
| Strict factorial invariance | 246 | 479.60 | .917 | .036 | .185 | 35.39 | < .001 |
| ***Fixed Error Term Correlations of Corresponding Indicators*** |  |  |  |  |  |  |  |
| Configural time invariance + configural factorial invariance | 213 | 278.45 | .981 | .019 | .052 |  |  |
| Fixing the correlations of indicator error terms that do not weaken model fit to the same value or zero | 230 | 292.40 | .981 | .018 | .053 | 15.68 | .547 |
| ***Autoregressive Cross-Lagged Models*** |  |  |  |  |  |  |  |
| Second-order autoregressive cross-lagged model | 232 | 292.96 | .981 | .017 | .053 |  |  |
| First-order autoregressive cross-lagged model | 234 | 299.88 | .980 | .019 | .055 | 6.09 | .048 |
|  |  |  |  |  |  |  |  |
| **Self-Reported Parental Intimacy** |  |  |  |  |  |  |  |
| ***Time Invariance*** |  |  |  |  |  |  |  |
| Configural time invariance | 213 | 412.04 | .922 | .033 | .066 |  |  |
| Weak time invariance | 229 | 548.49 | .852 | .045 | .300 | 65.17 | < .001 |
| Strong time invariance | 241 | 604.38 | .832 | .046 | .311 | 59.97 | < .001 |
| Strict time invariance | 257 | 1413.10 | .261 | .095 | .936 | 187.75 | < .001 |
| ***Factorial Invariance Between Mothering and Fathering*** |  |  |  |  |  |  |  |
| Configural time invariance + |  |  |  |  |  |  |  |
| Configural factorial invariance | 213 | 412.04 | .922 | .033 | .066 |  |  |
| Weak factorial invariance | 225 | 460.11 | .900 | .038 | .227 | 31.55 | .002 |
| Strong factorial invariance | 234 | 483.70 | .890 | .039 | .237 | 22.94 | .006 |
| Strict factorial invariance | 246 | 604.28 | .790 | .052 | .525 | 41.44 | < .001 |
| ***Fixed Error Term Correlations of Corresponding Indicators*** |  |  |  |  |  |  |  |
| Configural time invariance + configural factorial invariance | 213 | 412.04 | .922 | .033 | .066 |  |  |
| Fixing the correlations of indicator error terms that do not weaken model fit to the same value or zero | 227 | 428.65 | .920 | .034 | .068 | 18.82 | .172 |
| ***Autoregressive Cross-Lagged Models*** |  |  |  |  |  |  |  |
| Second-order autoregressive cross-lagged model | 229 | 431.14 | .919 | .034 | .069 |  |  |
| First-order autoregressive cross-lagged model | 231 | 429.88 | .920 | .033 | .069 | 1.11 | .573 |
| **Partner-Reported Parental Intimacy** |  |  |  |  |  |  |  |
| ***Time Invariance*** |  |  |  |  |  |  |  |
| Configural time invariance | 213 | 345.97 | .967 | .030 | .070 |  |  |
| Weak time invariance | 229 | 759.44 | .846 | .060 | .639 | 159.20 | < .001 |
| Strong time invariance | 241 | 780.22 | .842 | .059 | .637 | 21.71 | .041 |
| Strict time invariance | 257 | 1109.10 | .690 | .081 | .856 | 121.81 | < .001 |
| ***Factorial Invariance Between Mothering and Fathering*** |  |  |  |  |  |  |  |
| Configural time invariance + |  |  |  |  |  |  |  |
| Configural factorial invariance | 213 | 345.97 | .967 | .030 | .070 |  |  |
| Weak factorial invariance | 225 | 400.09 | .950 | .035 | .213 | 29.83 | .003 |
| Strong factorial invariance | 234 | 426.30 | .943 | .035 | .212 | 25.96 | .002 |
| Strict factorial invariance | 246 | 499.51 | .914 | .043 | .292 | 34.93 | < .001 |
| ***Fixed Error Term Correlations of Corresponding Indicators*** |  |  |  |  |  |  |  |
| Configural time invariance + configural factorial invariance | 213 | 345.97 | .967 | .030 | .070 |  |  |
| Fixing the correlations of indicator error terms that do not weaken model fit to the same value or zero | 232 | 362.38 | .967 | .028 | .073 | 18.96 | .460 |
| ***Autoregressive Cross-Lagged Models*** |  |  |  |  |  |  |  |
| Second-order autoregressive cross-lagged model | 234 | 370.01 | .965 | .029 | .078 |  |  |
| First-order autoregressive cross-lagged model | 236 | 368.94 | .966 | .028 | .077 | 0.24 | .888 |
| *Note. N* = 885. In the scaled ∆χ^2^ tests, a model is compared to a more complex model above (e.g., a weak time invariance model is compared to a configural time invariance model). The model comparisons CFI = robust comparative fit index; RMSEA = robust root-mean-square error of approximation; SRMR = standardized root mean square residual. | | | | | | | |
|  | | | | | | | |
