# Supplementary material for: Developmental Stage-Specific Effects of Parenting on Adolescents’ Emotion Regulation: A Longitudinal Study From Infancy to Late Adolescence
Source: Front Psychol. 2021 Jun 4;12:582770. doi: 10.3389/fpsyg.2021.582770 (PMC8211896; doi:10.3389/fpsyg.2021.582770)
Supplement: Supplementary file 4 [file Table_4.docx]

**Supplementary Material 4.** Standardized Parameter Estimates for Selected Measurement Models of Parental Autonomy and Intimacy.

|  | |  |  | | |
| --- | --- | --- | --- | --- | --- |
| **Self-Reported Parental Autonomy** | |  |  | | |
|  | |  |  | | |
| ***Factor Loadings*** | | λ | | | S.E. |
| Maternal autonomy at T1 → Maternal autonomy 1 at T1 | | .326 | | | 0.051 |
| Maternal autonomy at T1 → Maternal autonomy 2 at T1 | | .698 | | | 0.045 |
| Maternal autonomy at T1 → Maternal autonomy 3 at T1 | | .594 | | | 0.050 |
| Maternal autonomy at T1 → Maternal autonomy 4 at T1 | | .683 | | | 0.049 |
| Maternal autonomy at T2 → Maternal autonomy 1 at T2 | | .180 | | | 0.055 |
| Maternal autonomy at T2 → Maternal autonomy 2 at T2 | | .675 | | | 0.053 |
| Maternal autonomy at T2 → Maternal autonomy 3 at T2 | | .654 | | | 0.062 |
| Maternal autonomy at T2 → Maternal autonomy 4 at T2 | | .788 | | | 0.059 |
| Maternal autonomy at T3 → Maternal autonomy 1 at T3 | | .295 | | | 0.048 |
| Maternal autonomy at T3 → Maternal autonomy 2 at T3 | | .576 | | | 0.049 |
| Maternal autonomy at T3 → Maternal autonomy 3 at T3 | | .593 | | | 0.056 |
| Maternal autonomy at T3 → Maternal autonomy 4 at T3 | | .790 | | | 0.057 |
| Paternal autonomy at T1 → Paternal autonomy 1 at T1 | | .264 | | | 0.052 |
| Paternal autonomy at T1 → Paternal autonomy 2 at T1 | | .803 | | | 0.033 |
| Paternal autonomy at T1 → Paternal autonomy 3 at T1 | | .671 | | | 0.043 |
| Paternal autonomy at T1 → Paternal autonomy 4 at T1 | | .754 | | | 0.062 |
| Paternal autonomy at T2 → Paternal autonomy 1 at T2 | | .127 | | | 0.060 |
| Paternal autonomy at T2 → Paternal autonomy 2 at T2 | | .724 | | | 0.083 |
| Paternal autonomy at T2 → Paternal autonomy 3 at T2 | | .738 | | | 0.052 |
| Paternal autonomy at T2 → Paternal autonomy 4 at T2 | | .779 | | | 0.071 |
| Paternal autonomy at T3 → Paternal autonomy 1 at T3 | | .131 | | | 0.060 |
| Paternal autonomy at T3 → Paternal autonomy 2 at T3 | | .532 | | | 0.077 |
| Paternal autonomy at T3 → Paternal autonomy 3 at T3 | | .790 | | | 0.070 |
| Paternal autonomy at T3 → Paternal autonomy 4 at T3 | | .574 | | | 0.079 |
|  | |  | | |  |
| ***Correlations Between Latent Variables*** | | ρ | | | S.E. |
| Maternal autonomy at T1 ↔ Maternal autonomy at T2 | | .421 | | | 0.087 |
| Maternal autonomy at T1 ↔ Maternal autonomy at T3 | | .424 | | | 0.070 |
| Maternal autonomy at T2 ↔ Maternal autonomy at T3 | | .473 | | | 0.092 |
| Maternal autonomy at T1 ↔ Paternal autonomy at T1 | | .169 | | | 0.053 |
| Maternal autonomy at T1 ↔ Paternal autonomy at T2 | | −.031 | | | 0.074 |
| Maternal autonomy at T1 ↔ Paternal autonomy at T3 | | −.107 | | | 0.069 |
| Maternal autonomy at T2 ↔ Paternal autonomy at T1 | | .092 | | | 0.058 |
| Maternal autonomy at T2 ↔ Paternal autonomy at T2 | | .092 | | | 0.063 |
| Maternal autonomy at T2 ↔ Paternal autonomy at T3 | | −.020 | | | 0.064 |
| Maternal autonomy at T3 ↔ Paternal autonomy at T1 | | −.015 | | | 0.071 |
| Maternal autonomy at T3 ↔ Paternal autonomy at T2 | | −.066 | | | 0.087 |
| Maternal autonomy at T3 ↔ Paternal autonomy at T3 | | .098 | | | 0.075 |
| Paternal autonomy at T1 ↔ Paternal autonomy at T2 | | .209 | | | 0.088 |
| Paternal autonomy at T1 ↔ Paternal autonomy at T3 | | .235 | | | 0.082 |
| Paternal autonomy at T2 ↔ Paternal autonomy at T3 | | .273 | | | 0.092 |
|  | |  | | |  |
| ***Error Term Correlations of Indicators*** | | ρ | | | S.E. |
| Maternal autonomy 1 at T1 ↔ Maternal autonomy 1 at T2 | | .338 | | | 0.059 |
| Maternal autonomy 1 at T1 ↔ Maternal autonomy 1 at T3 | | .341 | | | 0.057 |
| Maternal autonomy 1 at T2 ↔ Maternal autonomy 1 at T3 | | .320 | | | 0.061 |
| Maternal autonomy 2 at T1 ↔ Maternal autonomy 2 at T2 | | .098 | | | 0.074 |
| Maternal autonomy 2 at T1 ↔ Maternal autonomy 2 at T3 | | −.020 | | | 0.066 |
| Maternal autonomy 2 at T2 ↔ Maternal autonomy 2 at T3 | | .163 | | | 0.067 |
| Maternal autonomy 3 at T1 ↔ Maternal autonomy 3 at T2 | | .123 | | | 0.114 |
| Maternal autonomy 3 at T1 ↔ Maternal autonomy 3 at T3 | | −.028 | | | 0.050 |
| Maternal autonomy 3 at T2 ↔ Maternal autonomy 3 at T3 | | .198 | | | 0.096 |
| Maternal autonomy 4 at T1 ↔ Maternal autonomy 4 at T2 | | −.051 | | | 0.094 |
| Maternal autonomy 4 at T1 ↔ Maternal autonomy 4 at T3 | | −.007 | | | 0.094 |
| Maternal autonomy 4 at T2 ↔ Maternal autonomy 4 at T3 | | .108 | | | 0.119 |
| Paternal autonomy 1 at T1 ↔ Paternal autonomy 1 at T2 | | .541 | | | 0.071 |
| Paternal autonomy 1 at T1 ↔ Paternal autonomy 1 at T3 | | .349 | | | 0.066 |
| Paternal autonomy 1 at T2 ↔ Paternal autonomy 1 at T3 | | .527 | | | 0.062 |
| Paternal autonomy 2 at T1 ↔ Paternal autonomy 2 at T2 | | .262 | | | 0.096 |
| Paternal autonomy 2 at T1 ↔ Paternal autonomy 2 at T3 | | .020 | | | 0.080 |
| Paternal autonomy 2 at T2 ↔ Paternal autonomy 2 at T3 | | .025 | | | 0.109 |
| Paternal autonomy 3 at T1 ↔ Paternal autonomy 3 at T2 | | .258 | | | 0.101 |
| Paternal autonomy 3 at T1 ↔ Paternal autonomy 3 at T3 | | −.042 | | | 0.097 |
| Paternal autonomy 3 at T2 ↔ Paternal autonomy 3 at T3 | | .147 | | | 0.110 |
| Paternal autonomy 4 at T1 ↔ Paternal autonomy 4 at T2 | | .388 | | | 0.194 |
| Paternal autonomy 4 at T1 ↔ Paternal autonomy 4 at T3 | | .136 | | | 0.101 |
| Paternal autonomy 4 at T2 ↔ Paternal autonomy 4 at T3 | | .418 | | | 0.077 |
|  | |  | | |  |
| **Partner-Reported Parental Autonomy** |  |  | | | |
|  |  |  | | | |
| ***Factor Loadings*** | | λ | | | S.E. |
| Maternal autonomy at T1 → Maternal autonomy 1 at T1 | | .195 | | | 0.055 |
| Maternal autonomy at T1 → Maternal autonomy 2 at T1 | | .638 | | | 0.075 |
| Maternal autonomy at T1 → Maternal autonomy 3 at T1 | | .800 | | | 0.054 |
| Maternal autonomy at T1 → Maternal autonomy 4 at T1 | | .698 | | | 0.093 |
| Maternal autonomy at T2 → Maternal autonomy 1 at T2 | | .258 | | | 0.056 |
| Maternal autonomy at T2 → Maternal autonomy 2 at T2 | | .733 | | | 0.055 |
| Maternal autonomy at T2 → Maternal autonomy 3 at T2 | | .803 | | | 0.051 |
| Maternal autonomy at T2 → Maternal autonomy 4 at T2 | | .625 | | | 0.085 |
| Maternal autonomy at T3 → Maternal autonomy 1 at T3 | | .381 | | | 0.057 |
| Maternal autonomy at T3 → Maternal autonomy 2 at T3 | | .756 | | | 0.038 |
| Maternal autonomy at T3 → Maternal autonomy 3 at T3 | | .821 | | | 0.046 |
| Maternal autonomy at T3 → Maternal autonomy 4 at T3 | | .820 | | | 0.055 |
| Paternal autonomy at T1 → Paternal autonomy 1 at T1 | | .462 | | | 0.055 |
| Paternal autonomy at T1 → Paternal autonomy 2 at T1 | | .788 | | | 0.035 |
| Paternal autonomy at T1 → Paternal autonomy 3 at T1 | | .757 | | | 0.036 |
| Paternal autonomy at T1 → Paternal autonomy 4 at T1 | | .793 | | | 0.038 |
| Paternal autonomy at T2 → Paternal autonomy 1 at T2 | | .418 | | | 0.062 |
| Paternal autonomy at T2 → Paternal autonomy 2 at T2 | | .685 | | | 0.058 |
| Paternal autonomy at T2 → Paternal autonomy 3 at T2 | | .751 | | | 0.040 |
| Paternal autonomy at T2 → Paternal autonomy 4 at T2 | | .798 | | | 0.042 |
| Paternal autonomy at T3 → Paternal autonomy 1 at T3 | | .542 | | | 0.051 |
| Paternal autonomy at T3 → Paternal autonomy 2 at T3 | | .803 | | | 0.033 |
| Paternal autonomy at T3 → Paternal autonomy 3 at T3 | | .825 | | | 0.033 |
| Paternal autonomy at T3 → Paternal autonomy 4 at T3 | | .787 | | | 0.033 |
|  | |  | | |  |
| ***Correlations Between Latent Variables*** | | ρ | | | S.E. |
| Maternal autonomy at T1 ↔ Maternal autonomy at T2 | | .415 | | | 0.083 |
| Maternal autonomy at T1 ↔ Maternal autonomy at T3 | | .319 | | | 0.067 |
| Maternal autonomy at T2 ↔ Maternal autonomy at T3 | | .382 | | | 0.079 |
| Maternal autonomy at T1 ↔ Paternal autonomy at T1 | | .055 | | | 0.055 |
| Maternal autonomy at T1 ↔ Paternal autonomy at T2 | | .107 | | | 0.071 |
| Maternal autonomy at T1 ↔ Paternal autonomy at T3 | | .007 | | | 0.062 |
| Maternal autonomy at T2 ↔ Paternal autonomy at T1 | | .109 | | | 0.084 |
| Maternal autonomy at T2 ↔ Paternal autonomy at T2 | | .057 | | | 0.078 |
| Maternal autonomy at T2 ↔ Paternal autonomy at T3 | | −.006 | | | 0.077 |
| Maternal autonomy at T3 ↔ Paternal autonomy at T1 | | .016 | | | 0.063 |
| Maternal autonomy at T3 ↔ Paternal autonomy at T2 | | .014 | | | 0.074 |
| Maternal autonomy at T3 ↔ Paternal autonomy at T3 | | .004 | | | 0.071 |
| Paternal autonomy at T1 ↔ Paternal autonomy at T2 | | .263 | | | 0.065 |
| Paternal autonomy at T1 ↔ Paternal autonomy at T3 | | .151 | | | 0.065 |
| Paternal autonomy at T2 ↔ Paternal autonomy at T3 | | .381 | | | 0.073 |
|  | |  | | |  |
| ***Error Term Correlations of Indicators*** | | ρ | | | S.E. |
| Maternal autonomy 1 at T1 ↔ Maternal autonomy 1 at T2 | | .453 | | | 0.078 |
| Maternal autonomy 1 at T1 ↔ Maternal autonomy 1 at T3 | | .258 | | | 0.065 |
| Maternal autonomy 1 at T2 ↔ Maternal autonomy 1 at T3 | | .309 | | | 0.074 |
| Maternal autonomy 2 at T1 ↔ Maternal autonomy 2 at T2 | | .159 | | | 0.123 |
| Maternal autonomy 2 at T1 ↔ Maternal autonomy 2 at T3 | | .118 | | | 0.067 |
| Maternal autonomy 2 at T2 ↔ Maternal autonomy 2 at T3 | | .318 | | | 0.106 |
| Maternal autonomy 3 at T1 ↔ Maternal autonomy 3 at T2 | | −.158 | | | 0.130 |
| Maternal autonomy 3 at T1 ↔ Maternal autonomy 3 at T3 | | .138 | | | 0.137 |
| Maternal autonomy 3 at T2 ↔ Maternal autonomy 3 at T3 | | .256 | | | 0.097 |
| Maternal autonomy 4 at T1 ↔ Maternal autonomy 4 at T2 | | −.004 | | | 0.101 |
| Maternal autonomy 4 at T1 ↔ Maternal autonomy 4 at T3 | | −.162 | | | 0.078 |
| Maternal autonomy 4 at T2 ↔ Maternal autonomy 4 at T3 | | .102 | | | 0.136 |
| Paternal autonomy 1 at T1 ↔ Paternal autonomy 1 at T2 | | .145 | | | 0.059 |
| Paternal autonomy 1 at T1 ↔ Paternal autonomy 1 at T3 | | .185 | | | 0.063 |
| Paternal autonomy 1 at T2 ↔ Paternal autonomy 1 at T3 | | .194 | | | 0.067 |
| Paternal autonomy 2 at T1 ↔ Paternal autonomy 2 at T2 | | −.007 | | | 0.093 |
| Paternal autonomy 2 at T1 ↔ Paternal autonomy 2 at T3 | | .232 | | | 0.085 |
| Paternal autonomy 2 at T2 ↔ Paternal autonomy 2 at T3 | | .113 | | | 0.088 |
| Paternal autonomy 3 at T1 ↔ Paternal autonomy 3 at T2 | | .136 | | | 0.075 |
| Paternal autonomy 3 at T1 ↔ Paternal autonomy 3 at T3 | | .061 | | | 0.105 |
| Paternal autonomy 3 at T2 ↔ Paternal autonomy 3 at T3 | | .378 | | | 0.093 |
| Paternal autonomy 4 at T1 ↔ Paternal autonomy 4 at T2 | | .000 | | | 0.089 |
| Paternal autonomy 4 at T1 ↔ Paternal autonomy 4 at T3 | | .017 | | | 0.073 |
| Paternal autonomy 4 at T2 ↔ Paternal autonomy 4 at T3 | | −.053 | | | 0.098 |
|  | |  | | |  |
| **Self-Reported Parental Intimacy** |  |  | | | |
|  |  |  | | | |
| ***Factor Loadings*** | | λ | | | S.E. |
| Maternal intimacy at T1 → Maternal intimacy 1 at T1 | | .606 | | | 0.073 |
| Maternal intimacy at T1 → Maternal intimacy 2 at T1 | | .764 | | | 0.067 |
| Maternal intimacy at T1 → Maternal intimacy 3 at T1 | | .564 | | | 0.069 |
| Maternal intimacy at T1 → Maternal intimacy 4 at T1 | | .689 | | | 0.094 |
| Maternal intimacy at T2 → Maternal intimacy 1 at T2 | | .497 | | | 0.086 |
| Maternal intimacy at T2 → Maternal intimacy 2 at T2 | | .858 | | | 0.075 |
| Maternal intimacy at T2 → Maternal intimacy 3 at T2 | | .396 | | | 0.091 |
| Maternal intimacy at T2 → Maternal intimacy 4 at T2 | | .809 | | | 0.036 |
| Maternal intimacy at T3 → Maternal intimacy 1 at T3 | | .363 | | | 0.106 |
| Maternal intimacy at T3 → Maternal intimacy 2 at T3 | | .595 | | | 0.092 |
| Maternal intimacy at T3 → Maternal intimacy 3 at T3 | | .439 | | | 0.081 |
| Maternal intimacy at T3 → Maternal intimacy 4 at T3 | | .720 | | | 0.086 |
| Paternal intimacy at T1 → Paternal intimacy 1 at T1 | | .639 | | | 0.085 |
| Paternal intimacy at T1 → Paternal intimacy 2 at T1 | | .764 | | | 0.061 |
| Paternal intimacy at T1 → Paternal intimacy 3 at T1 | | .665 | | | 0.069 |
| Paternal intimacy at T1 → Paternal intimacy 4 at T1 | | .728 | | | 0.083 |
| Paternal intimacy at T2 → Paternal intimacy 1 at T2 | | .517 | | | 0.118 |
| Paternal intimacy at T2 → Paternal intimacy 2 at T2 | | .693 | | | 0.102 |
| Paternal intimacy at T2 → Paternal intimacy 3 at T2 | | .512 | | | 0.086 |
| Paternal intimacy at T2 → Paternal intimacy 4 at T2 | | .703 | | | 0.125 |
| Paternal intimacy at T3 → Paternal intimacy 1 at T3 | | .605 | | | 0.080 |
| Paternal intimacy at T3 → Paternal intimacy 2 at T3 | | .607 | | | 0.074 |
| Paternal intimacy at T3 → Paternal intimacy 3 at T3 | | .613 | | | 0.076 |
| Paternal intimacy at T3 → Paternal intimacy 4 at T3 | | .835 | | | 0.065 |
|  | |  | | |  |
| ***Correlations Between Latent Variables*** | | ρ | | | S.E. |
| Maternal intimacy at T1 ↔ Maternal intimacy at T2 | | .434 | | | 0.099 |
| Maternal intimacy at T1 ↔ Maternal intimacy at T3 | | .274 | | | 0.118 |
| Maternal intimacy at T2 ↔ Maternal intimacy at T3 | | .408 | | | 0.085 |
| Maternal intimacy at T1 ↔ Paternal intimacy at T1 | | .051 | | | 0.049 |
| Maternal intimacy at T1 ↔ Paternal intimacy at T2 | | .027 | | | 0.061 |
| Maternal intimacy at T1 ↔ Paternal intimacy at T3 | | −.003 | | | 0.047 |
| Maternal intimacy at T2 ↔ Paternal intimacy at T1 | | .045 | | | 0.055 |
| Maternal intimacy at T2 ↔ Paternal intimacy at T2 | | .057 | | | 0.088 |
| Maternal intimacy at T2 ↔ Paternal intimacy at T3 | | .063 | | | 0.072 |
| Maternal intimacy at T3 ↔ Paternal intimacy at T1 | | −.025 | | | 0.080 |
| Maternal intimacy at T3 ↔ Paternal intimacy at T2 | | .207 | | | 0.104 |
| Maternal intimacy at T3 ↔ Paternal intimacy at T3 | | .114 | | | 0.085 |
| Paternal intimacy at T1 ↔ Paternal intimacy at T2 | | .364 | | | 0.099 |
| Paternal intimacy at T1 ↔ Paternal intimacy at T3 | | .089 | | | 0.080 |
| Paternal intimacy at T2 ↔ Paternal intimacy at T3 | | .155 | | | 0.076 |
|  | |  | | |  |
| ***Error Term Correlations of Indicators*** | | ρ | | | S.E. |
| Maternal intimacy 1 at T1 ↔ Maternal intimacy 1 at T2 | | .227 | | | 0.055 |
| Maternal intimacy 1 at T1 ↔ Maternal intimacy 1 at T3 | | .180 | | | 0.101 |
| Maternal intimacy 1 at T2 ↔ Maternal intimacy 1 at T3 | | .094 | | | 0.043 |
| Maternal intimacy 2 at T1 ↔ Maternal intimacy 2 at T2 | | −.132 | | | 0.137 |
| Maternal intimacy 2 at T1 ↔ Maternal intimacy 2 at T3 | | −.044 | | | 0.090 |
| Maternal intimacy 2 at T2 ↔ Maternal intimacy 2 at T3 | | .076 | | | 0.131 |
| Maternal intimacy 3 at T1 ↔ Maternal intimacy 3 at T2 | | .270 | | | 0.058 |
| Maternal intimacy 3 at T1 ↔ Maternal intimacy 3 at T3 | | .075 | | | 0.051 |
| Maternal intimacy 3 at T2 ↔ Maternal intimacy 3 at T3 | | .089 | | | 0.055 |
| Maternal intimacy 4 at T1 ↔ Maternal intimacy 4 at T2 | | .115 | | | 0.195 |
| Maternal intimacy 4 at T1 ↔ Maternal intimacy 4 at T3 | | .027 | | | 0.074 |
| Maternal intimacy 4 at T2 ↔ Maternal intimacy 4 at T3 | | −.012 | | | 0.106 |
| Paternal intimacy 1 at T1 ↔ Paternal intimacy 1 at T2 | | .178 | | | 0.080 |
| Paternal intimacy 1 at T1 ↔ Paternal intimacy 1 at T3 | | .078 | | | 0.083 |
| Paternal intimacy 1 at T2 ↔ Paternal intimacy 1 at T3 | | .491 | | | 0.124 |
| Paternal intimacy 2 at T1 ↔ Paternal intimacy 2 at T2 | | −.066 | | | 0.097 |
| Paternal intimacy 2 at T1 ↔ Paternal intimacy 2 at T3 | | −.223 | | | 0.118 |
| Paternal intimacy 2 at T2 ↔ Paternal intimacy 2 at T3 | | .288 | | | 0.126 |
| Paternal intimacy 3 at T1 ↔ Paternal intimacy 3 at T2 | | .265 | | | 0.087 |
| Paternal intimacy 3 at T1 ↔ Paternal intimacy 3 at T3 | | .109 | | | 0.073 |
| Paternal intimacy 3 at T2 ↔ Paternal intimacy 3 at T3 | | .319 | | | 0.087 |
| Paternal intimacy 4 at T1 ↔ Paternal intimacy 4 at T2 | | .181 | | | 0.091 |
| Paternal intimacy 4 at T1 ↔ Paternal intimacy 4 at T3 | | .287 | | | 0.116 |
| Paternal intimacy 4 at T2 ↔ Paternal intimacy 4 at T3 | | .276 | | | 0.191 |
|  | |  | | |  |
| **Partner-Reported Parental Intimacy** |  | | |  | |
|  |  | | |  | |
| ***Factor Loadings*** | | λ | | | S.E. |
| Maternal intimacy at T1 → Maternal intimacy 1 at T1 | | .842 | | | 0.086 |
| Maternal intimacy at T1 → Maternal intimacy 2 at T1 | | .392 | | | 0.127 |
| Maternal intimacy at T1 → Maternal intimacy 3 at T1 | | .319 | | | 0.121 |
| Maternal intimacy at T1 → Maternal intimacy 4 at T1 | | .855 | | | 0.069 |
| Maternal intimacy at T2 → Maternal intimacy 1 at T2 | | .738 | | | 0.102 |
| Maternal intimacy at T2 → Maternal intimacy 2 at T2 | | .846 | | | 0.084 |
| Maternal intimacy at T2 → Maternal intimacy 3 at T2 | | .584 | | | 0.073 |
| Maternal intimacy at T2 → Maternal intimacy 4 at T2 | | .783 | | | 0.088 |
| Maternal intimacy at T3 → Maternal intimacy 1 at T3 | | .816 | | | 0.037 |
| Maternal intimacy at T3 → Maternal intimacy 2 at T3 | | .758 | | | 0.066 |
| Maternal intimacy at T3 → Maternal intimacy 3 at T3 | | .619 | | | 0.066 |
| Maternal intimacy at T3 → Maternal intimacy 4 at T3 | | .923 | | | 0.024 |
| Paternal intimacy at T1 → Paternal intimacy 1 at T1 | | .752 | | | 0.044 |
| Paternal intimacy at T1 → Paternal intimacy 2 at T1 | | .826 | | | 0.056 |
| Paternal intimacy at T1 → Paternal intimacy 3 at T1 | | .699 | | | 0.049 |
| Paternal intimacy at T1 → Paternal intimacy 4 at T1 | | .818 | | | 0.044 |
| Paternal intimacy at T2 → Paternal intimacy 1 at T2 | | .714 | | | 0.052 |
| Paternal intimacy at T2 → Paternal intimacy 2 at T2 | | .837 | | | 0.037 |
| Paternal intimacy at T2 → Paternal intimacy 3 at T2 | | .558 | | | 0.055 |
| Paternal intimacy at T2 → Paternal intimacy 4 at T2 | | .825 | | | 0.048 |
| Paternal intimacy at T3 → Paternal intimacy 1 at T3 | | .746 | | | 0.035 |
| Paternal intimacy at T3 → Paternal intimacy 2 at T3 | | .909 | | | 0.018 |
| Paternal intimacy at T3 → Paternal intimacy 3 at T3 | | .716 | | | 0.034 |
| Paternal intimacy at T3 → Paternal intimacy 4 at T3 | | .886 | | | 0.023 |
|  | |  | | |  |
| ***Correlations Between Latent Variables*** | | ρ | | | S.E. |
| Maternal intimacy at T1 ↔ Maternal intimacy at T2 | | .484 | | | 0.178 |
| Maternal intimacy at T1 ↔ Maternal intimacy at T3 | | .059 | | | 0.156 |
| Maternal intimacy at T2 ↔ Maternal intimacy at T3 | | .185 | | | 0.091 |
| Maternal intimacy at T1 ↔ Paternal intimacy at T1 | | .124 | | | 0.115 |
| Maternal intimacy at T1 ↔ Paternal intimacy at T2 | | .102 | | | 0.092 |
| Maternal intimacy at T1 ↔ Paternal intimacy at T3 | | −.037 | | | 0.094 |
| Maternal intimacy at T2 ↔ Paternal intimacy at T1 | | −.011 | | | 0.079 |
| Maternal intimacy at T2 ↔ Paternal intimacy at T2 | | .095 | | | 0.101 |
| Maternal intimacy at T2 ↔ Paternal intimacy at T3 | | .024 | | | 0.095 |
| Maternal intimacy at T3 ↔ Paternal intimacy at T1 | | .107 | | | 0.071 |
| Maternal intimacy at T3 ↔ Paternal intimacy at T2 | | −.094 | | | 0.077 |
| Maternal intimacy at T3 ↔ Paternal intimacy at T3 | | .092 | | | 0.084 |
| Paternal intimacy at T1 ↔ Paternal intimacy at T2 | | .438 | | | 0.082 |
| Paternal intimacy at T1 ↔ Paternal intimacy at T3 | | .212 | | | 0.068 |
| Paternal intimacy at T2 ↔ Paternal intimacy at T3 | | .405 | | | 0.084 |
|  | |  | | |  |
| ***Error Term Correlations of Indicators*** | | ρ | | | S.E. |
| Maternal intimacy 1 at T1 ↔ Maternal intimacy 1 at T2 | | .017 | | | 0.140 |
| Maternal intimacy 1 at T1 ↔ Maternal intimacy 1 at T3 | | −.006 | | | 0.101 |
| Maternal intimacy 1 at T2 ↔ Maternal intimacy 1 at T3 | | .135 | | | 0.077 |
| Maternal intimacy 2 at T1 ↔ Maternal intimacy 2 at T2 | | .371 | | | 0.157 |
| Maternal intimacy 2 at T1 ↔ Maternal intimacy 2 at T3 | | −.026 | | | 0.147 |
| Maternal intimacy 2 at T2 ↔ Maternal intimacy 2 at T3 | | .403 | | | 0.146 |
| Maternal intimacy 3 at T1 ↔ Maternal intimacy 3 at T2 | | .235 | | | 0.086 |
| Maternal intimacy 3 at T1 ↔ Maternal intimacy 3 at T3 | | .016 | | | 0.061 |
| Maternal intimacy 3 at T2 ↔ Maternal intimacy 3 at T3 | | .152 | | | 0.077 |
| Maternal intimacy 4 at T1 ↔ Maternal intimacy 4 at T2 | | .078 | | | 0.181 |
| Maternal intimacy 4 at T1 ↔ Maternal intimacy 4 at T3 | | −.212 | | | 0.168 |
| Maternal intimacy 4 at T2 ↔ Maternal intimacy 4 at T3 | | .233 | | | 0.140 |
| Paternal intimacy 1 at T1 ↔ Paternal intimacy 1 at T2 | | .127 | | | 0.100 |
| Paternal intimacy 1 at T1 ↔ Paternal intimacy 1 at T3 | | .130 | | | 0.091 |
| Paternal intimacy 1 at T2 ↔ Paternal intimacy 1 at T3 | | .241 | | | 0.081 |
| Paternal intimacy 2 at T1 ↔ Paternal intimacy 2 at T2 | | .044 | | | 0.077 |
| Paternal intimacy 2 at T1 ↔ Paternal intimacy 2 at T3 | | .140 | | | 0.148 |
| Paternal intimacy 2 at T2 ↔ Paternal intimacy 2 at T3 | | −.093 | | | 0.111 |
| Paternal intimacy 3 at T1 ↔ Paternal intimacy 3 at T2 | | .331 | | | 0.074 |
| Paternal intimacy 3 at T1 ↔ Paternal intimacy 3 at T3 | | .263 | | | 0.071 |
| Paternal intimacy 3 at T2 ↔ Paternal intimacy 3 at T3 | | .315 | | | 0.076 |
| Paternal intimacy 4 at T1 ↔ Paternal intimacy 4 at T2 | | .006 | | | 0.076 |
| Paternal intimacy 4 at T1 ↔ Paternal intimacy 4 at T3 | | −.038 | | | 0.122 |
| Paternal intimacy 4 at T2 ↔ Paternal intimacy 4 at T3 | | −.086 | | | 0.103 |
| *Note.* T1 = infancy; T2 = middle childhood; T3 = late adolescence. | | | | | |
|  | | | | | |
