# Supplementary material for: Developmental Stage-Specific Effects of Parenting on Adolescents’ Emotion Regulation: A Longitudinal Study From Infancy to Late Adolescence
Source: Front Psychol. 2021 Jun 4;12:582770. doi: 10.3389/fpsyg.2021.582770 (PMC8211896; doi:10.3389/fpsyg.2021.582770)
Supplement: Supplementary file 5 [file Table_5.docx]

**Supplementary Material 5.** Standardized Parameter Estimates for Selected Measurement Model of Adolescents’ Emotion Regulation Patterns.

|  |  | |  |
| --- | --- | --- | --- |
| **Factor Loadings of Reappraisal** | λ | | S.E. |
| Reappraisal 1 | .704 | | 0.040 |
| Reappraisal 2 | .715 | | 0.038 |
| Reappraisal 3 | .389 | | 0.052 |
| Reappraisal 4 | .825 | | 0.024 |
| Reappraisal 5 | .757 | | 0.029 |
| Reappraisal 6 | .834 | | 0.023 |
| **Factor Loadings of Suppression** | λ | | S.E. |
| Suppression 1 | .734 | | 0.035 |
| Suppression 2 | .600 | | 0.040 |
| Suppression 3 | .791 | | 0.030 |
| Suppression 4 | .627 | | 0.041 |
| **Factor Loadings of Rumination** | λ | | S.E. |
| Rumination 1 | .498 | | 0.042 |
| Rumination 2 | .630 | | 0.034 |
| Catastrophizing 1 | .627 | | 0.039 |
| Catastrophizing 2 | .872 | | 0.019 |
| Catastrophizing 3 | .687 | | 0.036 |
| Catastrophizing 4 | .809 | | 0.023 |
| **Correlations Between Latent Emotion Regulation Patterns** | ρ | | S.E. |
| Reappraisal ↔ Suppression | −.076 | | 0.064 |
| Reappraisal ↔ Rumination | −.190 | | 0.059 |
| Suppression ↔ Rumination | .152 | | 0.062 |
|  | |  |  |
