# Supplementary material for: Developmental Stage-Specific Effects of Parenting on Adolescents’ Emotion Regulation: A Longitudinal Study From Infancy to Late Adolescence
Source: Front Psychol. 2021 Jun 4;12:582770. doi: 10.3389/fpsyg.2021.582770 (PMC8211896; doi:10.3389/fpsyg.2021.582770)
Supplement: Supplementary file 6 [file Table_6.docx]

**Supplementary Material 6.** Standardized Parameter Estimates for Final Selected Models of Parental Autonomy and Intimacy with No Effects of Parenting on Adolescents’ Emotion Regulation Patterns.

|  | |  |  | | | |
| --- | --- | --- | --- | --- | --- | --- |
| **Self-Reported Parental Autonomy** | |  |  | | | |
|  | |  |  | | | |
| ***Path Coefficients*** | | | | β | | S.E. |
| Maternal autonomy at T1 → Maternal autonomy at T2 | | | | 0.428 | | 0.085 |
| Paternal autonomy at T1 → Maternal autonomy at T2 | | | | 0.008 | | 0.060 |
| Maternal autonomy at T2 → Maternal autonomy at T3 | | | | 0.383 | | 0.125 |
| Paternal autonomy at T2 → Maternal autonomy at T3 | | | | −0.088 | | 0.081 |
| Maternal autonomy at T1 → Maternal autonomy at T3 | | | | 0.240 | | 0.118 |
|  | | | |  | |  |
| Paternal autonomy at T1 → Paternal autonomy at T2 | | | | 0.223 | | 0.088 |
| Maternal autonomy at T1 → Paternal autonomy at T2 | | | | −0.070 | | 0.076 |
| Paternal autonomy at T2 → Paternal autonomy at T3 | | | | 0.295 | | 0.087 |
| Maternal autonomy at T1 → Paternal autonomy at T3 | | | | −0.110 | | 0.066 |
| Paternal autonomy at T1 → Paternal autonomy at T3 | | | | 0.182 | | 0.074 |
|  | | | |  | |  |
| Parents’ average education → Maternal autonomy at T2 | | | | −0.142 | | 0.047 |
| Child’s sex → Maternal autonomy at T2 | | | | 0.110 | | 0.048 |
| Assisted reproductive treatment → Maternal autonomy at T2 | | | | 0.048 | | 0.045 |
| Parents’ average education → Maternal autonomy at T3 | | | | −0.030 | | 0.056 |
| Child’s sex → Maternal autonomy at T3 | | | | 0.036 | | 0.056 |
| Assisted reproductive treatment → Maternal autonomy at T3 | | | | 0.017 | | 0.050 |
|  | | | |  | |  |
| Parents’ average education → Paternal autonomy at T2 | | | | 0.027 | | 0.062 |
| Child’s sex → Paternal autonomy at T2 | | | | −0.115 | | 0.056 |
| Assisted reproductive treatment → Paternal autonomy at T2 | | | | 0.015 | | 0.055 |
| Parents’ average education → Paternal autonomy at T3 | | | | −0.154 | | 0.061 |
| Child’s sex → Paternal autonomy at T3 | | | | 0.103 | | 0.057 |
| Assisted reproductive treatment → Paternal autonomy at T3 | | | | 0.020 | | 0.058 |
|  | | | |  | |  |
| Parents’ average education → Adolescents’ reappraisal | | | | 0.097 | | 0.053 |
| Child’s sex → Adolescents’ reappraisal | | | | 0.086 | | 0.049 |
| Assisted reproductive treatment → Adolescents’ reappraisal | | | | 0.000 | | 0.050 |
| Parents’ average education → Adolescents’ suppression | | | | −0.050 | | 0.057 |
| Child’s sex → Adolescents’ suppression | | | | −0.283 | | 0.048 |
| Assisted reproductive treatment → Adolescents’ suppression | | | | 0.041 | | 0.051 |
| Parents’ average education → Adolescents’ rumination | | | | −0.033 | | 0.054 |
| Child’s sex → Adolescents’ rumination | | | | 0.118 | | 0.049 |
| Assisted reproductive treatment → Adolescents’ rumination | | | | 0.070 | | 0.048 |
|  | | | |  | |  |
| ***Correlations of Latent Variables and Covariates*** | | | | ρ | | S.E. |
| Maternal autonomy at T1 ↔ Paternal autonomy at T1 | | | | .163 | | 0.052 |
| Maternal autonomy at T2 ↔ Paternal autonomy at T2 | | | | .147 | | 0.070 |
| Maternal autonomy at T3 ↔ Paternal autonomy at T3 | | | | .200 | | 0.083 |
| Reappraisal ↔ Suppression | | | | −.023 | | 0.066 |
| Reappraisal ↔ Rumination | | | | −.172 | | 0.059 |
| Rumination ↔ Suppression | | | | .175 | | 0.062 |
|  | | | |  | |  |
| Parents’ average education ↔ Maternal autonomy at T1 | | | | −.057 | | 0.049 |
| Child’s sex ↔ Maternal autonomy at T1 | | | | −.004 | | 0.049 |
| Assisted reproductive treatment ↔ Maternal autonomy at T1 | | | | −.054 | | 0.047 |
| Parents’ average education ↔ Paternal autonomy at T1 | | | | −.073 | | 0.047 |
| Child’s sex ↔ Paternal autonomy at T1 | | | | .022 | | 0.049 |
| Assisted reproductive treatment ↔ Paternal autonomy at T1 | | | | −.032 | | 0.047 |
| Parents’ average education ↔ Child’s sex | | | | .063 | | 0.036 |
| Assisted reproductive treatment ↔ Child’s sex | | | | .033 | | 0.035 |
| Parents’ average education ↔ Assisted reproductive treatment | | | | −.067 | | 0.035 |
|  | | | |  | |  |
| ***Factor Loadings*** | | | | λ | | S.E. |
| Maternal autonomy at T1 → Maternal autonomy 1 at T1 | | | | .324 | | 0.051 |
| Maternal autonomy at T1 → Maternal autonomy 2 at T1 | | | | .711 | | 0.046 |
| Maternal autonomy at T1 → Maternal autonomy 3 at T1 | | | | .594 | | 0.052 |
| Maternal autonomy at T1 → Maternal autonomy 4 at T1 | | | | .670 | | 0.050 |
| Maternal autonomy at T2 → Maternal autonomy 1 at T2 | | | | .180 | | 0.055 |
| Maternal autonomy at T2 → Maternal autonomy 2 at T2 | | | | .676 | | 0.051 |
| Maternal autonomy at T2 → Maternal autonomy 3 at T2 | | | | .662 | | 0.058 |
| Maternal autonomy at T2 → Maternal autonomy 4 at T2 | | | | .787 | | 0.057 |
| Maternal autonomy at T3 → Maternal autonomy 1 at T3 | | | | .292 | | 0.047 |
| Maternal autonomy at T3 → Maternal autonomy 2 at T3 | | | | .568 | | 0.049 |
| Maternal autonomy at T3 → Maternal autonomy 3 at T3 | | | | .585 | | 0.055 |
| Maternal autonomy at T3 → Maternal autonomy 4 at T3 | | | | .802 | | 0.058 |
|  | | | |  | |  |
| Paternal autonomy at T1 → Paternal autonomy 1 at T1 | | | | .259 | | 0.052 |
| Paternal autonomy at T1 → Paternal autonomy 2 at T1 | | | | .802 | | 0.034 |
| Paternal autonomy at T1 → Paternal autonomy 3 at T1 | | | | .672 | | 0.042 |
| Paternal autonomy at T1 → Paternal autonomy 4 at T1 | | | | .751 | | 0.059 |
| Paternal autonomy at T2 → Paternal autonomy 1 at T2 | | | | .127 | | 0.063 |
| Paternal autonomy at T2 → Paternal autonomy 2 at T2 | | | | .719 | | 0.084 |
| Paternal autonomy at T2 → Paternal autonomy 3 at T2 | | | | .741 | | 0.055 |
| Paternal autonomy at T2 → Paternal autonomy 4 at T2 | | | | .788 | | 0.070 |
| Paternal autonomy at T3 → Paternal autonomy 1 at T3 | | | | .148 | | 0.060 |
| Paternal autonomy at T3 → Paternal autonomy 2 at T3 | | | | .531 | | 0.073 |
| Paternal autonomy at T3 → Paternal autonomy 3 at T3 | | | | .785 | | 0.069 |
| Paternal autonomy at T3 → Paternal autonomy 4 at T3 | | | | .592 | | 0.069 |
|  | | | |  | |  |
| Reappraisal → Reappraisal factor score | | | | .942 | | .004 |
| Suppression → Suppression factor score | | | | .894 | | .006 |
| Rumination → Rumination factor score | | | | .938 | | .005 |
|  | | | |  | |  |
| ***Error Term Correlations of Indicators*** | | | | ρ | | S.E. |
| Maternal autonomy 1 at T1 ↔Maternal autonomy 1 at T2 | | | | .320 | | 0.047 |
| Maternal autonomy 1 at T1 ↔ Maternal autonomy 1 at T3 | | | | .361 | | 0.050 |
| Maternal autonomy 1 at T2 ↔ Maternal autonomy 1 at T3 | | | | .310 | | 0.046 |
| Maternal autonomy 2 at T2 ↔ Maternal autonomy 2 at T3 | | | | .170 | | 0.070 |
| Maternal autonomy 3 at T2 ↔ Maternal autonomy 3 at T3 | | | | .206 | | 0.094 |
|  | | | |  | |  |
| Paternal autonomy 1 at T1 ↔ Paternal autonomy 1 at T2 | | | | .495 | | 0.053 |
| Paternal autonomy 1 at T1 ↔ Paternal autonomy 1 at T3 | | | | .452 | | 0.047 |
| Paternal autonomy 1 at T2 ↔ Paternal autonomy 1 at T3 | | | | .482 | | 0.049 |
| Paternal autonomy 2 at T1 ↔ Paternal autonomy 2 at T2 | | | | .243 | | 0.100 |
| Paternal autonomy 3 at T1 ↔ Paternal autonomy 3 at T2 | | | | .254 | | 0.102 |
| Paternal autonomy 4 at T1 ↔ Paternal autonomy 4 at T2 | | | | .418 | | 0.107 |
| Paternal autonomy 4 at T2 ↔ Paternal autonomy 4 at T3 | | | | .303 | | 0.103 |
|  | | | |  | |  |
| **Partner-Reported Parental Autonomy** | | | |  | | |
|  | | | |  | | |
| ***Path Coefficients*** | | | | β | | S.E. |
| Maternal autonomy at T1 → Maternal autonomy at T2 | | | | 0.429 | | 0.083 |
| Paternal autonomy at T1 → Maternal autonomy at T2 | | | | 0.082 | | 0.081 |
| Maternal autonomy at T2 → Maternal autonomy at T3 | | | | 0.320 | | 0.094 |
| Paternal autonomy at T2 → Maternal autonomy at T3 | | | | −0.046 | | 0.071 |
| Maternal autonomy at T1 → Maternal autonomy at T3 | | | | 0.172 | | 0.084 |
|  | | | |  | |  |
| Paternal autonomy at T1 → Paternal autonomy at T2 | | | | 0.263 | | 0.066 |
| Maternal autonomy at T1 → Paternal autonomy at T2 | | | | 0.090 | | 0.075 |
| Paternal autonomy at T2 → Paternal autonomy at T3 | | | | 0.377 | | 0.075 |
| Maternal autonomy at T1 → Paternal autonomy at T3 | | | | −0.044 | | 0.069 |
| Paternal autonomy at T2 → Paternal autonomy at T3 | | | | 0.058 | | 0.068 |
|  | | | |  | |  |
| Parents’ average education → Maternal autonomy at T2 | | | | 0.104 | | 0.069 |
| Child’s sex → Maternal autonomy at T2 | | | | −0.074 | | 0.058 |
| Assisted reproductive treatment → Maternal autonomy at T2 | | | | 0.041 | | 0.056 |
| Parents’ average education → Maternal autonomy at T3 | | | | −0.121 | | 0.052 |
| Child’s sex → Maternal autonomy at T3 | | | | 0.080 | | 0.050 |
| Assisted reproductive treatment → Maternal autonomy at T3 | | | | 0.017 | | 0.049 |
|  | | | |  | |  |
| Parents’ average education → Paternal autonomy at T2 | | | | −0.029 | | 0.052 |
| Child’s sex → Paternal autonomy at T2 | | | | −0.007 | | 0.047 |
| Assisted reproductive treatment → Paternal autonomy at T2 | | | | −0.019 | | 0.045 |
| Parents’ average education → Paternal autonomy at T3 | | | | 0.076 | | 0.047 |
| Child’s sex → Paternal autonomy at T3 | | | | 0.054 | | 0.044 |
| Assisted reproductive treatment → Paternal autonomy at T3 | | | | −0.038 | | 0.044 |
|  | | | |  | |  |
| Parents’ average education → Adolescents’ reappraisal | | | | 0.100 | | 0.051 |
| Child’s sex → Adolescents’ reappraisal | | | | 0.085 | | 0.049 |
| Assisted reproductive treatment → Adolescents’ reappraisal | | | | −0.021 | | 0.049 |
| Parents’ average education → Adolescents’ suppression | | | | −0.045 | | 0.056 |
| Child’s sex → Adolescents’ suppression | | | | −0.279 | | 0.049 |
| Assisted reproductive treatment → Adolescents’ suppression | | | | 0.041 | | 0.050 |
| Parents’ average education → Adolescents’ rumination | | | | −0.043 | | 0.052 |
| Child’s sex → Adolescents’ rumination | | | | 0.114 | | 0.049 |
| Assisted reproductive treatment → Adolescents’ rumination | | | | 0.097 | | 0.047 |
|  | | | |  | |  |
| ***Correlations of Latent Variables and Covariates*** | | | | ρ | | S.E. |
| Maternal autonomy at T1 ↔ Paternal autonomy at T1 | | | | .056 | | 0.055 |
| Maternal autonomy at T2 ↔ Paternal autonomy at T2 | | | | .001 | | 0.099 |
| Maternal autonomy at T3 ↔ Paternal autonomy at T3 | | | | .040 | | 0.085 |
| Reappraisal ↔ Suppression | | | | −.018 | | 0.066 |
| Rumination ↔ Suppression | | | | .165 | | 0.061 |
| Reappraisal ↔ Rumination | | | | −.162 | | 0.058 |
|  | | | |  | |  |
| Parents’ average education ↔ Maternal autonomy at T1 | | | | −.159 | | 0.043 |
| Child’s sex ↔ Maternal autonomy at T1 | | | | .063 | | 0.049 |
| Assisted reproductive treatment ↔ Maternal autonomy at T1 | | | | .015 | | 0.048 |
| Parents’ average education ↔ Paternal autonomy at T1 | | | | .057 | | 0.046 |
| Child’s sex ↔ Paternal autonomy at T1 | | | | −.027 | | 0.043 |
| Assisted reproductive treatment ↔ Paternal autonomy at T1 | | | | −.024 | | 0.043 |
| Parents’ average education ↔ Child’s sex | | | | .068 | | 0.036 |
| Assisted reproductive treatment ↔ Child’s sex | | | | .029 | | 0.035 |
| Parents’ average education ↔ Assisted reproductive treatment | | | | −.062 | | 0.035 |
|  | | | |  | |  |
| ***Factor Loadings*** | | | | λ | | S.E. |
| Maternal autonomy at T1 → Maternal autonomy 1 at T1 | | | | .186 | | 0.055 |
| Maternal autonomy at T1 → Maternal autonomy 2 at T1 | | | | .641 | | 0.077 |
| Maternal autonomy at T1 → Maternal autonomy 3 at T1 | | | | .801 | | 0.051 |
| Maternal autonomy at T1 → Maternal autonomy 4 at T1 | | | | .694 | | 0.090 |
| Maternal autonomy at T2 → Maternal autonomy 1 at T2 | | | | .266 | | 0.057 |
| Maternal autonomy at T2 → Maternal autonomy 2 at T2 | | | | .735 | | 0.056 |
| Maternal autonomy at T2 → Maternal autonomy 3 at T2 | | | | .787 | | 0.050 |
| Maternal autonomy at T2 → Maternal autonomy 4 at T2 | | | | .622 | | 0.090 |
| Maternal autonomy at T3 → Maternal autonomy 1 at T3 | | | | .384 | | 0.057 |
| Maternal autonomy at T3 → Maternal autonomy 2 at T3 | | | | .755 | | 0.037 |
| Maternal autonomy at T3 → Maternal autonomy 3 at T3 | | | | .819 | | 0.045 |
| Maternal autonomy at T3 → Maternal autonomy 4 at T3 | | | | .822 | | 0.052 |
|  | | | |  | |  |
| Paternal autonomy at T1 → Paternal autonomy 1 at T1 | | | | .464 | | 0.055 |
| Paternal autonomy at T1 → Paternal autonomy 2 at T1 | | | | .788 | | 0.035 |
| Paternal autonomy at T1 → Paternal autonomy 3 at T1 | | | | .756 | | 0.036 |
| Paternal autonomy at T1 → Paternal autonomy 4 at T1 | | | | .794 | | 0.038 |
| Paternal autonomy at T2 → Paternal autonomy 1 at T2 | | | | .416 | | 0.061 |
| Paternal autonomy at T2 → Paternal autonomy 2 at T2 | | | | .688 | | 0.057 |
| Paternal autonomy at T2 → Paternal autonomy 3 at T2 | | | | .760 | | 0.039 |
| Paternal autonomy at T2 → Paternal autonomy 4 at T2 | | | | .791 | | 0.041 |
| Paternal autonomy at T3 → Paternal autonomy 1 at T3 | | | | .542 | | 0.052 |
| Paternal autonomy at T3 → Paternal autonomy 2 at T3 | | | | .808 | | 0.031 |
| Paternal autonomy at T3 → Paternal autonomy 3 at T3 | | | | .826 | | 0.031 |
| Paternal autonomy at T3 → Paternal autonomy 4 at T3 | | | | .787 | | 0.032 |
|  | | | |  | |  |
| Reappraisal → Reappraisal factor score | | | | .942 | | .004 |
| Suppression → Suppression factor score | | | | .893 | | .006 |
| Rumination → Rumination factor score | | | | .938 | | .005 |
|  | | | |  | |  |
| ***Error Term Correlations of Indicators*** | | | | ρ | | S.E. |
| Maternal autonomy 1 at T1 ↔ Maternal autonomy 1 at T2 | | | | .459 | | 0.076 |
| Maternal autonomy 1 at T1 ↔ Maternal autonomy 1 at T3 | | | | .266 | | 0.050 |
| Maternal autonomy 1 at T2 ↔ Maternal autonomy 1 at T3 | | | | .300 | | 0.056 |
| Maternal autonomy 2 at T2 ↔ Maternal autonomy 2 at T3 | | | | .324 | | 0.116 |
| Maternal autonomy 3 at T2 ↔ Maternal autonomy 3 at T3 | | | | .266 | | 0.099 |
|  | | | |  | |  |
| Paternal autonomy 1 at T1 ↔ Paternal autonomy 1 at T2 | | | | .172 | | 0.044 |
| Paternal autonomy 1 at T1 ↔ Paternal autonomy 1 at T3 | | | | .174 | | 0.044 |
| Paternal autonomy 1 at T2 ↔ Paternal autonomy 1 at T3 | | | | .166 | | 0.043 |
| Paternal autonomy 2 at T1 ↔ Paternal autonomy 2 at T3 | | | | .241 | | 0.082 |
| Paternal autonomy 3 at T2 ↔ Paternal autonomy 3 at T3 | | | | .395 | | 0.088 |
|  | | | |  | |  |
| **Self-Reported Parental Intimacy** | | | |  | | |
|  | | | |  | | |
| ***Path Coefficients*** | | | | β | | S.E. |
| Maternal intimacy at T1 → Maternal intimacy at T2 | | | | 0.435 | | 0.097 |
| Paternal intimacy at T1 → Maternal intimacy at T2 | | | | 0.007 | | 0.057 |
| Maternal intimacy at T2 → Maternal intimacy at T3 | | | | 0.415 | | 0.084 |
| Paternal intimacy at T2 → Maternal intimacy at T3 | | | | 0.175 | | 0.119 |
|  | | | |  | |  |
| Paternal intimacy at T1 → Paternal intimacy at T2 | | | | 0.341 | | 0.095 |
| Maternal intimacy at T1 → Paternal intimacy at T2 | | | | 0.004 | | 0.060 |
| Paternal intimacy at T2 → Paternal intimacy at T3 | | | | 0.188 | | 0.089 |
| Maternal intimacy at T2 → Paternal intimacy at T3 | | | | 0.039 | | 0.061 |
|  | | | |  | |  |
| Parents’ average education → Maternal intimacy at T2 | | | | −0.086 | | 0.048 |
| Child’s sex → Maternal intimacy at T2 | | | | 0.054 | | 0.045 |
| Assisted reproductive treatment → Maternal intimacy at T2 | | | | 0.033 | | 0.045 |
| Parents’ average education → Maternal intimacy at T3 | | | | 0.012 | | 0.057 |
| Child’s sex → Maternal intimacy at T3 | | | | −0.037 | | 0.056 |
| Assisted reproductive treatment → Maternal intimacy at T3 | | | | 0.057 | | 0.056 |
|  | | | |  | |  |
| Parents’ average education → Paternal intimacy at T2 | | | | 0.007 | | 0.073 |
| Child’s sex → Paternal intimacy at T2 | | | | 0.028 | | 0.059 |
| Assisted reproductive treatment → Paternal intimacy at T2 | | | | 0.116 | | 0.055 |
| Parents’ average education → Paternal intimacy at T3 | | | | 0.038 | | 0.065 |
| Child’s sex → Paternal intimacy at T3 | | | | 0.106 | | 0.053 |
| Assisted reproductive treatment → Paternal intimacy at T3 | | | | −0.029 | | 0.055 |
|  | | | |  | |  |
| Parents’ average education → Adolescents’ reappraisal | | | | 0.089 | | 0.052 |
| Child’s sex → Adolescents’ reappraisal | | | | 0.073 | | 0.049 |
| Assisted reproductive treatment → Adolescents’ reappraisal | | | | −0.010 | | 0.049 |
| Parents’ average education → Adolescents’ suppression | | | | −0.046 | | 0.054 |
| Child’s sex → Adolescents’ suppression | | | | −0.286 | | 0.048 |
| Assisted reproductive treatment → Adolescents’ suppression | | | | 0.040 | | 0.050 |
| Parents’ average education → Adolescents’ rumination | | | | −0.048 | | 0.051 |
| Child’s sex → Adolescents’ rumination | | | | 0.133 | | 0.049 |
| Assisted reproductive treatment → Adolescents’ rumination | | | | 0.061 | | 0.048 |
|  | | | |  | |  |
| ***Correlations of Latent Variables and Covariates*** | | | | ρ | | S.E. |
| Maternal intimacy at T1 ↔ Paternal intimacy at T1 | | | | .056 | | 0.049 |
| Maternal intimacy at T2 ↔ Paternal intimacy at T2 | | | | .031 | | 0.084 |
| Maternal intimacy at T3 ↔ Paternal intimacy at T3 | | | | .095 | | 0.093 |
| Reappraisal ↔ Suppression | | | | −.025 | | 0.066 |
| Reappraisal ↔ Rumination | | | | −.167 | | 0.059 |
| Rumination ↔ Suppression | | | | .170 | | 0.061 |
|  | | | |  | |  |
| Parents’ average education ↔ Maternal intimacy at T1 | | | | −.039 | | 0.046 |
| Child’s sex ↔ Maternal intimacy at T1 | | | | −.012 | | 0.045 |
| Assisted reproductive treatment ↔ Maternal intimacy at T1 | | | | .039 | | 0.046 |
| Parents’ average education ↔ Paternal intimacy at T1 | | | | −.017 | | 0.048 |
| Child’s sex ↔ Paternal intimacy at T1 | | | | −.001 | | 0.046 |
| Assisted reproductive treatment ↔ Paternal intimacy at T1 | | | | .089 | | 0.049 |
| Parents’ average education ↔ Child’s sex | | | | .056 | | 0.036 |
| Assisted reproductive treatment ↔ Child’s sex | | | | .028 | | 0.035 |
| Parents’ average education ↔ Assisted reproductive treatment | | | | −.065 | | 0.035 |
|  | | | |  | |  |
| ***Factor Loadings*** | | | | λ | | S.E. |
| Maternal intimacy at T1 → Maternal intimacy 1 at T1 | | | | .602 | | 0.076 |
| Maternal intimacy at T1 → Maternal intimacy 2 at T1 | | | | .749 | | 0.071 |
| Maternal intimacy at T1 → Maternal intimacy 3 at T1 | | | | .569 | | 0.074 |
| Maternal intimacy at T1 → Maternal intimacy 4 at T1 | | | | .700 | | 0.090 |
| Maternal intimacy at T2 → Maternal intimacy 1 at T2 | | | | .496 | | 0.087 |
| Maternal intimacy at T2 → Maternal intimacy 2 at T2 | | | | .858 | | 0.072 |
| Maternal intimacy at T2 → Maternal intimacy 3 at T2 | | | | .395 | | 0.090 |
| Maternal intimacy at T2 → Maternal intimacy 4 at T2 | | | | .806 | | 0.035 |
| Maternal intimacy at T3 → Maternal intimacy 1 at T3 | | | | .358 | | 0.107 |
| Maternal intimacy at T3 → Maternal intimacy 2 at T3 | | | | .622 | | 0.081 |
| Maternal intimacy at T3 → Maternal intimacy 3 at T3 | | | | .446 | | 0.078 |
| Maternal intimacy at T3 → Maternal intimacy 4 at T3 | | | | .682 | | 0.073 |
|  | | | |  | |  |
| Paternal intimacy at T1 → Paternal intimacy 1 at T1 | | | | .641 | | 0.083 |
| Paternal intimacy at T1 → Paternal intimacy 2 at T1 | | | | .759 | | 0.062 |
| Paternal intimacy at T1 → Paternal intimacy 3 at T1 | | | | .662 | | 0.071 |
| Paternal intimacy at T1 → Paternal intimacy 4 at T1 | | | | .734 | | 0.083 |
| Paternal intimacy at T2 → Paternal intimacy 1 at T2 | | | | .534 | | 0.113 |
| Paternal intimacy at T2 → Paternal intimacy 2 at T2 | | | | .665 | | 0.109 |
| Paternal intimacy at T2 → Paternal intimacy 3 at T2 | | | | .512 | | 0.092 |
| Paternal intimacy at T2 → Paternal intimacy 4 at T2 | | | | .736 | | 0.115 |
| Paternal intimacy at T3 → Paternal intimacy 1 at T3 | | | | .612 | | 0.081 |
| Paternal intimacy at T3 → Paternal intimacy 2 at T3 | | | | .609 | | 0.083 |
| Paternal intimacy at T3 → Paternal intimacy 3 at T3 | | | | .603 | | 0.086 |
| Paternal intimacy at T3 → Paternal intimacy 4 at T3 | | | | .845 | | 0.070 |
|  | | | |  | |  |
| Reappraisal → Factor score of reappraisal | | | | .942 | | .004 |
| Suppression → Factor score of suppression | | | | .894 | | .006 |
| Rumination → Factor score of rumination | | | | .938 | | .005 |
|  | | | |  | |  |
| ***Error Term Correlations of Indicators*** | | | | ρ | | S.E. |
| Maternal intimacy 1 at T1 ↔ Maternal intimacy 1 at T2 | | | | .218 | | 0.057 |
| Maternal intimacy 1 at T2 ↔ Maternal intimacy 1 at T3 | | | | .084 | | 0.039 |
| Maternal intimacy 3 at T1 ↔ Maternal intimacy 3 at T2 | | | | .271 | | 0.059 |
| Paternal intimacy 1 at T1 ↔ Paternal intimacy 1 at T2 | | | | .135 | | 0.067 |
| Paternal intimacy 1 at T2 ↔ Paternal intimacy 1 at T3 | | | | .466 | | 0.122 |
| Paternal intimacy 2 at T2 ↔ Paternal intimacy 2 at T3 | | | | .283 | | 0.126 |
| Paternal intimacy 3 at T1 ↔ Paternal intimacy 3 at T2 | | | | .240 | | 0.090 |
| Paternal intimacy 3 at T2 ↔ Paternal intimacy 3 at T3 | | | | .312 | | 0.091 |
| Paternal intimacy 4 at T1 ↔ Paternal intimacy 4 at T2 | | | | .163 | | 0.091 |
| Paternal intimacy 4 at T1 ↔ Paternal intimacy 4 at T3 | | | | .257 | | 0.124 |
|  | | | |  | |  |
| **Partner-Reported Parental Intimacy** | | | | |  | |
|  | | | | |  | |
| ***Path Coefficients*** | | | | β | | S.E. |
| Maternal intimacy at T1 → Maternal intimacy at T2 | | | | 0.485 | | 0.181 |
| Paternal intimacy at T1 → Maternal intimacy at T2 | | | | −0.057 | | 0.076 |
| Maternal intimacy at T2 → Maternal intimacy at T3 | | | | 0.225 | | 0.094 |
| Paternal intimacy at T2 → Maternal intimacy at T2 | | | | −0.088 | | 0.068 |
|  | | | |  | |  |
| Paternal intimacy at T1 → Paternal intimacy at T2 | | | | 0.430 | | 0.084 |
| Maternal intimacy at T1 → Paternal intimacy at T2 | | | | 0.028 | | 0.086 |
| Paternal intimacy at T2 → Paternal intimacy at T3 | | | | 0.412 | | 0.081 |
| Maternal intimacy at T2 → Paternal intimacy at T3 | | | | −0.018 | | 0.085 |
|  | | | |  | |  |
| Parents’ average education → Maternal intimacy at T2 | | | | 0.035 | | 0.074 |
| Child’s sex → Maternal intimacy at T2 | | | | 0.015 | | 0.052 |
| Assisted reproductive treatment → Maternal intimacy at T2 | | | | 0.038 | | 0.055 |
| Parents’ average education → Maternal intimacy at T3 | | | | 0.019 | | 0.052 |
| Child’s sex → Maternal intimacy at T3 | | | | 0.010 | | 0.048 |
| Assisted reproductive treatment → Maternal intimacy at T3 | | | | 0.085 | | 0.051 |
|  | | | |  | |  |
| Parents’ average education → Paternal intimacy at T2 | | | | 0.040 | | 0.052 |
| Child’s sex → Paternal intimacy at T2 | | | | −0.057 | | 0.044 |
| Assisted reproductive treatment → Paternal intimacy at T2 | | | | 0.064 | | 0.044 |
| Parents’ average education → Paternal intimacy at T3 | | | | 0.024 | | 0.052 |
| Child’s sex → Paternal intimacy at T3 | | | | 0.090 | | 0.044 |
| Assisted reproductive treatment → Paternal intimacy at T3 | | | | −0.012 | | 0.042 |
|  | | | |  | |  |
| Parents’ average education → Adolescents’ reappraisal | | | | 0.116 | | 0.051 |
| Child’s sex → Adolescents’ reappraisal | | | | 0.089 | | 0.047 |
| Assisted reproductive treatment → Adolescents’ reappraisal | | | | −0.029 | | 0.050 |
| Parents’ average education → Adolescents’ suppression | | | | −0.034 | | 0.058 |
| Child’s sex → Adolescents’ suppression | | | | −0.297 | | 0.048 |
| Assisted reproductive treatment → Adolescents’ suppression | | | | 0.019 | | 0.050 |
| Parents’ average education → Adolescents’ rumination | | | | −0.094 | | 0.053 |
| Child’s sex → Adolescents’ rumination | | | | 0.108 | | 0.050 |
| Assisted reproductive treatment → Adolescents’ rumination | | | | 0.088 | | 0.048 |
|  | | | |  | |  |
| ***Correlations of Latent Variables and Covariates*** | | | | ρ | | S.E. |
| Maternal intimacy at T1 ↔ Paternal intimacy at T1 | | | | .126 | | 0.117 |
| Maternal intimacy at T2 ↔ Paternal intimacy at T2 | | | | .081 | | 0.104 |
| Maternal intimacy at T3 ↔ Paternal intimacy at T3 | | | | .137 | | 0.085 |
| Reappraisal ↔ Suppression | | | | −.021 | | 0.065 |
| Rumination ↔ Suppression | | | | .171 | | 0.060 |
| Reappraisal ↔ Rumination | | | | −.170 | | 0.059 |
|  | | | |  | |  |
| Parents’ average education ↔ Maternal intimacy at T1 | | | | .055 | | 0.041 |
| Child’s sex ↔ Maternal intimacy at T1 | | | | −.002 | | 0.061 |
| Assisted reproductive treatment ↔ Maternal intimacy at T1 | | | | −.004 | | 0.051 |
| Parents’ average education ↔ Paternal intimacy at T1 | | | | −.023 | | 0.044 |
| Child’s sex ↔ Paternal intimacy at T1 | | | | .028 | | 0.043 |
| Assisted reproductive treatment ↔ Paternal intimacy at T1 | | | | .146 | | 0.041 |
| Parents’ average education ↔ Child’s sex | | | | −.070 | | 0.035 |
| Assisted reproductive treatment ↔ Child’s sex | | | | .062 | | 0.037 |
| Parents’ average education ↔ Assisted reproductive treatment | | | | .029 | | 0.035 |
|  |  | | | |  | |
| ***Factor Loadings*** | | | | λ | | S.E. |
| Maternal intimacy at T1 → Maternal intimacy 1 at T1 | | | | .841 | | 0.084 |
| Maternal intimacy at T1 → Maternal intimacy 2 at T1 | | | | .386 | | 0.125 |
| Maternal intimacy at T1 → Maternal intimacy 3 at T1 | | | | .315 | | 0.120 |
| Maternal intimacy at T1 → Maternal intimacy 4 at T1 | | | | .858 | | 0.069 |
| Maternal intimacy at T2 → Maternal intimacy 1 at T2 | | | | .740 | | 0.098 |
| Maternal intimacy at T2 → Maternal intimacy 2 at T2 | | | | .841 | | 0.084 |
| Maternal intimacy at T2 → Maternal intimacy 3 at T2 | | | | .578 | | 0.076 |
| Maternal intimacy at T2 → Maternal intimacy 4 at T2 | | | | .787 | | 0.090 |
| Maternal intimacy at T3 → Maternal intimacy 1 at T3 | | | | .811 | | 0.038 |
| Maternal intimacy at T3 → Maternal intimacy 2 at T3 | | | | .758 | | 0.066 |
| Maternal intimacy at T3 → Maternal intimacy 3 at T3 | | | | .611 | | 0.067 |
| Maternal intimacy at T3 → Maternal intimacy 4 at T3 | | | | .924 | | 0.026 |
|  | | | |  | |  |
| Paternal intimacy at T1 → Paternal intimacy 1 at T1 | | | | .751 | | 0.045 |
| Paternal intimacy at T1 → Paternal intimacy 2 at T1 | | | | .827 | | 0.056 |
| Paternal intimacy at T1 → Paternal intimacy 3 at T1 | | | | .698 | | 0.049 |
| Paternal intimacy at T1 → Paternal intimacy 4 at T1 | | | | .817 | | 0.044 |
| Paternal intimacy at T2 → Paternal intimacy 1 at T2 | | | | .720 | | 0.051 |
| Paternal intimacy at T2 → Paternal intimacy 2 at T2 | | | | .838 | | 0.037 |
| Paternal intimacy at T2 → Paternal intimacy 3 at T2 | | | | .557 | | 0.057 |
| Paternal intimacy at T2 → Paternal intimacy 4 at T2 | | | | .824 | | 0.048 |
| Paternal intimacy at T3 → Paternal intimacy 1 at T3 | | | | .748 | | 0.035 |
| Paternal intimacy at T3 → Paternal intimacy 2 at T3 | | | | .910 | | 0.018 |
| Paternal intimacy at T3 → Paternal intimacy 3 at T3 | | | | .721 | | 0.033 |
| Paternal intimacy at T3 → Paternal intimacy 4 at T3 | | | | .888 | | 0.023 |
|  | | | |  | |  |
| Reappraisal → Factor score of reappraisal | | | | .942 | | 0.004 |
| Suppression → Factor score of suppression | | | | .894 | | 0.006 |
| Rumination → Factor score of rumination | | | | .938 | | 0.005 |
|  | | | |  | |  |
| ***Error Term Correlations of Indicators*** | | | | ρ | | S.E. |
| Maternal intimacy 2 at T1 ↔ Maternal intimacy 2 at T2 | | | | .462 | | 0.095 |
| Maternal intimacy 2 at T2 ↔ Maternal intimacy 2 at T3 | | | | .395 | | 0.091 |
| Maternal intimacy 3 at T1 ↔ Maternal intimacy 3 at T2 | | | | .227 | | 0.084 |
| Maternal intimacy 3 at T2 ↔ Maternal intimacy 3 at T3 | | | | .168 | | 0.078 |
| Paternal intimacy 1 at T2 ↔ Paternal intimacy 1 at T3 | | | | .241 | | 0.083 |
| Paternal intimacy 3 at T1 ↔ Paternal intimacy 3 at T2 | | | | .323 | | 0.052 |
| Paternal intimacy 3 at T1 ↔ Paternal intimacy 3 at T3 | | | | .319 | | 0.048 |
| Paternal intimacy 3 at T2 ↔ Paternal intimacy 3 at T3 | | | | .222 | | 0.044 |
| *Note.* T1 = infancy; T2 = middle childhood; T3 = late adolescence. | | | | | | |
|  | | | | | | |
