# Supplementary material for: Developmental Stage-Specific Effects of Parenting on Adolescents’ Emotion Regulation: A Longitudinal Study From Infancy to Late Adolescence
Source: Front Psychol. 2021 Jun 4;12:582770. doi: 10.3389/fpsyg.2021.582770 (PMC8211896; doi:10.3389/fpsyg.2021.582770)
Supplement: Supplementary file 7 [file Table_7.docx]

**Supplementary Material 7.** Monte Carlo Simulations: Statistical Power and Type I Error Rates for Detecting Differences Between Nested Developmental Timing Models.

|  |  | | | | | | | | | | |  |
| --- | --- | --- | --- | --- | --- | --- | --- | --- | --- | --- | --- | --- |
|  | Data-Generating Population Model | | | | | | | | | | |  |
|  | Whole  Childhood Model | | Stability  Model | | | Infancy  Model | | | Middle  Childhood Model | | |  |
| Model comparison | ∆χ^2^ | **1−β** | | ∆χ^2^ | *α/***1−β** | | ∆χ^2^ | **1−β***/α* | | ∆χ^2^ | **1−β***/α* | |
| **Self-Reported Parental Autonomy** |  |  | |  |  | |  |  | |  |  | |
| Whole Childhood Model (*df* = 340) vs. Stability Model (*df* = 352) | 54.47 | **.996**  **(.981)** | | 13.17 | *.086*  *(.025)* | | 30.71 | **.814**  **(.623)** | | 28.77 | **.769**  **(.557)** | |
| Whole Childhood Model (df = 340) vs. Infancy Model (*df* = 346) | 22.29 | **.827**  **(.661)** | | 6.71 | *.092*  *(.024)* | | 6.46 | *.084*  *(.020)* | | 22.75 | **.827**  **(.642)** | |
| Whole Childhood Model (*df* = 340) vs. Middle Childhood Model (*df* = 346) | 24.18 | **.845**  **(.699)** | | 6.88 | *.091*  *(.030)* | | 25.27 | **.870**  **(.725)** | | 6.80 | *.086*  *(.026)* | |
| Infancy Model (*df* = 346) vs.  Stability Model (*df* = 352) | 32.74 | **.968**  **(.915)** | | 6.59 | *.069*  *(.020)* | | 24.37 | **.900**  **(.754)** | | 7.58 | *.134*  *(.043)* | |
| Middle Childhood Model (*df* = 346) vs. Stability Model (*df* = 352) | 31.31 | **.949**  **(.881)** | | 6.46 | *.080*  *(.023)* | | 7.94 | *.143*  *(.041)* | | 22.18 | **.847**  **(.671)** | |
|  |  |  | |  |  | |  |  | |  |  | |
| Whole Childhood Model (*df* = 340) vs. no effects of parenting on ER patterns (*df* = 358) | 117.56 | **1.000** | | 38.88 | **.812** | | 74.25 | **1.000** | | 73.57 | **1.000** | |
| Stability Model (*df* = 352) vs.  no effects of parenting on ER patterns (*df* = 358) | 59.45 | **1.000** | | 25.19 | **.921** | | 42.11 | **.998** | | 42.28 | **.999** | |
| Infancy Model (*df* = 346) vs.  no effects of parenting on ER patterns (*df* = 358) | 93.57 | **1.000** | | 32.00 | **.854** | | 66.82 | **1.000** | | 50.36 | **.997** | |
| Middle Childhood Model (*df* = 346) vs.  no effects of parenting on ER patterns (*df* = 358) | 91.74 | **1.000** | | 31.81 | **.863** | | 49.85 | **0.995** | | 64.85 | **1.000** | |
|  |  |  | |  |  | |  |  | |  |  | |
| **Partner-Reported Parental Autonomy** |  |  | |  |  | |  |  | |  |  | |
| Whole Childhood Model (*df* = 340) vs. Stability Model (*df* = 352) | 60.88 | **.997**  **(.994)** | | 13.06 | *.074*  *(.016)* | | 31.81 | **.845**  **(.695)** | | 27.71 | **.721**  **(.526)** | |
| Whole Childhood Model (*df* = 340) vs. Infancy Model (*df* = 346) | 19.30 | **.738**  **(.547)** | | 6.81 | *.091*  *(.021)* | | 6.87 | *.098*  *(.033)* | | 18.26 | **.712**  **(.517)** | |
| Whole Childhood Model (*df* = 340) vs. Middle Childhood Model (*df* = 346) | 21.01 | **.780**  **(.608)** | | 6.72 | *.094*  *(.024)* | | 22.21 | **.829**  **(.639)** | | 6.65 | *.088*  *(.030)* | |
| Infancy Model (*df* = 346) vs.  Stability Model (*df* = 352) | 41.60 | **.997**  **(.986)** | | 6.36 | *.069*  *(.009)* | | 24.93 | **.916**  **(.793)** | | 9.95 | *.277*  *(.124)* | |
| Middle Childhood Model (*df* = 346) vs. Stability Model (*df* = 352) | 39.89 | **.994**  **(.963)** | | 6.48 | *.071*  *(.014)* | | 10.87 | *.319*  *(.157)* | | 21.11 | **.826**  **(.638)** | |
|  |  |  | |  |  | |  |  | |  |  | |
| Whole Childhood Model (*df* = 340) vs. no effects of parenting on ER patterns (*df* = 358) | 123.07 | **1.000** | | 39.95 | **.845** | | 71.64 | **1.000** | | 73.33 | **1.000** | |
| Stability Model (*df* = 352) vs.  no effects of parenting on ER patterns (*df* = 358) | 60.25 | **1.000** | | 26.24 | **.946** | | 38.92 | **.999** | | 44.33 | **.999** | |
| Infancy Model (df = 346) vs.  no effects of parenting on ER patterns (*df* = 358) | 102.27 | **1.000** | | 32.99 | **.878** | | 64.02 | **1.000** | | 54.95 | **.999** | |
| Middle Childhood Model (*df* = 346) vs.  no effects of parenting on ER patterns (*df* = 358) | 100.25 | **1.000** | | 32.97 | **.888** | | 49.77 | **.997** | | 65.87 | **1.000** | |
|  |  |  | |  |  | |  |  | |  |  | |
|  |  |  | |  |  | |  |  | |  |  | |
| **Self-Reported Parental Intimacy** |  |  | |  |  | |  |  | |  |  | |
| Whole Childhood Model (*df* = 339) vs. Stability Model (*df* = 351) | 74.26 | **.998**  **(.998)** | | 13.26 | *.099*  *(.026)* | | 38.01 | **.936**  **(.812)** | | 27.26 | **.678**  **(.475)** | |
| Whole Childhood Model (*df* = 339) vs. Infancy Model (*df* = 345) | 19.38 | **.702**  **(.510)** | | 6.86 | *.091*  *(.026)* | | 7.08 | *.120*  *(.036)* | | 18.41 | **.678**  **(.487)** | |
| Whole Childhood Model (*df* = 339) vs. Middle Childhood Model (*df* = 345) | 28.02 | **.874**  **(.745)** | | 6.93 | *.100*  *(.036)* | | 28.93 | **.906**  **(.794)** | | 6.88 | *.105*  *(.036)* | |
| Infancy Model (*df* = 345) vs.  Stability Model (*df* = 351) | 54.42 | **.994**  **(.989)** | | 6.52 | *.088*  *(.020)* | | 31.35 | **.966**  **(.896)** | | 9.61 | *.215*  *(.109)* | |
| Middle Childhood Model (*df* = 345) vs. Stability Model (*df* = 351) | 51.25 | **.985**  **(.958)** | | 6.58 | *.092*  *(.025)* | | 12.34 | *.381*  *(.212)* | | 21.14 | **.757**  **(.597)** | |
|  |  |  | |  |  | |  |  | |  |  | |
| Whole Childhood Model (*df* = 339) vs. no effects of parenting on ER patterns (*df* = 357) | 145.96 | **1.000** | | 43.56 | **.892** | | 79.45 | **.999** | | 87.62 | **1.000** | |
| Stability Model (*df* = 351) vs.  no effects of parenting on ER patterns (*df* = 357) | 71.20 | **1.000** | | 29.61 | **.956** | | 40.99 | **.994** | | 59.10 | **.999** | |
| Infancy Model (*df* = 345) vs.  no effects of parenting on ER patterns (*df* = 357) | 125.99 | **1.000** | | 36.62 | **.924** | | 71.61 | **.999** | | 69.42 | **1.000** | |
| Middle Childhood Model (*df* = 345) vs.  no effects of parenting on ER patterns (*df* = 357) | 117.79 | **1.000** | | 36.33 | **.927** | | 52.79 | **.995** | | 79.96 | **1.000** | |
|  |  |  | |  |  | |  |  | |  |  | |
| **Partner-Reported Parental Intimacy** |  |  | |  |  | |  |  | |  |  | |
| Whole Childhood Model (*df* = 344) vs. Stability Model (*df* = 356) | 79.08 | **1.000**  **(1.000)** | | 13.65 | *.102*  *(.036)* | | 37.55 | **.933**  **(.812)** | | 32.42 | **.853**  **(.683)** | |
| Whole Childhood Model (*df* = 344) vs. Infancy Model (*df* = 350) | 20.32 | **.771**  **(.578)** | | 7.20 | *.110*  *(.032)* | | 7.02 | *.103*  *(.027)* | | 19.85 | **.767**  **(.561)** | |
| Whole Childhood Model (*df* = 344) vs. Middle Childhood Model (*df* = 350) | 28.00 | **.864**  **(.732)** | | 7.31 | *.118*  *(.041)* | | 27.06 | **.864**  **(.733)** | | 7.55 | *.131*  *(.058)* | |
| Infancy Model (*df* = 350) vs.  Stability Model (*df* = 356) | 54.04 | **.999**  **(.999)** | | 6.69 | *.080*  *(.024)* | | 31.20 | **.966**  **(.900)** | | 13.20 | *.458*  *(.262)* | |
| Middle Childhood Model (*df* = 350) vs. Stability Model (*df* = 356) | 52.99 | **.998**  **(.996)** | | 6.57 | *.072*  *(.016)* | | 13.27 | *.470*  *(.277)* | | 24.73 | **.900**  **(.744)** | |
|  |  |  | |  |  | |  |  | |  |  | |
| Whole Childhood Model (*df* = 344) vs. no effects of parenting on ER patterns (*df* = 362) | 128.18 | **1.000** | | 44.46 | **.904** | | 73.94 | **1.000** | | 79.10 | **1.000** | |
| Stability Model (*df* = 356) vs.  no effects of parenting on ER patterns (*df* = 362) | 49.14 | **1.000** | | 29.52 | **.969** | | 35.58 | **.992** | | 45.08 | **.999** | |
| Infancy Model (df = 350) vs.  no effects of parenting on ER patterns (*df* = 362) | 108.77 | **1.000** | | 37.22 | **.927** | | 66.36 | **1.000** | | 59.78 | **1.000** | |
| Middle Childhood Model (*df* = 350) vs.  no effects of parenting on ER patterns (*df* = 362) | 100.45 | **1.000** | | 36.65 | **.931** | | 48.83 | **.985** | | 70.09 | **1.000** | |
| *Note. N* = 885. The statistical power and Type I error rate using the nominal alpha level have been reported without parentheses; the statistical power and Type I error rate reported in parentheses have been adjusted using the Bonferroni-corrected alpha level (= .05/5). ∆χ^2^ = the average scaled chi-square difference test value of 1000 replicated simulations; **1−β** = statistical power for detecting the difference in the scaled chi-square difference test with the alpha level of .05 (bolded values); *α =* Type I error rate for falsely detecting a difference in the scaled chi-square test with the alpha level of .050 (italicized values); ER = emotion regulation. | | | | | | | | | | | | |
|  | | | | | | | | | | | | |
