# Supplementary material for: Developmental Stage-Specific Effects of Parenting on Adolescents’ Emotion Regulation: A Longitudinal Study From Infancy to Late Adolescence
Source: Front Psychol. 2021 Jun 4;12:582770. doi: 10.3389/fpsyg.2021.582770 (PMC8211896; doi:10.3389/fpsyg.2021.582770)
Supplement: Supplementary file 8 [file Table_8.docx]

Supplementary Material 8: Self-Reported and Partner-Reported Developmental Timing Models of Combined Autonomy and Intimacy

According to the development-cohesion model of family relationships, both high parental autonomy and high parental intimacy are necessary for optimal child development, whereas deficits in one or both indicate a more problematic developmental environment (Mattejat and Scholz, 1994). In our main analyses, we only modeled autonomy and intimacy separately, which may have explained our null results. To take this possibility into account, we inspected the robustness of our results with another modeling strategy for (a) self-reported and (b) partner-reported parenting data. This modeling strategy enabled us to explore the effects of the combined high autonomy and high intimacy (vs. low autonomy and low intimacy) on adolescents’ emotion regulation (ER) patterns.

# MODELING STRATEGY

In building the alternative models, we first parceled the indicators of autonomy (four items of each measurement point) into the average variables at infancy (T1), middle childhood (T2), and late adolescence (T3) separately for mothers and fathers. The same procedure was also executed for the intimacy indicators at T1, T2, and T3 separately for mothers and fathers. These pairs of autonomy and intimacy variables were used as the two reflective indicators of each latent parenting variable at each measurement point. The correlations between the maternal autonomy and intimacy indicator variables were .42 at T1, .39 at T2, and .49 at T3 for self-reports, and .39 at T1, .39 at T2, and .51 at T3 for partner-reports. The correlations between the paternal autonomy and intimacy indicator variables were .47 at T1, .59 at T2, and .53 at T3 for self-reports, and .51 at T1, .49 at T2, and .51 at T3 for partner-reports. All zero-order correlations between the variables used in the self- and partner-reported models of combined autonomy and intimacy are presented in Supplementary Table 8A. The descriptive statistics for the variables are presented in Supplementary Table 8B.

The created measurement models of the self- and partner-reported combined autonomy and intimacy included six latent variables (mothering and fathering at T1, T2, and T3). The loadings of both autonomy and intimacy indicators of each latent variable were fixed to the same value. Thus, each latent variable of mothering and fathering at T1, T2, and T3, reflected the shared variance of autonomy and intimacy. These latent variables captured the continuum of optimal parenting in terms of *high autonomy and high intimacy* versus *low autonomy and low intimacy*. This modeling strategy allowed us to investigate the combined effects of the autonomy and intimacy at T1, T2, and T3 on adolescents’ ER patterns, while also controlling the stability of parenting and the parenting effects in other developmental stages, a prerequisite for testing our sensitive periods hypotheses. It also allowed us to model the unique impact of mothers and fathers, one of our main research questions.

We followed the same modeling strategy as in our main models reported in the manuscript. The correlations between the latent mothering and fathering variables were estimated. The error term correlations of the corresponding autonomy and intimacy indicators were also estimated across time as we assumed these indicators to share some unique method variance. The time invariance of each maternal and paternal dimension and the invariance between mothers and fathers were assessed. After testing the invariance structure, we fixed the error term correlations of corresponding indicators, which did not weaken the model fit, to the same value or zero in order to minimize the parameter-to-*N* ratio. Then, we compared two models: (a) the basic first-order autoregressive cross-lagged model and (b) the second-order autoregressive cross-lagged model in which the second-order autoregressive paths (from T1 to T3) of both mothering and fathering were estimated. Of these models, we included the model with a better fit as such to the developmental timing models.

The four developmental timing models and their submodels were tested and compared using the same modeling strategy described in the manuscript (see Developmental Timing Models section in Methods). Thus, the models only differed in their effects of parenting on adolescents’ ER patterns.

Missing data were handled with the same procedure as in the main models (Howard et al., 2015; see Missing data section in Methods). In sum, first, we formed principal component auxiliary variables for self-reported (20 principal components explaining 50 % of the overall variance) and partner-reported models (19 principal component variables explaining 50% of the overall variance) separately. Then, we used the full information maximum likelihood estimation that utilized the created auxiliary variables to improve model estimation in both self- and partner-reported models (Howard et al., 2015). When modeling the partner-reported developmental timing models, we had to exclude four auxiliary variables due to the memory limitations in the estimation procedure. Thus, we used 15 principal component auxiliary variables, explaining 44% of the variance in the original variables that were used to obtain the principal components.

# RESULTS

## **Parental Measurement Models**

The results regarding the measurement models of the self- and partner-reported parenting are shown in Supplementary Table 8C. Both models showed adequate model fit when the configural time invariance structure was tested (self-reported: scaled χ^2^ (33, *N* = 885) = 39.65, *p* = .198, CFI = .993, RMSEA = .014, SRMR = .035; partner-reported: scaled χ^2^ (33, *N* = 885) = 34.69, *p* = .387, CFI = .998, RMSEA = .009, SRMR = .043). However, neither of the models met any stricter assumptions of the time invariance (Supplementary Table 8C). Similarly, only the configural factorial invariance between the mothers and fathers was held in both models (Supplementary Table 8C). In the self-reported model, the standardized factor loadings were .46–.69 for parental autonomy indicators and .69–.85 for parental intimacy indicators. In the partner-reported model, the standardized factor loadings were .44–.68 for parental autonomy indicators and .66–.85 for parental intimacy indicators. In the comparisons of the first- and second-order autoregressive cross-lagged models, no difference existed between the models (Supplementary Table 8C). Thus, the first-order autoregressive structure was included in both self-reported and partner-reported models.

## **Developmental Timing Models**

The fit indices of all four developmental timing models were adequate in both modeling situations (self-reported parenting models: CFI = .995–1.000, RMSEA = .001–.009, SRMR = .036–.038; partner-reported parenting models: CFI = .979–.983, RMSEA = .015–.018, SRMR = .045–.047). The comparisons of developmental timing models showed consistent results across both modeling situations: Contrary to our first hypothesis, no differences in the model fit were found between the developmental timing models (Supplementary Table 8D). As a result, the most parsimonious Stability Model was considered as the most suitable model for both self- and partner-reported models. In other words, mothering and fathering in infancy and middle childhood did not show any predictive power on adolescents’ ER patterns over and above its temporal stability. Thus, the interpretation of these results was the same as the results regarding the main models reported in the manuscript.

Next, in both modeling situations, we compared the selected Stability Model and its submodel in which we also fixed the effects of parenting at T3 on ER patterns to zero to test whether parenting had any effects on adolescents’ ER patterns. Contrary to our second hypothesis, in the self-reported modeling situation, there was no difference in the model fit between the Stability Model and its submodel with no effects of parenting on adolescents’ ER patterns (Supplementary Table 8D). As a result, no other comparisons were needed to test the role of parents’ gender (third hypothesis) and the effects of parenting on each specific ER pattern (fourth hypothesis). Thus, in the self-reported modeling situation, we found no effects of mothering or fathering in infancy, middle childhood, and late adolescence on adolescents’ ER patterns.

In the partner-reported modeling situation, the Stability Model instead showed better fit than its submodel with no effects of parenting on adolescents’ ER patterns (Supplementary Table 8D). Thus, in this modeling situation, combined autonomy and intimacy of parenting in late adolescence had effects on adolescents’ ER patterns. Further model comparisons regarding parents’ gender indicated that only fathering but not mothering in late adolescence had effects on adolescent’s ER patterns (Supplementary Table 8D). Finally, model comparisons showed that it was specifically rumination on which fathering had an effect (Supplementary Table 8D). In other words, high autonomous and intimate fathering (i.e., optimal fathering) in late adolescence was linked to adolescents’ less rumination, β = −0.182 *SE* = 0.067, *p* = .007.

The final selected models for self- and partner-reported combined autonomy and intimacy of parenting are shown in Supplementary Figure 8. Like the main models reported in the manuscript, the self-reported model completely contradicted our hypotheses, suggesting that neither mothering nor fathering in any developmental stage had predictive power on adolescents’ ER patterns. In turn, the partner-reported model showed one effect of high autonomous and intimate fathering in late adolescence on adolescents’ lower rumination. However, it should be noted that five out of six of our modeling situations did not find this effect in late adolescence, and, in total, our models considered 36 effects of mothering and fathering in late adolescence on adolescents’ ER patterns. This multiple testing increases the risk that the single detected effect can be false positive. Therefore, it seemed safest to weigh other results in our interpretation. Regarding other model parts, the autoregressive paths of mothering and fathering showed small to moderate temporal stability, whereas the cross-lagged paths between mothering and fathering were small at best (Supplementary Figure 8).

In sum, the interpretation of our findings reported in this Supplementary Material largely corresponded with the main models reported in the manuscript. Thus, the overall picture of the results was not substantially dependent on whether parental autonomy and intimacy were considered together or separately. It should still be noted that due to statistical impracticality (e.g., model complexity, high parameter-to-*N* ratio), we did not directly test the interaction effects of parental autonomy and intimacy. In other words, our analyses did not consider the combinations of *low autonomy and high intimacy* or *high autonomy and low intimacy*. Nevertheless, in our alternative modeling strategy, the latent variables of mothering and fathering still reflected the continuum between *high autonomy and high intimacy* and *low autonomy and low intimacy*. Therefore, it seems highly unlikely that our null results on developmental timing effects could be explained by our focus on the separate main effects of autonomy and intimacy.

# REFERENCES

Mattejat, F., and Scholz, M. (1994). Das subjektive familienbild (SFB) [Subjective Family Picture]. Göttingen: Hogrefe.

Howard, W. J., Rhemtulla, M., and Little, T. D. (2015). Using principal components as auxiliary variables in missing data estimation. *Multivariate Behavioral Research* 50, 285–299. [doi: 10.1080/00273171.2014.999267](https://doi.org/10.1080/00273171.2014.999267)

Supplementary **Table 8A.** Correlations Between Variables in the Self-Reported and Partner-Reported Combined Autonomy and Intimacy Models.

|  |  |  |  |  |  |  |  |  |  |  |  |  |  |  |  |  |  |  |  |  |  |  |  |  |  |  |  |  |  |  |
| --- | --- | --- | --- | --- | --- | --- | --- | --- | --- | --- | --- | --- | --- | --- | --- | --- | --- | --- | --- | --- | --- | --- | --- | --- | --- | --- | --- | --- | --- | --- |
|  | 1 | 2 | 3 | 4 | 5 | 6 | 7 | 8 | 9 | 10 | 11 | 12 | 13 | 14 | 15 | 16 | 17 | 18 | 19 | 20 | 21 | 22 | 23 | 24 | 25 | 26 | 27 | 28 | 29 | 30 |
| 1. Maternal autonomy T1 (S) | – |  |  |  |  |  |  |  |  |  |  |  |  |  |  |  |  |  |  |  |  |  |  |  |  |  |  |  |  |  |
| 2. Maternal autonomy T2 (S) | **.33** | – |  |  |  |  |  |  |  |  |  |  |  |  |  |  |  |  |  |  |  |  |  |  |  |  |  |  |  |  |
| 3. Maternal autonomy T3 (S) | **.38** | **.42** | – |  |  |  |  |  |  |  |  |  |  |  |  |  |  |  |  |  |  |  |  |  |  |  |  |  |  |  |
| 4. Paternal autonomy T1 (S) | **.16** | .08 | .08 | – |  |  |  |  |  |  |  |  |  |  |  |  |  |  |  |  |  |  |  |  |  |  |  |  |  |  |
| 5. Paternal autonomy T2 (S) | **.17** | **.14** | **.15** | **.40** | – |  |  |  |  |  |  |  |  |  |  |  |  |  |  |  |  |  |  |  |  |  |  |  |  |  |
| 6. Paternal autonomy T3 (S) | −.07 | −.05 | .10 | **.28** | **.39** | – |  |  |  |  |  |  |  |  |  |  |  |  |  |  |  |  |  |  |  |  |  |  |  |  |
| 7. Maternal intimacy T1 (S) | **.42** | **.15** | **.12** | **.10** | −.02 | −.07 | – |  |  |  |  |  |  |  |  |  |  |  |  |  |  |  |  |  |  |  |  |  |  |  |
| 8. Maternal intimacy T2 (S) | **.22** | **.39** | **.21** | .05 | .04 | −.05 | **.43** | – |  |  |  |  |  |  |  |  |  |  |  |  |  |  |  |  |  |  |  |  |  |  |
| 9. Maternal intimacy T3 (S) | **.17** | **.23** | **.49** | .06 | .11 | **.12** | **.26** | **.35** | – |  |  |  |  |  |  |  |  |  |  |  |  |  |  |  |  |  |  |  |  |  |
| 10. Paternal intimacy T1 (S) | .05 | −.03 | .03 | **.47** | **.26** | **.15** | .05 | .04 | .03 | – |  |  |  |  |  |  |  |  |  |  |  |  |  |  |  |  |  |  |  |  |
| 11. Paternal intimacy T2 (S) | **.19** | **.15** | **.26** | **.28** | **.59** | **.25** | .09 | .11 | **.21** | **.44** | – |  |  |  |  |  |  |  |  |  |  |  |  |  |  |  |  |  |  |  |
| 12. Paternal intimacy T3 (S) | −.06 | −.01 | .11 | **.15** | **.25** | **.53** | −.06 | .04 | **.16** | **.20** | **.39** | – |  |  |  |  |  |  |  |  |  |  |  |  |  |  |  |  |  |  |
| 13. Maternal autonomy T1 (P) | **.26** | .09 | **.12** | **.62** | **.27** | **.14** | .07 | −.03 | −.01 | **.38** | **.20** | .05 | – |  |  |  |  |  |  |  |  |  |  |  |  |  |  |  |  |  |
| 14. Maternal autonomy T2 (P) | **.25** | **.17** | **.20** | **.29** | **.62** | **.24** | .04 | .00 | .06 | **.19** | **.46** | **.18** | **.47** | – |  |  |  |  |  |  |  |  |  |  |  |  |  |  |  |  |
| 15. Maternal autonomy T3 (P) | .09 | .11 | **.21** | **.25** | **.24** | **.36** | .00 | −.05 | **.16** | **.16** | **.24** | **.40** | **.38** | **.43** | – |  |  |  |  |  |  |  |  |  |  |  |  |  |  |  |
| 16. Paternal autonomy T1 (P) | **.44** | **.18** | **.19** | **.25** | **.16** | .06 | **.32** | **.17** | **.19** | **.11** | **.21** | .02 | **.15** | **.20** | **.14** | – |  |  |  |  |  |  |  |  |  |  |  |  |  |  |
| 17. Paternal autonomy T2 (P) | **.23** | **.30** | **.29** | **.14** | **.25** | −.03 | **.14** | **.21** | **.19** | .10 | **.24** | −.02 | .10 | **.12** | .03 | **.30** | – |  |  |  |  |  |  |  |  |  |  |  |  |  |
| 18. Paternal autonomy T3 (P) | **.15** | **.19** | **.27** | **.16** | **.18** | .08 | **.19** | **.20** | **.22** | .04 | **.22** | .00 | .06 | .12 | .06 | **.27** | **.39** | – |  |  |  |  |  |  |  |  |  |  |  |  |
| 19. Maternal intimacy T1 (P) | **.10** | .01 | −.04 | **.38** | **.18** | .04 | **.14** | .09 | .00 | **.59** | **.25** | .07 | **.39** | **.16** | **.16** | **.12** | **.12** | .06 | – |  |  |  |  |  |  |  |  |  |  |  |
| 20. Maternal intimacy T2 (P) | **.19** | .12 | **.17** | **.27** | **.43** | **.19** | **.20** | **.19** | **.20** | **.38** | **.58** | **.27** | **.17** | **.39** | **.17** | **.18** | **.17** | **.21** | **.37** | – |  |  |  |  |  |  |  |  |  |  |
| 21. Maternal intimacy T3 (P) | .06 | .07 | **.14** | **.16** | .10 | **.33** | .10 | .10 | **.26** | **.21** | **.17** | **.33** | **.14** | **.15** | **.51** | **.16** | **.14** | **.17** | **.17** | **.29** | – |  |  |  |  |  |  |  |  |  |
| 22. Paternal intimacy T1 (P) | **.22** | **.14** | .09 | .06 | .05 | .01 | **.34** | **.23** | **.22** | **.21** | **.15** | .11 | **.15** | **.20** | .10 | **.51** | **.13** | **.16** | **.13** | .07 | .09 | – |  |  |  |  |  |  |  |  |
| 23. Paternal intimacy T2 (P) | **.15** | **.21** | **.19** | .05 | **.16** | −.01 | **.18** | **.31** | **.17** | .07 | **.30** | .09 | **.11** | **.15** | **.14** | **.24** | **.49** | **.21** | .10 | .11 | .01 | **.42** | – |  |  |  |  |  |  |  |
| 24. Paternal intimacy T3 (P) | **.12** | **.15** | **.21** | .04 | .11 | .07 | **.16** | **.25** | **.24** | −.03 | **.17** | **.21** | .01 | .11 | **.19** | **.24** | **.27** | **.51** | −.03 | .14 | **.22** | **.30** | **.50** | – |  |  |  |  |  |  |
| 25. Reappraisal | .08 | .05 | .07 | .01 | .02 | .09 | .02 | .06 | .04 | .01 | .02 | .09 | .01 | .07 | .11 | .08 | −.03 | .03 | .07 | .11 | **.13** | .05 | .04 | **.13** | – |  |  |  |  |  |
| 26. Suppression | .06 | .00 | −.06 | −.04 | −.01 | −.08 | .01 | −.02 | −.09 | −.06 | −.01 | **−.13** | −.04 | .00 | −.03 | .01 | .01 | −.01 | .00 | −.03 | −.02 | .00 | −.03 | −.01 | −.06 | – |  |  |  |  |
| 27. Rumination | .00 | −.09 | −.05 | −.09 | −.13 | **−.17** | −.07 | −.01 | −.03 | −.04 | −.03 | −.08 | .06 | −.03 | −.04 | −.07 | −.07 | **−.14** | −.08 | −.03 | −.02 | −.01 | −.06 | **−.15** | **−.17** | **.13** | – |  |  |  |
| 28. Parents’ education level T1 | −.06 | −.06 | −.06 | .04 | .02 | −.06 | −.05 | −.06 | −.04 | −.03 | .01 | −.03 | −.04 | .08 | −.10 | .05 | −.01 | .05 | .02 | .01 | .03 | −.01 | .05 | .02 | **.10** | −.05 | −.08 | – |  |  |
| 29. ART | −.06 | .02 | .00 | −.05 | .03 | .03 | .05 | .03 | .07 | −.02 | .05 | .01 | −.01 | .02 | .02 | −.01 | .01 | −.03 | .04 | .02 | .06 | **.13** | **.10** | .06 | −.03 | .03 | **.09** | −.07 | – |  |
| 30. Child's sex | −.01 | .08 | **.10** | .03 | −.01 | .09 | .01 | .05 | .05 | .09 | .05 | **.16** | .05 | .02 | .07 | −.01 | .00 | .03 | .01 | −.03 | .02 | .06 | .00 | .07 | .08 | **−.28** | **.12** | .07 | .03 | – |
| *Note.* In bolded values p < .050. T1 = Infancy; T2 = Middle childhood; T3 = Late adolescence; (S) = Self-reported; (P) = Partner-reported; ART = Assisted reproduction treatment. | | | | | | | | | | | | | | | | | | | | | | | | | | | | | | |

Supplementary **Table 8B.** Descriptive Statistics of Variables in the Self-Reported and Partner-Reported Combined Autonomy and Intimacy Models.

|  |  |  |  |  |  |  |  |  |
| --- | --- | --- | --- | --- | --- | --- | --- | --- |
| Variable | *n* | *M* | *SD* | *Mdn* | min | max | skewness | kurtosis |
| 1. Maternal autonomy T1 (S) | 544 | 6.30 | 0.65 | 6.50 | 3.00 | 7.00 | −1.22 | 2.13 |
| 2. Maternal autonomy T2 (S) | 519 | 6.19 | 0.70 | 6.25 | 2.50 | 7.00 | −1.26 | 2.98 |
| 3. Maternal autonomy T3 (S) | 449 | 5.95 | 0.80 | 6.00 | 3.00 | 7.00 | −0.85 | 0.51 |
| 4. Paternal autonomy T1 (S) | 502 | 6.11 | 0.75 | 6.25 | 3.75 | 7.00 | −0.85 | 0.35 |
| 5. Paternal autonomy T2 (S) | 295 | 6.04 | 0.72 | 6.00 | 2.75 | 7.00 | −0.84 | 1.24 |
| 6. Paternal autonomy T3 (S) | 357 | 5.64 | 0.83 | 5.75 | 3.25 | 7.00 | −0.52 | −0.19 |
| 7. Maternal intimacy T1 (S) | 543 | 6.80 | 0.36 | 7.00 | 4.00 | 7.00 | −3.50 | 17.94 |
| 8. Maternal intimacy T2 (S) | 519 | 6.65 | 0.46 | 6.75 | 4.00 | 7.00 | −2.12 | 6.43 |
| 9. Maternal intimacy T3 (S) | 449 | 6.49 | 0.61 | 6.75 | 3.75 | 7.00 | −1.66 | 3.03 |
| 10. Paternal intimacy T1 (S) | 502 | 6.66 | 0.50 | 6.75 | 4.00 | 7.00 | −2.33 | 7.40 |
| 11. Paternal intimacy T2 (S) | 295 | 6.41 | 0.61 | 6.50 | 3.50 | 7.00 | −1.50 | 3.18 |
| 12. Paternal intimacy T3 (S) | 357 | 6.03 | 0.85 | 6.25 | 3.25 | 7.00 | −0.89 | 0.14 |
| 13. Maternal autonomy T1 (P) | 500 | 6.23 | 0.76 | 6.25 | 2.50 | 7.00 | −1.15 | 1.61 |
| 14. Maternal autonomy T2 (P) | 293 | 6.05 | 0.80 | 6.25 | 2.50 | 7.00 | −1.06 | 1.29 |
| 15. Maternal autonomy T3 (P) | 353 | 5.56 | 1.08 | 5.75 | 1.75 | 7.00 | −0.76 | 0.18 |
| 16. Paternal autonomy T1 (P) | 541 | 5.94 | 0.94 | 6.00 | 2.00 | 7.00 | −1.38 | 2.49 |
| 17. Paternal autonomy T2 (P) | 501 | 6.06 | 0.93 | 6.25 | 1.75 | 7.00 | −1.46 | 2.87 |
| 18. Paternal autonomy T3 (P) | 438 | 5.74 | 1.19 | 6.00 | 1.00 | 7.00 | −1.07 | 0.66 |
| 19. Maternal intimacy T1 (P) | 500 | 6.76 | 0.45 | 7.00 | 4.00 | 7.00 | −3.06 | 11.83 |
| 20. Maternal intimacy T2 (P) | 292 | 6.52 | 0.68 | 6.75 | 2.75 | 7.00 | −2.20 | 6.11 |
| 21. Maternal intimacy T3 (P) | 353 | 6.05 | 1.04 | 6.25 | 1.00 | 7.00 | −1.52 | 2.62 |
| 22. Paternal intimacy T1 (P) | 541 | 6.64 | 0.62 | 6.75 | 3.00 | 7.00 | −2.88 | 10.13 |
| 23. Paternal intimacy T2 (P) | 501 | 6.26 | 0.87 | 6.50 | 1.25 | 7.00 | −1.88 | 4.40 |
| 24. Paternal intimacy T3 (P) | 439 | 5.80 | 1.22 | 6.00 | 1.00 | 7.00 | −1.39 | 1.89 |
| 25. Reappraisal | 437 | 0.00 | 1.06 | 0.06 | −2.92 | 2.30 | −0.31 | −0.03 |
| 26. Suppression | 437 | 0.00 | 1.12 | 0.00 | −2.10 | 2.78 | 0.19 | −0.62 |
| 27. Rumination | 437 | 0.00 | 1.06 | −0.17 | −1.51 | 3.84 | 0.87 | 0.60 |
| 28. Parents’ education level T1 | 806 | 2.87 | 0.86 | 3.00 | 1.00 | 4.00 | −0.36 | −0.78 |
| 29. ART | 885 | 0.50 | 0.50 | 1.00 | 0.00 | 1.00 | 0.00 | −2.00 |
| 30. Child's sex | 806 | 0.50 | 0.50 | 1.00 | 0.00 | 1.00 | −0.02 | −2.00 |
| *Note.* T1 = Infancy; T2 = Middle childhood; T3 = Late adolescence; (S) = Self−reported; (P) = Partner−reported; ART = Assisted reproduction treatment. | | | | | | | | |
|  | | | | | | | | |

Supplementary **Table 8C.** Testing Measurement Models of Self-Reported and Partner-Reported Combined Autonomy and Intimacy.

|  | | | | | | | |
| --- | --- | --- | --- | --- | --- | --- | --- |
| Model | *df* | Scaled  χ^2^ | CFI | RMSEA | SRMR | Scaled  ∆χ^2^ test | *p* |
| **Self-Reported Combined Autonomy and Intimacy** |  |  |  |  |  |  |  |
| ***Time Invariance*** |  |  |  |  |  |  |  |
| Configural time invariance | 33 | 39.65 | .993 | .014 | .035 |  |  |
| Weak time invariance | 37 | 85.73 | .946 | .040 | .182 | 25.11 | < .001 |
| ***Factorial Invariance Between Mothering and Fathering*** |  |  |  |  |  |  |  |
| Configural time invariance + | 33 | 39.65 | .993 | .014 | .035 |  |  |
| Configural factorial invariance |  |  |  |  |  |  |  |
| Weak factorial invariance | 36 | 64.02 | .970 | .030 | .162 | 13.79 | .003 |
| ***Fixed Error Term Correlations of Corresponding Indicators*** |  |  |  |  |  |  |  |
| Configural time invariance + configural factorial invariance | 33 | 39.65 | .993 | .014 | .035 |  |  |
| Fixing the correlations of indicator error terms that do not weaken model fit to the same value or zero | 41 | 44.40 | .997 | .010 | .041 | 5.39 | .715 |
| ***Autoregressive Cross-Lagged Models*** |  |  |  |  |  |  |  |
| Second-order autoregressive cross-lagged model | 43 | 48.01 | .995 | .011 | .043 |  |  |
| First-order autoregressive cross-lagged model | 45 | 46.80 | .998 | .007 | .044 | 0.23 | .894 |
| **Partner-Reported Combined Autonomy and Intimacy** |  |  |  |  |  |  |  |
| ***Time Invariance*** |  |  |  |  |  |  |  |
| Configural time invariance | 33 | 34.69 | .998 | .009 | .043 |  |  |
| Weak time invariance | 37 | 94.60 | .918 | .048 | .136 | 35.52 | < .001 |
| ***Factorial Invariance Between Mothering and Fathering*** |  |  |  |  |  |  |  |
| Configural time invariance + | 33 | 34.69 | .998 | .009 | .043 |  |  |
| Configural factorial invariance |  |  |  |  |  |  |  |
| Weak factorial invariance | 36 | 70.29 | .953 | .037 | .183 | 23.10 | < .001 |
| ***Fixed Error Term Correlations of Corresponding Indicators*** |  |  |  |  |  |  |  |
| Configural time invariance + configural factorial invariance | 33 | 34.69 | .998 | .009 | .043 |  |  |
| Fixing the correlations of indicator error terms that do not weaken model fit to the same value or zero | 41 | 48.29 | .990 | .015 | .052 | 13.31 | .102 |
| ***Autoregressive Cross-Lagged Models*** |  | 56.54 | .986 | .017 |  |  |  |
| Second-order autoregressive cross-lagged model | 43 | 52.13 | .989 | .016 | .054 |  |  |
| First-order autoregressive cross-lagged model | 45 | 56.54 | .985 | .018 | .058 | 4.69 | .096 |
| *Note. N* = 885. In the ∆χ^2^ tests, a model is compared to a more complex model above (e.g., a weak time invariance model is compared to a configural time invariance model). CFI = robust comparative fit index; RMSEA = robust root-mean-square error of approximation; SRMR = standardized root mean square residual. | | | | | | | |

Supplementary **Table 8D.** Comparisons of Developmental Timing Models for Self- and Partner-Reported Combined Autonomy and Intimacy.

|  | | | | | | | |
| --- | --- | --- | --- | --- | --- | --- | --- |
| Model Comparison | ∆*df* | Scaled  ∆χ^2^ test | *p* | ∆AIC | ∆*R*^2^  Reappraisal | ∆*R*^2^  Suppression | ∆*R*^2^  Rumination |
| **Self-reported Combined Autonomy and Intimacy** |  |  |  |  |  |  |  |
| Stability Model (*df* = 93) vs. |  |  |  |  |  |  |  |
| Infancy Model | 6 | 4.13 | .658 | 8.30 | .001 | .004 | .013 |
| Middle Childhood Model | 6 | 3.22 | .781 | 8.78 | .007 | .010 | .004 |
| Whole Childhood Model | 12 | 6.62 | .882 | 17.91 | .008 | .013 | .014 |
| Infancy Model (*df* = 87) vs. |  |  |  |  |  |  |  |
| Middle Childhood Model | 0 |  |  | 0.49 | .006 | .006 | −.009 |
| Whole Childhood Model | 6 | 2.53 | .865 | 9.61 | .007 | .009 | .001 |
| Middle Childhood Model (*df* = 87) vs. |  |  |  |  |  |  |  |
| Whole Childhood Model | 6 | 3.41 | .756 | 9.13 | .001 | .003 | .010 |
| ***Additional Comparisons*** |  |  |  |  |  |  |  |
| Stability Model (*df* = 93) vs |  |  |  |  |  |  |  |
| its submodel with no effects of parenting  on ER patterns (*df* = 99) | 6 | 4.93 | .553 | −7.12 | −.005 | −.001 | −.017 |
| **Partner-Reported Combined Autonomy and Intimacy** |  |  |  |  |  |  |  |
| Stability Model (*df* = 93) vs. |  |  |  |  |  |  |  |
| Infancy Model | 6 | 3.12 | .793 | 9.11 | .002 | .007 | .005 |
| Middle Childhood Model | 6 | 2.09 | .911 | 9.91 | .006 | .002 | .000 |
| Whole Childhood Model | 12 | 7.13 | .849 | 17.03 | .009 | .027 | .014 |
| Infancy Model (*df* = 87) vs. |  |  |  |  |  |  |  |
| Middle Childhood Model | 0 |  |  | 0.80 | .004 | −.005 | −.005 |
| Whole Childhood Model | 6 | 3.96 | .682 | 7.92 | .007 | .020 | .009 |
| Middle Childhood Model (*df* = 87) vs. |  |  |  |  |  |  |  |
| Whole Childhood Model | 6 | 5.11 | .530 | 7.12 | .003 | .025 | .014 |
| ***Additional Comparisons*** |  |  |  |  |  |  |  |
| Stability Model (*df* = 93) vs |  |  |  |  |  |  |  |
| its submodel with no effects of parenting  on ER patterns (*df* = 99) | 6 | 18.54 | .005 | 6.10 | −.033 | −.001 | −.037 |
| its submodel with no effects of mothering  on ER patterns (*df* = 96) | 3 | 7.56 | .056 | 1.07 | −.026 | −.000 | −.003 |
| its submodel with no effects of fathering  on ER patterns (*df* = 96) | 3 | 9.88 | .020 | 4.54 | .001 | −.001 | −.036 |
| its submodel with no effects of fathering  on reappraisal (*df* = 97) | 4 | 9.48 | .050 | 0.78 | −.033 | −.000 | −.007 |
| its submodel with no effects of fathering  on suppression (*df* = 97) | 4 | 7.78 | .100 | −0.57 | −.026 | −.001 | −.001 |
| its submodel with no effects of fathering  on rumination (*df* = 97) | 4 | 17.06 | .002 | 8.34 | −.029 | .002 | −.037 |
| its submodel with no effects of fathering  on reappraisal and suppression (*df* = 98) | 5 | 9.70 | .084 | −0.87 | −.033 | −.001 | −.005 |
| *Note. N* = 885. The ∆AIC and ∆*R^2^* of reappraisal, suppression, and rumination were calculated by subtracting the estimates of the model above from the model below. In the model comparisons between the Infancy Model and Middle Childhood Model, scaled ∆χ2 tests were not calculated because these models were non-nested with each other. AIC = Akaike information criterion. | | | | | | | |
|  | | | | | | | |

Supplementary **Figure 8.** *The Final Self-Reported (A) and Partner-Reported (B) Combined Autonomy and Intimacy Models: Standardized Parameter Estimates and 95% Confidence Intervals.*


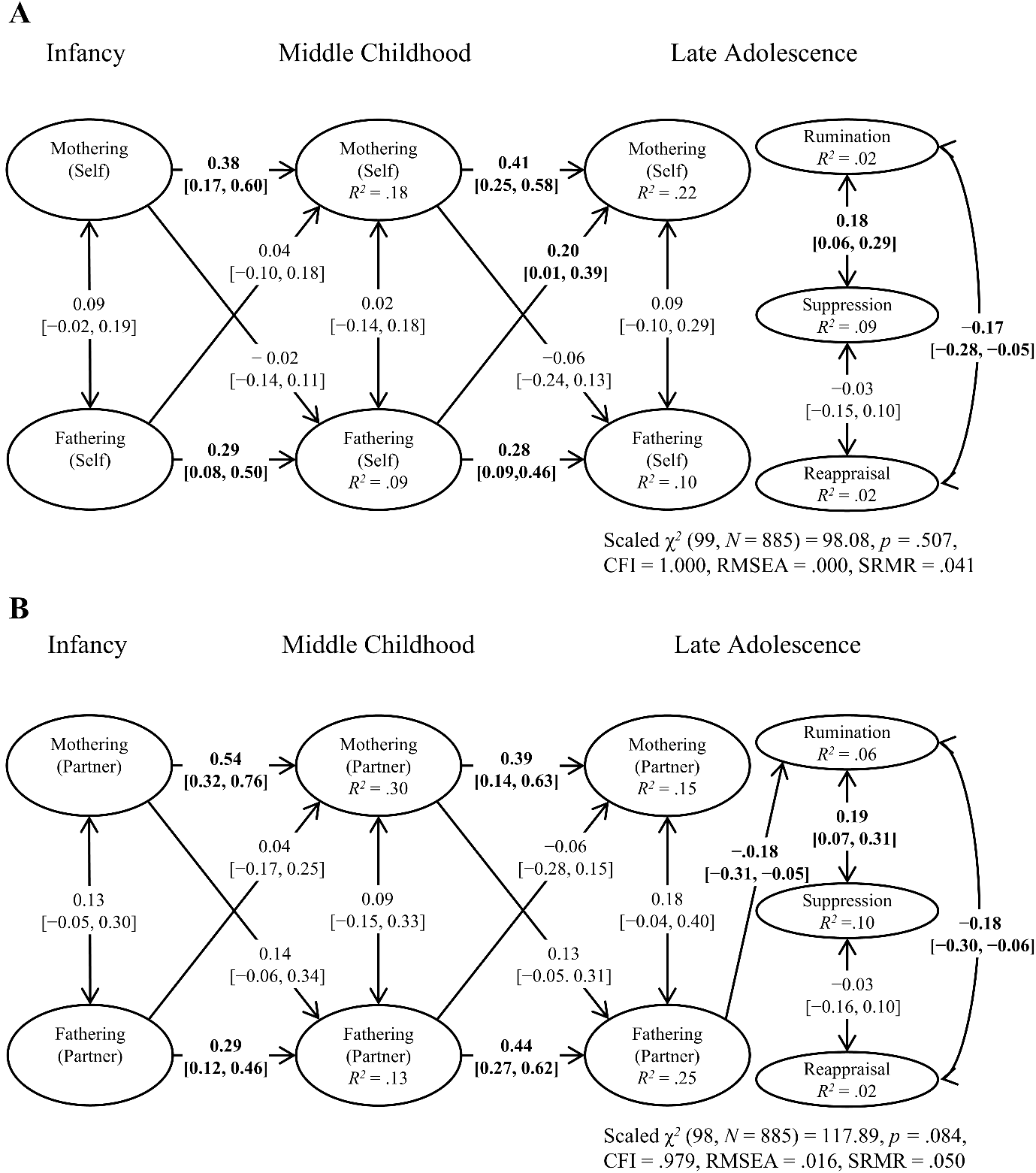


*Note.* Bolded values represent paths in which the 95% confidence interval did not contain zero. The factor loadings and error term correlations of indicators and the path coefficients of covariates are not shown. CFI = robust comparative fit index; RMSEA = robust root-mean-square error of approximation; SRMR = standardized root mean square residual.
